# Steroidal Pyrimidines and Dihydrotriazines as Novel Classes of Anticancer Agents against Hormone-Dependent Breast Cancer Cells

**DOI:** 10.3389/fphar.2017.00979

**Published:** 2018-01-10

**Authors:** Alexander M. Scherbakov, Alexander V. Komkov, Anna S. Komendantova, Margarita A. Yastrebova, Olga E. Andreeva, Valerii Z. Shirinian, Alakananda Hajra, Igor V. Zavarzin, Yulia A. Volkova

**Affiliations:** ^1^Department of Experimental Tumor Biology, N.N. Blokhin National Medical Research Center of Oncology, Moscow, Russia; ^2^N.D. Zelinsky Institute of Organic Chemistry, Russian Academy of Sciences, Moscow, Russia; ^3^Department of Chemistry, Visva-Bharati University, Santiniketan, India

**Keywords:** heterosteroids, steroidal pyrimidines, steroidal dihydrotriazines, breast cancer, prostate cancer, estrogen receptor α, anticancer drugs

## Abstract

Most breast and prostate tumors are hormone-dependent, making it possible to use hormone therapy in patients with these tumors. The design of effective endocrine drugs that block the growth of tumors and have no severe side effects is a challenge. Thereupon, synthetic steroids are promising therapeutic drugs for the treatment of diseases such as hormone-dependent breast and prostate cancers. Here, we describe novel series of steroidal pyrimidines and dihydrotriazines with anticancer activities. A flexible approach to unknown pyrimidine and dihydrotriazine derivatives of steroids with selective control of the heterocyclization pattern is disclosed. A number of 18-nor-5α-androsta-2,13-diene[3,2-d]pyrimidine, androsta-2-ene[3,2-d]pyrimidine, Δ^1, 3, 5(10)^-estratrieno[16,17-d]pyrimidine, and 17-chloro-16-dihydrotriazine steroids were synthesized by condensations of amidines with β-chlorovinyl aldehydes derived from natural hormones. The synthesized compounds were screened for cytotoxicity against breast cancer cells and showed IC_50_ values of 7.4 μM and higher. Compounds were tested against prostate cancer cells and exhibited antiproliferative activity with IC_50_ values of 9.4 μM and higher comparable to that of cisplatin. Lead compound **4a** displayed selectivity in ERα-positive breast cancer cells. At 10 μM concentration, this heterosteroid inhibited 50% of the E2-mediated ERα activity and led to partial ERα down-regulation. The ERα reporter assay and immunoblotting were supported by the docking study, which showed the probable binding mode of compound **4a** to the estrogen receptor pocket. Thus, heterosteroid **4a** proved to be a selective ERα modulator with the highest antiproliferative activity against hormone-dependent breast cancer and can be considered as a candidate for further anticancer drug development. In total, the synthesized heterosteroids may be considered as new promising classes of active anticancer agents.

## Introduction

Breast cancer is the most common cancer in women worldwide, with more than 1.5 million new cases recorded every year; it is also the fifth highest cause of cancer death (Nathan and Schmid, 2017). Estrogens are steroid hormones that play a critical role in the regulation of growth, differentiation, and metabolism of mammary cells, including malignant cells. Due to the ability of estrogens to significantly stimulate the growth of mammary cells, these hormones are involved in the progression of breast cancer. For more than 40 years, the antiestrogen tamoxifen (ICI 46474) is considered as the absolute leader in the endocrine therapy of hormone-dependent breast cancers (Jameera Begam et al., 2017). Tamoxifen belongs to selective estrogen receptor modulators (SERMs) (Cosman and Lindsay, 1999; Jameera Begam et al., 2017), which, in certain circumstances, perform the role of estrogen agonists or antagonists and modulate the effect of hormones in the target cells; otherwise, SERMs are also called estrogen agonists/antagonists. Due to convenient dosage forms for oral use, high efficiency, and low cost of the prolonged course of therapy, tamoxifen is considered as the “gold standard” for the treatment of patients with ERα-positive breast cancer. On the other hand, the effectiveness of tamoxifen may be limited by the development of resistance, an increased risk of endometrial cancer, and individual drug intolerance (Scherbakov et al., 2006; Ali et al., 2016; Traboulsi et al., 2017). This is why the development of novel classes of agents that effectively inhibit the growth of ERα-positive tumors and have no severe side effects is a challenge (Tryfonidis et al., 2016).

**Figure 1:**
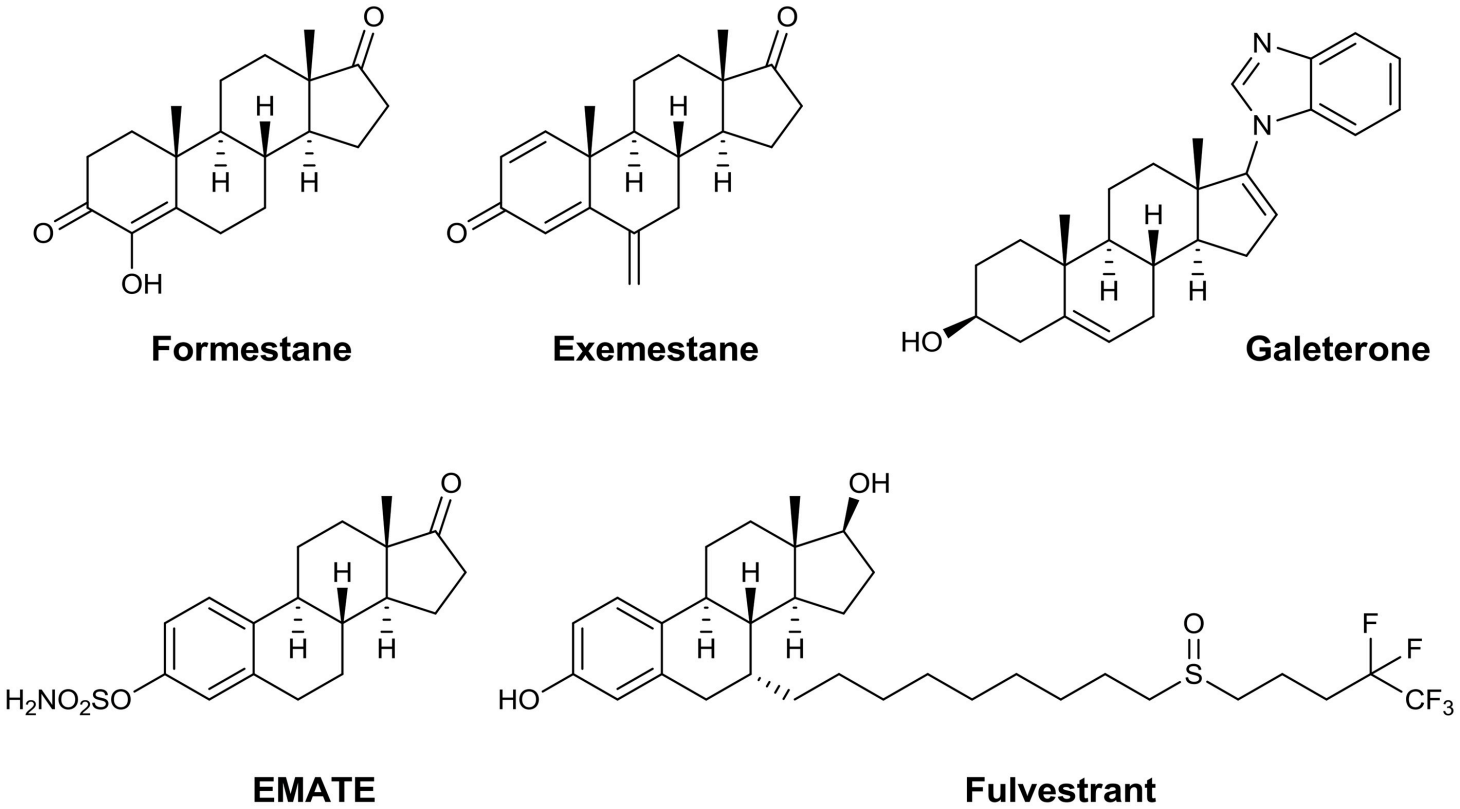
Steroidal anticancer agents.

Synthetic steroids encompass a wide range of compounds with various specific anticancer activities, e.g., aromatase inhibitors such as formestane and exemestane (Carlini et al., 2001), antiproliferative agents such as 2-methoxyestradiol (Lakhani et al., 2003), androgen signaling inhibitors such as galeterone and abiraterone (Bryce and Ryan, 2012), the SERM compound PSK3471, the steroid sulfatase inhibitor EMATE (Purohit and Foster, 2012), and the selective estrogen receptor degrader (SERD) fulvestrant (Nathan and Schmid, 2017; Figure 1).

Aromatase and steroid sulfatase inhibitors, SERDs, and SERMs synthetically derived from natural hormones are of great interest for the development of new breast cancer treatment regimens, especially for metastatic forms of the disease (Singer et al., 2006; Scherbakov et al., 2013; Secky et al., 2013; Boer, 2017; Kaklamani and Gradishar, 2017). Fulvestrant is an estrogen receptor degrader that binds with high selectivity to target cells, causes their degradation, resulting in the complete inhibition of the estrogen-mediated growth of breast cancer cells (Nathan and Schmid, 2017). First approved in the US in 2002, fulvestrant is not associated with tamoxifen-like agonist side effects, is not cross-resistant to tamoxifen or exemestane, and produces very high response rates in breast cancer patients. A combination of fulvestrant with other drugs seems to be very promising. In this regards, the combination of anastrozole and fulvestrant is superior to anastrozole alone or sequential anastrozole and fulvestrant for the treatment of ERα-positive metastatic breast cancer (Mehta et al., 2012).

In fulvestrant, the alkylsulfinyl moiety is attached to the endogenous estrogen receptor ligand, 17β-estradiol, at the 7-position, providing a structure similar to that of natural hormones but showing reverse biological activity. Recently, we have demonstrated that the modification of 17β-estradiol with imidazo[1,2-a]pyridine pendant at the 17α-position has the same effect (Rassokhina et al., 2016). 17α-Imidazopyridine-17β-methoxyestradiol showed remarkable effects as a selective ERα receptor modulator. In this study, we turned to an investigation of the structure—ERα-modulator activity relationship for two novel classes of heterosteroids possessing an *N*-heterocycle attached at the 16-position and fused to the A/D ring of the steroid core. We report the unique derivatives of the androstene and estrane series containing A/D-ring annulated pyrimidine or linked dihydrotriazine moieties. The antiproliferative potential of all synthesized compounds was evaluated in the MCF-7 and MDA-MB-231 breast cancer cell lines. The compounds were also tested toward two prostate cancer cell lines PC3 and 22Rv1. Taking into account activities of the compounds against hormone-dependent breast cancer ERα was analyzed as possible target for this series. Four compounds proved to be active as ERα antagonists. Steroidal dihydrotriazine **4a** was selected as the lead compound and analyzed by the ERα-reporter assay, immunoblotting, and docking simulation. Finally, the binding of compound **4a** to the estrogen receptor pocket was discussed using different docking models.

## Materials and methods

### Chemistry

#### General information

NMR spectra were acquired on Bruker Avance 600 and 300 spectrometers at 293, 303, and 333 K; the chemical shifts δ were measured in ppm relative to the solvent (^1^H: DMSO-d_6_, δ 2.50 ppm; ^13^C: DMSO-d_6_, δ 39.50 ppm). Splitting patterns are designated as s, singlet; d, doublet; t, triplet; q, quartet; m, multiplet; dd, double doublet; ddd, double double doublet; dt, doublet triplet. The coupling constants (*J*) are in Hertz. The structures of compounds were established using 1D NMR (^1^H, ^13^C) and 2D NMR (^1^H-^1^H COSY, ^13^C-^1^H HMBC, ^13^C-^1^H HSQC) spectroscopy. Infrared spectra were measured on a FT-IR spectrometer in KBr pellets. High-resolution mass spectra (HRMS) were measured using electrospray ionization (ESI) in positive ion mode (interface capillary voltage 4,500 V); the mass range was from m/z 50 to 3,000 Da; external/internal calibration was performed using an electrospray calibrant solution. A syringe injection was used for solutions in CH_3_CN (flow rate 3 ml/min). Nitrogen was applied as a dry gas and the interface temperature was set at 180°C. Melting points were measured on a Boetius capillary melting point apparatus and are uncorrected. Analytical thin-layer chromatography (TLC) was carried out on silica gel plates (silica gel 60 F254 aluminum supported plates); the visualization was accomplished with an UV lamp (254/365 nm) and using chemical staining with [Ce(SO_4_)_2_/H_2_SO_4_]. Column chromatography was performed on silica gel 60 (230–400 mesh, Merck). Androst-5-en-3β-ol-17-one-3β-acetate, 17β-hydroxy-5α-androstan-3-one, estrone, phosphorus oxychloride, guanidine salts, and acetimidamide hydrochloride were commercially available and were used as purchased. The spectroscopic data for steroidal chlorovinyl aldehydes **2a–c** are consistent with those reported previously (Komkov et al., 2015; Volkova et al., 2016). All reactions were carried out in freshly distilled and dry solvents.

#### 3-Hydroxy-2′-methyl-Δ^1,3,5(10)^-estratrieno[17,16-d]pyrimidine (**3a**)

Chloro-16-formyl-Δ^1,3,5(10)^-estratetraen-3-ol **2a** (113 mg, 0.36 mmol) was added to a suspension of acetamidine hydrochloride (51 mg, 0.54 mmol) and potassium carbonate (120 mg, 0.89 mmol) in DMF (4 mL). The mixture was stirred at 80°C for 9 h until the complete conversion of the intermediates (TLC monitoring). The resulting mixture was cooled to room temperature and diluted with water (30 mL). The precipitate that formed was filtered, dried, and washed with water (3 × 5 mL). The product was isolated by column chromatography using petroleum ether/ethyl acetate, 1:1, to obtain colorless solid (34 mg, 30% yield). R_*f*_ 0.27 (petroleum ether:EtOAc, 1:2; visualized by UV light at 254 nm); m.p. 264–266°C. ^1^H NMR (600 MHz, DMSO-d_6_), δ: 0.92 (s, 3H, 18-CH_3_), 1.35–1.41 (m, 1H, 7-CH_2_), 1.50–1.56 (m, 1H, 11-CH_2_), 1.61–1.69 (m, 2H, 8-CH_2_, 12-CH_2_), 1.70–1.76 (m, 1H, 14-CH), 1.90–1.95 (m, 1H, 7-CH), 2.14–2.18 (m, 1H, 12-CH_2_), 2.21–2.27 (m, 1H, 9-CH), 2.37–2.42 (m, 1H, 11-CH_2_), 2.48–2.54 (m, 1H, 15-CH_2_), 2.56 (s, 3H, 2′-CH_3_), 2.73–2.85 (m, 3H, 6-CH_2_, 15-CH_2_), 6.47 (s, 1H, 4-CH), 6.53 (dd, *J* = 2.4, 8.4 Hz, 1H, 2-CH), 7.07 (d, 1H, *J* = 8.4 Hz, 1-CH), 8.46 (s, 1H, 4-CH), 9.00 (br.s, 1H, OH). ^13^C NMR (125 MHz, DMSO-d_6_), δ: 17.1 (18-CH_3_), 25.5 (2′-CH_3_), 25.8 (11-CH_2_), 26.9 (7-CH_2_), 27.0 (15-CH_2_), 28.9 (6-CH_2_), 32.7 (12-CH_2_), 37.2 (8-CH), 43.7 (9-CH), 45.8 (13-C), 54.3 (14-CH), 112.8 (2-CH), 115.0 (4-CH), 125.8 (1-CH), 130.1 (10-C), 130.5 (16-C), 137.0 (5-C), 151.8 (4′-CH), 155.0 (3-C), 165.1 (2′-C), 181.0 (17-C). IR (KBr), cm^−1^: 3179 (OH), 2986, 2929, 2859 (CH), 1607, 1585, 1555, 1501 (C = C, C = N). HRMS (ESI) for C_21_H_25_N_2_O ([M+H]^+^): calcd 321.1961, found 321.1951.

#### 2′-Amino-3-hydroxy-Δ^1,3,5^(10)-estratrieno[17,16-d]pyrimidine (**3b**)

17-Chloro-16-formyl-Δ^1,3,5(10)^-estratetraen-3-ol **2a** (142 mg, 0.45 mmol) was added to a suspension of guanidine acetate (80 mg, 0.67 mmol) and potassium carbonate (180 mg, 1.34 mmol) in methanol (10 mL). The mixture was refluxed for 6 h until the complete conversion of the intermediates (TLC monitoring). The resulting mixture was cooled to room temperature and the solvent was removed under reduced pressure. The solid reside was washed with water (10 mL) and dried. The workup afforded the analytically pure product as colorless solid (129 mg, 89% yield). R_*f*_ 0.31 (CHCl_3_:MeOH, 5:0.2; visualized by UV light at 254 nm). The spectral data are consistent with those reported by Forgo and Vincze (2002); m.p. 285–287°C [m.p._lit_ (Forgo and Vincze, 2002) = 284–286°C]. ^1^H NMR (600 MHz, DMSO-d_6_), δ: 0.89 (s, 3H, 18-CH_3_), 1.32–1.36 (m, 1H, 7-CH_2_), 1.45–1.50 (m, 1H, 11-CH_2_), 1.54–1.64 (m, 2H, 8-CH_2_, 12-CH_2_), 1.65–1.68 (m, 1H, 14-CH), 1.86–1.90 (m, 1H, 7-CH_2_), 2.03–2.08 (m, 1H, 12-CH_2_), 2.20–2.25 (m, 1H, 9-CH), 2.31–2.38 (m, 2H, 11-CH_2_, 15-CH_2_), 2.62 (dd, *J* = 6.6, 14.4 Hz, 1H, 15-CH_2_), 2.69 (dt, *J* = 5.4, 16.2 Hz, 1H, 6-CH_2_), 2.77 (dt, *J* = 11.4, 16.2 Hz, 1H, 6-CH_2_), 6.36 (br.s, 2H, NH_2_), 6.40 (s, 1H, 4-CH), 6.45 (dd, *J* = 1.8, 8.4 Hz, 1H, 2-CH), 6.98 (d, 1H, *J* = 8.4 Hz, 1-CH), 8.00 (d, *J* = 1.8 Hz, 1H, 4-CH) (the signal of OH group was not observed in the ^1^H NMR spectrum). ^13^C NMR (125 MHz, DMSO-d_6_), δ: 17.1 (CH_3_), 26.0 (11-CH_2_), 26.6 (15-CH_2_), 27.1 (7-CH_2_), 29.1 (6-CH_2_), 32.9 (12-CH_2_), 37.4 (8-CH), 43.9 (9-CH), 45.8 (13-C), 54.4 (14-CH), 113.5 (2-CH), 115.6 (4-CH), 121.0 (16-C), 125.6 (1-CH), 128.2 (10-C), 136.7 (5-C), 152.6 (4′-CH), 157.5 (3-C), 162.9 (2′-C), 182.0 (17-C). IR (KBr), cm^−1^: 3361, 3181 (OH), 2930, 2858 (CH), 1637 (NH_2_), 1608, 1560 (C = C, C = N). HRMS (ESI) for C_20_H_24_N_3_O ([M+H]^+^): calcd 322.1914, found 322.1903.

#### 3β-Acetoxy-3′-methyl-5-androsteno[17,16-d]pyrimidine (**3c**)

3β-Acetoxy-17-chloro-16-formylandrosta-5,16-diene **2b** (130 mg, 0.34 mmol) was added to a suspension of acetamidine hydrochloride (65 mg, 0.69 mmol) and potassium carbonate (190 mg, 1.36 mmol) in DMF (5 mL). The mixture was stirred at 80°C for 6.5 h until the complete conversion of the intermediates (TLC monitoring). The resulting mixture was cooled to room temperature and the solvent was removed under reduced pressure. The crude product was isolated by column chromatography using petroleum ether/ethyl acetate, 1:2, to obtain colorless solid (52 mg, 40% yield). R_*f*_ 0.47 (petroleum ether:EtOAc, 1:2; visualized by UV light at 254 nm). The spectral data are consistent with those reported by Gogoi et al. (2013). m.p. 169–171°C [m.p._lit_ (Gogoi et al., 2013) = 165–167°C]. ^1^H NMR (600 MHz, DMSO-d_6at303K_), δ: 0.91 (s, 3H, 18-CH_3_), 1.05 (s, 3H, 19-CH_3_), 1.07–1.13 (m, 2H, 1-CH_2_, 9-CH), 1.49 (dt, *J* = 4.2, 12.6 Hz, 1H, 12-CH_2_), 1.53–1.65 (m, 4H, 2-CH_2_, 11-CH_2_, 12-CH_2_, 14-CH), 1.67–1.73 (m, 2H, 7-CH_2_, 11-CH_2_), 1.74–1.82 (m, 2H, 2-CH_2_, 8-CH), 1.86 (dt, *J* = 3.6, 13.2 Hz, 1H, 1-CH_2_), 1.99 (s, 3H, CH_3_CO), 2.05–2.12 (m, 1H, 7-CH_2_), 2.28–2.32 (m, 2H, 4-CH_2_), 2.47 (dd, *J* = 15.0 Hz, 1H, 15-CH_2_), 2.54 (s, 3H, 2′-CH_3_), 2.72 (dd, *J* = 6.6,15.0 Hz, 1H, 15-CH_2_), 4.43–4.48 (m, 1H, 3-CH), 5.39–5.40 (m, 1H, 6-CH), 8.43 (s, 1H, H-4′). ^13^C NMR (125 MHz, DMSO-d_6at303K_), δ: 16.7 (18-CH_3_), 18.9 (19-CH_3_), 20.0 (11-CH_2_), 21.0 (CH_3_), 25.4 (2′-CH_3_), 27.3 (2-CH_2_), 27.3 (15-CH_2_), 30.1 (8-CH), 30.6 (7-CH_2_), 32.4 (12-CH_2_), 36.3 (10-C), 36.3 (1-CH_2_), 37.7 (4-CH_2_), 45.4 (13-C), 49.8 (9-CH), 55.0 (14-CH), 73.1 (3-CH), 121.6 (6-CH), 130.5 (16-C), 139.8 (5-C), 151.8 (4′-CH), 165.1 (2′-C), 169.7 (CO), 180.8 (17-C). IR (KBr), cm^−1^: 2941, 2903, 2858 (CH), 1732 (CO), 1589, 1556 (C = C, C = N). HRMS (ESI) for C_24_H_33_N_2_O_2_ ([M+H]^+^): calcd 381.2537, found 381.2535.

#### 3β-Hydroxy-3′-methyl-5-androsteno[17,16-d]pyrimidine (**3d**)

A mixture of steroidal pyrimidine **3c** (50 mg, 0.13 mmol) and potassium carbonate (200 mg, 1.44 mmol) in MeOH (8 mL) was refluxed for 3 h until the complete conversion of the starting material (TLC monitoring). The resulting mixture was cooled to room temperature and the solvent was removed under reduced pressure. The crude product was washed with water (6 mL) and dried to get colorless solid (42 mg, 95% yield). R_*f*_ 0.28 (CHCl_3_:MeOH, 5:0.1; visualized by UV light at 254 nm); m.p. 182–183°C. ^1^H NMR (600 MHz, DMSO-d_6_ + CCl_4_), δ: 0.92 (s, 3H, 18-CH_3_), 1.03 (s, 3H, 19-CH_3_), 0.99–1.09 (m, 2H, 1-CH_2_, 9-CH), 1.32–1.38 (m, 1H, 2-CH_2_), 1.48 (dt, *J* = 4.2, 12.6 Hz, 1H, 12-CH_2_), 1.50–1.55 (m, 1H, 14-CH), 1.58–1.73 (m, 4H, 2-CH_2_, 7-CH_2_, 11-CH_2_), 1.74–1.82 (m, 2H, 1-CH_2_, 8-CH), 2.04–2.19 (m, 4H, 4-CH_2_, 7-CH_2_, 12-CH_2_), 2.46 (dd, *J* = 14.4 Hz, 1H, 15-CH_2_), 2.55 (s, 3H, 2′-CH_3_), 2.72 (dd, *J* = 14.4 Hz, 1H, 15-CH_2_), 3.22–3.32 (m, 1H, 3-CH), 5.28–5.30 (m, 1H, 6-CH), 8.39 (s, 1H, H-4′) (the signal of OH group was not observed in the ^1^H NMR spectrum). ^13^C NMR (125 MHz, DMSO-d_6_ + CCl_4_), δ: 16.7 (18-CH_3_), 19.1 (19-CH_3_), 20.0 (11-CH_2_), 25.3 (2′-CH_3_), 27.4 (15-CH_2_), 30.2 (8-CH), 30.7 (7-CH_2_), 31.3 (2-CH_2_), 32.5 (12-CH_2_), 36.3 (10-C), 36.8 (1-CH_2_), 42.2 (4-CH_2_), 45.4 (13-C), 50.1 (9-CH), 55.2 (14-CH), 69.9 (3-CH), 119.8 (6-CH), 130.4 (16-C), 141.6 (5-C), 151.5 (4′-CH), 164.9 (2′-C), 180.7 (17-C). IR (KBr), cm^−1^: 3378 (OH), 2965, 2938, 2858, 2818 (CH), 1599, 1554 (C = C, C = N). HRMS (ESI) for C_22_H_31_N_2_O ([M+H]^+^): calcd 339.2431, found 339.2431.

#### 2′-Amino-3β-hydroxy-5-androsteno[17,16-d]pyrimidine (**3e**)

3β-Acetoxy-17-chloro-16-formylandrosta-5,16-diene **2b** (122 mg, 0.32 mmol) was added to a suspension of guanidine acetate (58 mg, 0.48 mmol) and potassium carbonate (134 mg, 0.97 mmol) in methanol (10 mL). The resulting mixture was refluxed for 6 h until the complete conversion of the intermediates (TLC monitoring). The resulting mixture was cooled to room temperature. The precipitate that formed was filtered, washed with H_2_O (5 mL), and dried. The workup afforded the analytically pure product as colorless solid (92 mg, 84% yield). R_*f*_ 0.56 (CHCl_3_:MeOH, 5:0.2; visualized by UV light at 254 nm). The spectral data are consistent with those reported by Matsumoto et al. (2003). m.p. 342–344°C [m.p._lit_ (Matsumoto et al., 2003) = 308–312°C]. ^1^H NMR (600 MHz, DMSO-d_6at333K_), δ: 0.90 (s, 3H, 18-CH_3_), 1.03 (s, 3H, 19-CH_3_), 0.98–1.10 (m, 2H, 1-CH_2_, 9-CH), 1.34–1.42 (m, 1H, 2-CH_2_), 1.42–1.50 (m, 1H, 12-CH_2_), 1.44–1.52 (m, 1H, 14-CH), 1.55–1.83 (m, 6H, 1-CH_2_, 2-CH_2_, 7-CH_2_, 8-CH, 11-CH_2_), 1.99–2.09 (m, 2H, 7-CH_2_, 12-CH_2_), 2.09–2.22 (m, 2H, 4-CH_2_), 2.29 (dd, *J* = 6.0, 13.8 Hz, 1H, 15-CH_2_), 2.55 (dd, *J* = 6.0, 13.8 Hz, 1H, 15-CH_2_), 3.24–3.32 (m, 1H, 3-CH), 4.41 (br.s, 1H, OH), 5.30–5.32 (m, 1H, 6-CH), 6.11 (br.s, 2H, NH_2_), 7.98 (s, 1H, H-4′). ^13^C NMR (125 MHz, DMSO-d_6at333K_), δ: 16.4 (18-CH_3_), 18.8 (19-CH_3_), 19.9 (11-CH_2_), 26.6 (15-CH_2_), 30.1 (7-CH_2_), 30.5 (8-CH), 31.2 (2-CH_2_), 32.5 (12-CH_2_), 36.1 (10-C), 36.6 (1-CH_2_), 42.0 (4-CH_2_), 45.0 (13-C), 50.0 (9-CH), 55.1 (14-CH), 69.7 (3-CH), 119.6 (6-CH), 121.0 (16-C), 141.5 (5-C), 152.2 (4′-CH), 162.6 (2′-C), 181.5 (17-C). IR (KBr), cm^−1^: 3535 (OH), 3368, 3314, 3159 (NH_2_), 2935, 2893, 2844 (CH), 1647 (NH_2_), 1608, 1559 (C = C, C = N). HRMS (ESI) for C_21_H_30_N_3_O ([M+H]^+^): calcd 340.2383, found 340.2382. Anal. calcd for C_21_H_29_N_3_O:C, 74.30; H, 8.6; N, 12.38. Found: C, 73.83; H, 8.65; N, 12.15.

#### 2′-Amino-17β-hydroxy-5α-androstano[2,3-d]pyrimidine (**3f**)

3-Chloro-2-formyl-17β-formyloxy-5α-androstane **2c** (108 mg, 0.30 mmol) was added to a suspension of guanidine acetate (53 mg, 0.45 mmol) and potassium carbonate (124 mg, 0.90 mmol) in methanol (10 mL). The mixture was refluxed for 4 h until the complete conversion of the intermediates (TLC monitoring). The resulting mixture was cooled to room temperature. The precipitate that formed was filtered, washed with water (5 mL), and dried. The workup afforded analytically pure product as colorless solid (91 mg, 89% yield). R_*f*_ 0.67 (CHCl_3_:MeOH, 5:0.2; visualized by UV light at 254 nm). The spectral data are consistent with those reported by De Ruggieri et al. (1962). m.p. > 350°C (m.p._lit_ > 300°C). ^1^H NMR (600 MHz, DMSO-d_6_), δ: 0.68 (s, 3H, 18-CH_3_), 0.69 (s, 3H, 19-CH_3_), 0.78–0.84 (m, 1H, 9-CH), 0.86–0.95 (m, 2H, 7-CH_2_, 14-CH), 0.98–1.06 (m, 1H, 12-CH_2_), 1.14–1.29 (m, 2H, 6-CH_2_, 15-CH_2_), 1.32–1.42 (m, 3H, 8-CH, 11-CH_2_, 16-CH_2_), 1.49–1.56 (m, 3H, 5-CH, 6-CH_2_, 15-CH_2_), 1.58–1.64 (m, 1H, 11-CH_2_), 1.64–1.70 (m, 1H, 7-CH_2_), 1.77–1.82 (m, 1H, 12-CH_2_), 1.83–1.89 (m, 1H, 16-CH_2_), 2.16 (d, *J* = 15.6 Hz, 1H, 1-CH_2_), 2.24 (dd, *J* = 12.6, 18.0 Hz, 1H, 4-CH_2_), 2.43–2.56 (m, 2H, 4-CH_2_, 1-CH_2_), 3.47 (t, *J* = 8.4 Hz, 1H, 17-CH), 4.18 (br.s, 1H, OH), 5.90 (br.s, 2H, NH_2_), 7.91 (s, 1H, H-4′). ^13^C NMR (125 MHz, DMSO-d_6_), δ: 10.8 (18-CH_3_), 10.9 (19-CH_3_), 20.2 (11-CH_2_), 22.7 (15-CH_2_), 27.8 (6-CH_2_), 29.6 (16-CH_2_), 30.5 (7-CH_2_), 34.5 (10-C), 35.0 (8-CH), 35.2 (4-CH_2_), 36.3 (12-CH_2_), 38.4 (1-CH_2_), 40.8 (5-CH), 42.1 (13-C), 50.4 (14-CH), 53.0 (9-CH), 79.8 (17-CH), 116.7 (2-C), 157.9 (4′-CH), 161.6 (2′-C), 164.2 (3-C). IR (KBr), cm^−1^: 3322, 3169 (NH_2_), 2969, 2923, 2905, 2849 (CH), 1659 (NH_2_), 1596, 1561 (C = C, C = N). HRMS (ESI) for C_21_H_32_N_3_O ([M+H]^+^): calcd 342.2540, found 342.2538.

#### 16-(4,6-Dimethyl-1,2-dihydro-1,3,5-triazin-2-yl)-17-chloro-Δ^1,3,5(10), 16^-estratetraen-3-ol (**4a**)

17-Chloro-16-formyl-Δ^1,3,5(10)^-estratetraen-3-ol **2a** (100 mg, 0.32 mmol) was added to a suspension of acetamidine hydrochloride (150 mg, 1.3 mmol) and potassium carbonate (260 mg, 1.92 mmol) in DMF (4 mL). The mixture was stirred at 80°C for 8 h until the complete conversion of the intermediates (TLC monitoring). The resulting mixture was cooled to room temperature and diluted with water (30 mL). The precipitate that formed was filtered and washed with water (5 mL) and hot benzene (5 mL). The workup afforded the analytically pure product as colorless solid (40 mg, 32% yield). R_*f*_ 0.37 (petroleum ether:EtOAc, 1:2; visualized by UV light at 254 nm); m.p. 209–210°C. ^1^H NMR (600 MHz, DMSO-d_6_), δ: 0.84 (s, 3H, 18-CH_3_), 1.30–1.36 (m, 1H, 7-CH_2_), 1.37–1.50 (m, 3H, 8-CH, 11-CH_2_, 12-CH_2_), 1.58–1.62 (m, 1H, 14-CH), 1.75–1.83 (m, 2H, 7-CH_2_, 12-CH_2_), 1.80 (s, 3H, 4′-CH_3_), 1.82 (s, 3H, 6′-CH_3_), 1.95 (dd, *J* = 12.0, 14.4 Hz, 1H, 15-CH_2_), 2.14 (dd, *J* = 6.6, 14.4 Hz, 1H, 15-CH_2_), 2.18–2.22 (m, 1H, 9-CH), 2.31–2.36 (m, 1H, 11-CH_2_), 2.68–2.78 (m, 2H, 6-CH_2_), 5.35 (s, 1H, 2′-CH), 6.44 (d, *J* = 2.4 Hz, 1H, 4-CH), 6.51 (dd, 1H, *J* = 2.4, 9.0 Hz, 2-CH), 7.02 (d, 1H, *J* = 9.0 Hz, 1-CH), 9.03 (br.s, 1H), 9.37 (br.s, 1H). ^13^C NMR (150 MHz, DMSO-d_6_), δ: 15.0 (18-CH_3_), 20.2 (4′-CH_3_, 6′-CH_3_), 25.8 (11-CH_2_), 26.7 (7-CH_2_), 28.9 (15-CH_2_, 6-CH_2_), 33.6 (12-CH_2_), 36.9 (8-CH), 43.7 (9-CH), 47.9 (13-C), 53.1 (14-CH), 67.8 (2′-CH), 112.7 (2-CH), 115.0 (4-CH), 125.6 (1-CH), 130.2 (10-C), 135.4 (17-C), 137.0 (5-C), 138.6 (16-C), 151.6 (4′-C, 6′-C), 155.0 (3-C). IR (KBr), cm^−1^: 3198 (NH), 2929, 2857 (CH), 1703, 1611 (C = C, C = N), 1499, 1456, 1435, 1378, 1287, 1248. HRMS (ESI) for C_23_H_29_ClN_3_O ([M+H]^+^): calcd 398.1994, found 398.1995.

#### 3β-Acetoxy-16-(4,6-dimethyl-1,2-dihydro-1,3,5-triazin-2-yl)-17-chloroandrosta-5,16-diene (**4b**)

3β-Acetoxy-17-chloro-16-formylandrosta-5,16-diene **2b** (100 mg, 0.26 mmol) was added to a suspension of acetamidine hydrochloride (125 mg, 1.3 mmol) and potassium carbonate (220 mg, 1.6 mmol) in DMF (4 mL). The mixture was stirred at 80°C for 8 h until the complete conversion of the intermediates (TLC monitoring). The resulting mixture was cooled to room temperature and diluted with water (30 mL). The precipitate that formed was filtered, washed with water (5 mL), and dried. The crude product was purified by column chromatography using chloroform/MeOH, 10:1, to obtain colorless solid (45 mg, 30% yield). R_*f*_ 0.45 (petroleum ether:EtOAc, 1:2; visualized by UV light at 254 nm); m.p. 158–160°C. ^1^H NMR (600 MHz, DMSO-d_6_), δ: 0.84 (s, 3H, 18-CH_3_), 1.01 (s, 3H, 19-CH_3_), 1.02–1.11 (m, 2H, 1-CH_2_, 9-CH), 1.32 (dt, *J* = 4.2, 12.6 Hz, 1H, 12-CH_2_), 1.36–1.41 (m, 1H, 14-CH), 1.43–1.49 (m, 1H, 11-CH_2_), 1.51–1.65 (m, 4H, 2-CH_2_, 7-CH_2_, 8-CH, 11-CH_2_), 1.69–1.73 (m, 1H, 12-CH_2_), 1.76–1.84 (m, 2H, 1-CH_2_, 2-CH_2_), 1.79 (s, 3H, 4′-CH_3_), 1.81 (s, 3H, 6′-CH_3_), 1.88 (dd, *J* = 14.4, 15.0 Hz, 1H, 15-CH_2_), 1.92–1.97 (m, 1H, 7-CH_2_), 1.98 (s, 3H, CH_3_CO), 2.07 (dd, 1H, *J* = 6.6, 15.0 Hz, 15-CH_2_), 2.26–2.31 (m, 2H, 4-CH_2_), 4.42–4.48 (m, 1H, 3-CH), 5.32 (s, 1H, 2′-CH), 5.34–5.36 (m,1H, 6-CH), 9.40 (br.s, 1H, NH). ^13^C NMR (125 MHz, DMSO-d_6_), δ: 14.8 (18-CH_3_), 18.8 (19-CH_3_), 19.9 (11-CH_2_), 20.3 (4′-CH_3_, 6′-CH_3_), 20.9 (CH_3_COO), 27.3 (2-CH_2_), 29.2 (15-CH_2_), 29.9 (8-CH), 30.3 (7-CH_2_), 33.4 (12-CH_2_), 36.3 (1-CH_2_), 36.3 (10-C), 37.6 (4-CH_2_), 47.5 (13-C), 49.8 (9-CH), 53.6 (14-CH), 67.4 (2′-CH), 73.1 (3-CH), 121.8 (6-CH), 135.4 (17-C), 138.5 (16-C), 139.8 (5-C), 152.0 (4′-C, 6′-C), 169.7 (CO). IR (KBr), cm^−1^: 3183 (NH), 2945, 2857 (CH), 1735 (COO),1704, 1629 (C = C, C = N). HRMS (ESI) for C_26_H_37_ClN_3_O_3_ ([M+H]^+^): calcd 458.2569, found 458.2558.

#### 17β-Hydroxy-2-(4,6-dimethyl-1,2-dihydro-1,3,5-triazin-2-yl)-3-chloro-5α-androstane (**4c**)

3-Chloro-2-formyl-17β-formyloxy-5α-androstane **2c** (120 mg, 0.34 mmol) was added to a suspension of acetamidine hydrochloride (160 mg, 1.69 mmol) and potassium carbonate (280 mg, 2.0 mmol) in DMF (5 mL). The mixture was stirred at 60–65°C for 6 h until the complete conversion of the intermediates (TLC monitoring). The resulting mixture was cooled to room temperature and the solvent was removed under reduced pressure. The product was purified by column chromatography using chloroform/MeOH, 6:1, to obtain colorless solid (41 mg, 29% yield). R_*f*_ 0.30 (CHCl_3_:MeOH, 5:0.3; visualized by UV light at 254 nm); m.p. 210–212°C. ^1^H NMR (600 MHz, DMSO-d_6_), δ: 0.62 (s, 3H, 18-CH_3_), 0.68 (s, 3H, 19-CH_3_), 0.65–0.71 (m, 1H, 9-CH), 0.78–0.88 (m, 2H, 7-CH_2_, 14-CH), 0.90–0.96 (m, 1H, 12-CH_2_), 1.11–1.18 (m, 2H, 6-CH_2_, 15-CH_2_), 1.22–1.39 (m, 5H, 8-CH, 11-CH_2_, 16-CH_2_), 1.40–1.50 (m, 3H, 5-CH, 6-CH_2_, 15-CH_2_), 1.60 (d, *J* = 12.0 Hz, 1H, 7-CH_2_), 1.64 (d, *J* = 16.8 Hz, 1H, 1-CH_2_), 1.71 (d, *J* = 12.6 Hz, 1H, 12-CH_2_), 1.80 (s, 3H, 4′-CH_3_), 1.82 (s, 3H, 6′-CH_3_), 2.00–2.05 (m, 1H, 4-CH_2_), 2.06 (d, *J* = 16.8 Hz, 1H, 1-CH_2_), 2.14–2.19 (m, 1H, 4-CH_2_), 3.39–3.43 (m, 1H, 17-CH), 4.40 (br.s, 1H), 5.48 (s, 1H, 2′-CH), 9.50 (br.s, 1H). ^13^C NMR (150 MHz, DMSO-d_6_), δ: 11.1 (18-CH_3_), 11.6 (19-CH_3_), 20.3 (4′-CH_3_, 6′-CH_3_), 20.4 (11-CH_2_), 23.0 (15-CH_2_), 27.2 (6-CH_2_), 29.8 (16-CH_2_), 30.6 (7-CH_2_), 34.1 (10-C), 35.0 (8-CH), 36.4 (12-CH_2_), 38.1 (4-CH_2_), 39.1 (1-CH_2_), 42.4 (13-C), 42.5 (5-CH), 50.4 (14-CH), 53.1 (9-CH), 70.7 (2′-CH), 80.0 (17-CH), 123.0 (3-C), 134.0 (2-C), 152.2 (4′-C, 6′-C). IR (KBr), cm^−1^: 3205 (NH), 2926, 2872 (CH), 1708, 1665 (C = C, C = N), 1498, 1469, 1444, 1380, 1380, 1338, 1250. HRMS (ESI) for C_24_H_37_ClN_3_O ([M+H]^+^): calcd 418.2620, found 418.2610.

### Cell cultures and evaluation of inhibitory activity

The MCF-7 and MDA-MB231 human breast cancer cell lines and the PC3 and 22Rv1 prostate cancer cell lines were purchased from the ATCC collection. Cells were cultured in standard high glucose DMEM medium (Hyclone) supplemented with 10% fetal calf serum (FCS) (HyClone) and 0.1 mg/ml sodium pyruvate (Santa Cruz) at 37°C, 5% CO_2_ and 80–85% humidity (NuAir CO_2_ incubator). The cell growth was evaluated by the modified MTT (3-[4,5-dimethylthiazol-2-yl]-2,5-diphenyltetrazolium bromide) (Applichem) test (Iselt et al., 1989) as described in Volkova et al. (2016). Briefly, the cells were seeded at a density of 2.5 × 10^4^ cells per well in 24-well plates (Corning) in 900 μL of the medium. The tested compounds were dissolved in DMSO (Applichem) to 10 mM before experiments and then were diluted in the medium to the required concentrations. Compounds with different concentrations in 100 μL of the medium were added 24 h after the seeding, and the cells were grown for 72 h. After incubation with the compounds, the medium was removed, and the MTT reagent dissolved in the medium was added to the final concentration of 0.2 mg/mL to each well and incubated for 3 h. Then the cell supernatants were removed and the MTT formazan purple crystals were dissolved in 100% DMSO (350 μL per well). Plates were gently shaken and the absorbance was measured at 571 nm with a MultiScan reader (ThermoFisher). The viability of the cells was assessed after subtraction of the blank value (the absorbance in the well w/o cells) from all wells. Dose-response curves were analyzed by regression analysis using sigmoidal curves (Log(concentration) vs. normalized absorbance). The half maximal inhibitory concentrations (IC_50_) were determined with GraphPad Prism.

### Transient transfection and measurement of estrogen receptor α activity

To determine the transcriptional activity of the estrogen receptor α (ERα), MCF-7 cells were transfected with the plasmids containing luciferase reporter gene under the control of the promoter containing estrogen responsive elements. Assay was performed in steroid-free conditions (phenol red-free DMEM medium supplemented with 10% DCC serum). The reporter plasmid ERE-TK-LUC used in this work was kindly provided by Reid et al. (2003). The transfection was carried out for 24 h at 37°C using Metafectene (Biontex Laboratories). To control the efficiency and potential toxicity of the transfection, the cells were co-transfected with the β-galactosidase plasmid. The tested compounds were added to phenol red-free DMEM medium supplemented with 10% DCC serum. The luciferase activity was measured according to a standard protocol (Promega) using a Infinite M200 Pro luminometer (Tecan). The β-galactosidase activity was analyzed using a substrate, *p*-nitrophenyl β-D-galactopyranoside (ONPG). Briefly, cell lysates were mixed with 0.1 M phosphate buffer (pH 7.5) containing 1.0 mM MgCl_2_, 3.3 mM ONPG and 53 mM β-mercaptoethanol. After incubation for 1 h at 37°C, the absorbances at 405 nm were measured on the MultiScan FC reader (ThermoFisher). The luciferase activity was calculated in arbitrary units evaluated as the ratio of the luciferase activity to the galactosidase activity.

### Western blot analysis

The cells were removed from the dishes with 1.2 ml of phosphate buffer, twice washed, and incubated for 10 min on ice in the modified lysis buffer containing 50 mM Tris-HCl, pH 7.5, 0.5% Igepal CA-630, 150 mM NaCl, 1 mM EDTA, 1 mM DTT, 1 mM PMSF, 0.1 mM sodium orthovanadate and aprotinin, leupeptin, pepstatin (1 μg/mL each) as described earlier (Scherbakov et al., 2006). The protein content was determined by the Bradford method.

Cell lysates (40 μg protein) were separated in 10% SDS-PAGE under reducing conditions, transferred to a nitrocellulose membrane (SantaCruz), and processed according to the standard protocol. To prevent nonspecific absorption, the membranes were treated with 5% nonfat milk solution in TBS buffer (100 mM Tris, 150 mM NaCl, pH 7.5) with 0.1% Tween-20 and then incubated with primary antibodies overnight at 4°C.

Primary antibodies to ERα were purchased from Sigma-Aldrich (Merck); the antibodies against α-tubulin (Cell Signaling Technology) were added to standardize loading. Goat anti-rabbit IgGs (Jackson ImmunoResearch) conjugated to horseradish peroxidase were used as secondary antibodies. Signals were detected using the ECL reagent as described in Mruk and Cheng (2011) and an ImageQuant LAS4000 system (GE HealthCare). ImageJ software (NIH) was used for densitometry.

### Statistical analysis

Each biology experiment was repeated three times. Statistical analysis was performed using Microsoft Excel and GraphPad Prism. Results were expressed as mean ± S.D. (standard deviation value). Student's *t*-test was used to evaluate the significance of differences in comparisons. *P*-value of < 0.05 was considered statistically significant.

### Molecular docking analysis

*In silico* docking was performed using Autodock Vina (Trott and Olson, 2010) run through PyRx interface to manage the workflow and PyMol to visualize the results. Ligands were prepared by generating the energy-minimized 3D structures using ChemBioDraw3D followed by processing with Autodock Tools 1.5.4 to assign Gasteiger charges, merge nonpolar hydrogens, and set torsional bonds. Initial docking runs were performed within a 25–30 Å cubic search space surrounding the binding pocket, with solutions found using an exhaustiveness of 8, and output modes ranked according to binding affinity (BA). For a detailed comparison, multiple runs with a reduced search space were run with an increased exhaustiveness of 16 and 32. The Autodock Vina produced ligand poses with the best fit and strongest BA (global minima) using a stochastic algorithm to explore surfaces/pockets of the rigid macromolecule, through an iterative series of local optimizations evaluating both intermolecular (hydrophobic interactions, repulsions, hydrogen bonding, etc.) and intramolecular (torsion, rotational torque) factors. SAR insights are greatly aided by molecular docking analysis but must be taken as putative due to the rigid modeling of the protein target and the potential for conformational bias (Bissantz et al., 2010).

## Results

### Chemistry

**Scheme 1:**
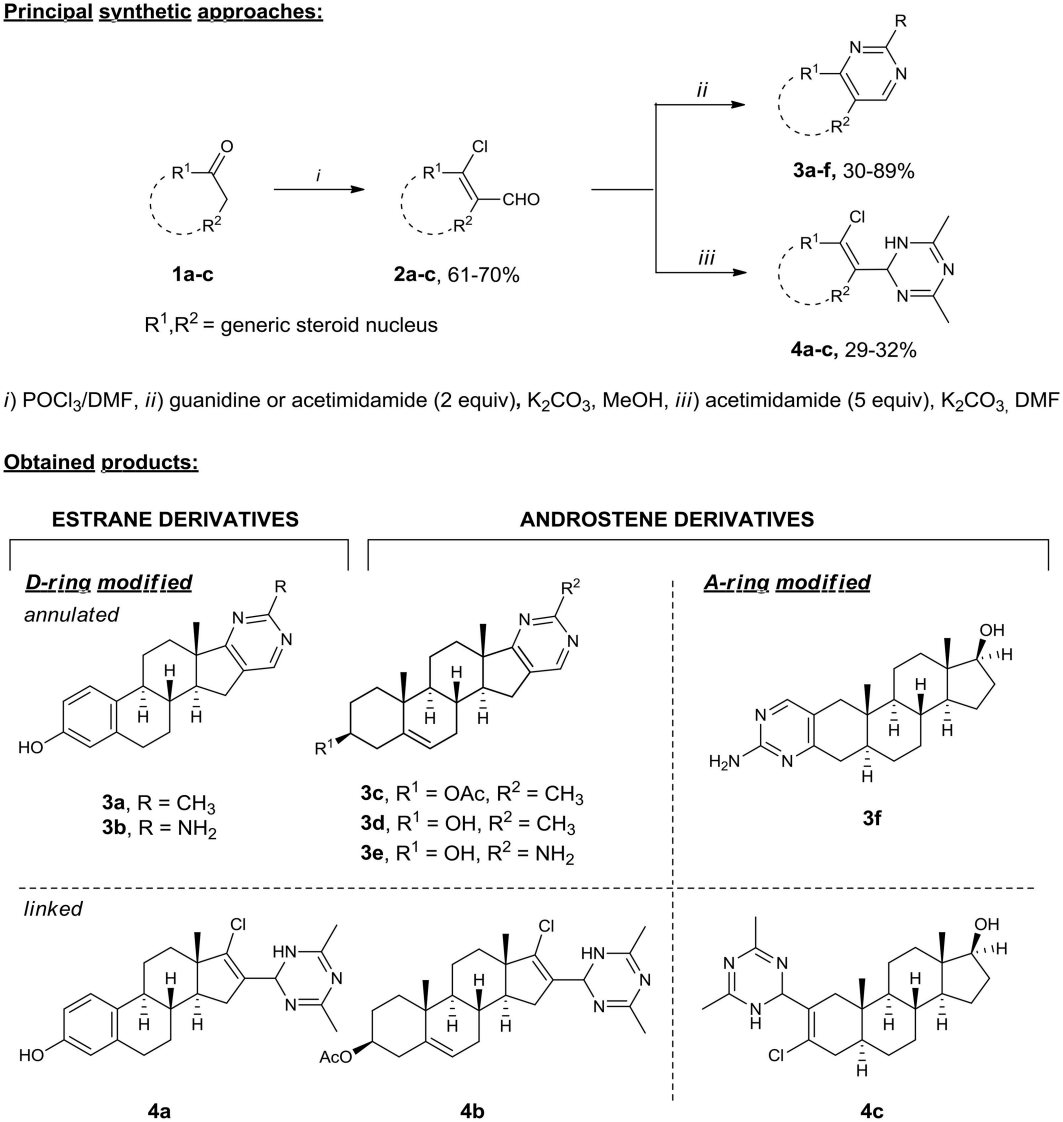
Synthesis of A-/D-ring functionalized azasteroids of the androstene and estrane series. Steroidal β-chlorovinyl aldehydes **2** as ambident elecrophiles easily undergo cyclizations with bis-nucleophilic guanidine and acetimidamide under mild reaction conditions (2 equiv excess, under reflux in methanol with potassium carbonate) providing A-/D-fused steroidal pyrimidines **3a–f** in 30–89% yields. The reaction of acetimidamide with 2 equiv excess of potassium carbonate in DMF produced heterosteroids **4a–c** containing the dihydrotriazine substituent at the 16-C or 2-C position of the steroid core in 29–32% yields.

Our interest in the preparation of structurally diverse heterosteroids lead to a need for a facile flexible strategy, in which a common intermediate can be used in a conjunctive fashion to form an array of *N*-heterocycles attached or fused to the steroid core. Hence, we turned to β-chlorovinyl aldehydes, which are readily available by the Vilsmeier–Haack reaction (Tasneem, 2003) and proved to be highly reactive ambident electrophiles (Bera et al., 2008; Bezboruah et al., 2013; Brockmeyer et al., 2014; Kroger et al., 2015). Recently, we have reported the synthesis of steroidal pyridazines (Komkov et al., 2015; Volkova et al., 2016), thiadiazoles (Zavarzin et al., 2013), and 4,5-disubstituted pyrimidines (Komendantova et al., 2017) via condensation of β-chlorovinyl aldehydes with bis-nucleophiles such as oxamic acid thiohydrazides and amidines. Based on these results, we accomplished the efficient synthesis of heterosteroids possessing a six-membered *N*-heterocycle attached or fused to the A/D ring of the steroid core starting from readily available materials. Thus, the synthesis of derivatives of the androstene and estrane series containing A-/D-ring annulated pyrimidine (Schemes 1, 3a–f) or linked dihydrotriazine (Schemes 1, 4a–c) moieties was accomplished starting from natural hormones **1a–c** (estrone, dihydrotestosterone, and dehydroepiandrosterone) by the general two-step sequence involving: (1) the Vilsmeier–Haack reaction giving steroidal β-chlorovinyl aldehydes **2a–c**, (2) the condensation of the former with amidines (Scheme 1).

The heterocyclization pattern was directed to dihydrotriazines by increasing amidine excess up to 4–5 equivs. The dihydrotriazine ring was constructed from two acetimidamide molecules and one steroid molecule *via* the nucleophilic attack of two amidine molecules on the formyl group. The reaction of acetimidamide with potassium carbonate in DMF produced heterosteroids **4a–c** containing the dihydrotriazine substituent at the 16-C or 2-C position of the steroid core in 29–32% yields. The structural assignments for all compounds **3a–f** and **4a–c** were confirmed by 2D NMR (^1^H-^1^H COSY, ^13^C-^1^H HMBC, and ^13^C-^1^H HSQC, see Supplementary Material) techniques and HRMS.

### Antitumor evaluation

#### Cytotoxic effects against breast and prostate cancer cells

The antiproliferative activity of all the synthesized compounds was evaluated against the human estrogen-responsive MCF-7 breast cancer cell line and ERα-negative MDA-MB231 cells using the MTT assay (Figures S1, S2). Cisplatin, a standard chemotherapy drug, was used as the reference compound. All compounds were also tested for cytotoxicity toward prostate cancer cells (Figures S3, S4). AR-negative PC3 cells and AR-positive 22Rv1 cells were used in this assay. The corresponding inhibitory concentrations IC_50_ (IC_50_ is the half maximal inhibitory concentration) are given in Table 1.

**Table 1:** Antiproliferative activity of the synthesized heterosteroidal compounds.

**Entry**	**IC**_**50**_, μ**M**			
	**Breast cancer**		**Prostate cancer**	
	**MCF-7**	**MDA-MB231**	**PC3**	**22Rv1**
**3a**	NA	NA	NA	NA
**3b**	NA	NA	NA	NA
**3c**	21.6 ± 2.2	NA	NA	NA
**3d**	12.0 ± 1.4	NA	NA	NA
**3e**	14.9 ± 1.5	NA	NA	NA
**4a**	7.4 ± 0.9	14.7 ± 1.6	13.5 ± 1.5	11.7 ± 1.4
**4b**	11.2 ± 1.4	12.2 ± 1.4	12.9 ± 1.4	9.4 ± 1.0
**4c**	14.8 ± 1.6	19.1 ± 2.1	22.7 ± 2.4	18.1 ± 1.9
Cisplatin (reference drug)	6.5 ± 0.9	14.1 ± 1.7	4.9 ± 0.7	9.8 ± 1.1

*NA indicates that the compound does not inhibit the growth by 50% at concentrations lower than 25 μM*.

Most of the tested heterosteroids showed remarkable anticancer activity against ERα-positive MCF-7 cancer cells. Estranes **3a,b** containing the D-ring-fused pyrimidine moiety proved to be inactive, while their androstene analogs **3c–e** were active with the IC_50_ values in the range of 12.0–21.6 μM. It is remarkable that the IC_50_ value for compound **3d** containing the 3-OH group is higher than that for compound **3c** possessing the 3-OAc protected group. The solubility of steroidal A-ring annulated pyrimidine **3f** in DMSO is too low to perform the MTT assay.

Steroidal dihydrotriazines **4a–c** proved to be more active against MCF-7 cancer cells compared to steroidal fused pyrimidines. Androstene derivative **4c** bearing the dihydrotriazine moiety at C-2 had the IC_50_ value of 14.8 μM, while the IC_50_ value for compound **4b** modified at 16-C reached 11.2 μM. The 16-C dihydrotriazine-modified estrane **4a** was shown to be the most active derivative. Moreover, only steroidal dihydrotriazines **4a**, **4b**, and **4c** were active against ERα-negative MDA-MB231 cells; their IC_50_ values vary in the range of 12.2–19.1 μM.

All compounds were tested against 22Rv1 and PC3 prostate cancer cells. Among them, compounds **4a**, **4b**, and **4c** displayed antiproliferative activity. Estrane derivative **4a** inhibited the growth of PC3 and 22Rv1 prostate cancer cells with IC_50_ of 13.5 and 11.7 μM, respectively, while androstene derivative **4b** displayed cytotoxicity comparable to that of cisplatin in hormone-dependent 22Rv1 prostate cancer cells. Androstene derivative **4c** was less active against prostate cancer cells than compounds **4a** and **4b**, and revealed the IC_50_ value about 20 μM (Table 1).

#### ERα activity and immunoblotting

Considering indicated antiproliferative activity of compounds in ERα-positive MCF-7 breast cancer cells, ERα was analyzed as a possible target for these synthetic steroids. For this purpose the luciferase reporter assay was used to determine ERα activity in MCF-7 cells. The ERα-mediated reporter constructs were provided to express luciferase under the control of the promoter containing estrogen responsive elements (ERE-TK-LUC). Thus, ERα activity was correlated to luciferase activity measured in treated or control cells.

**Figure 2:**
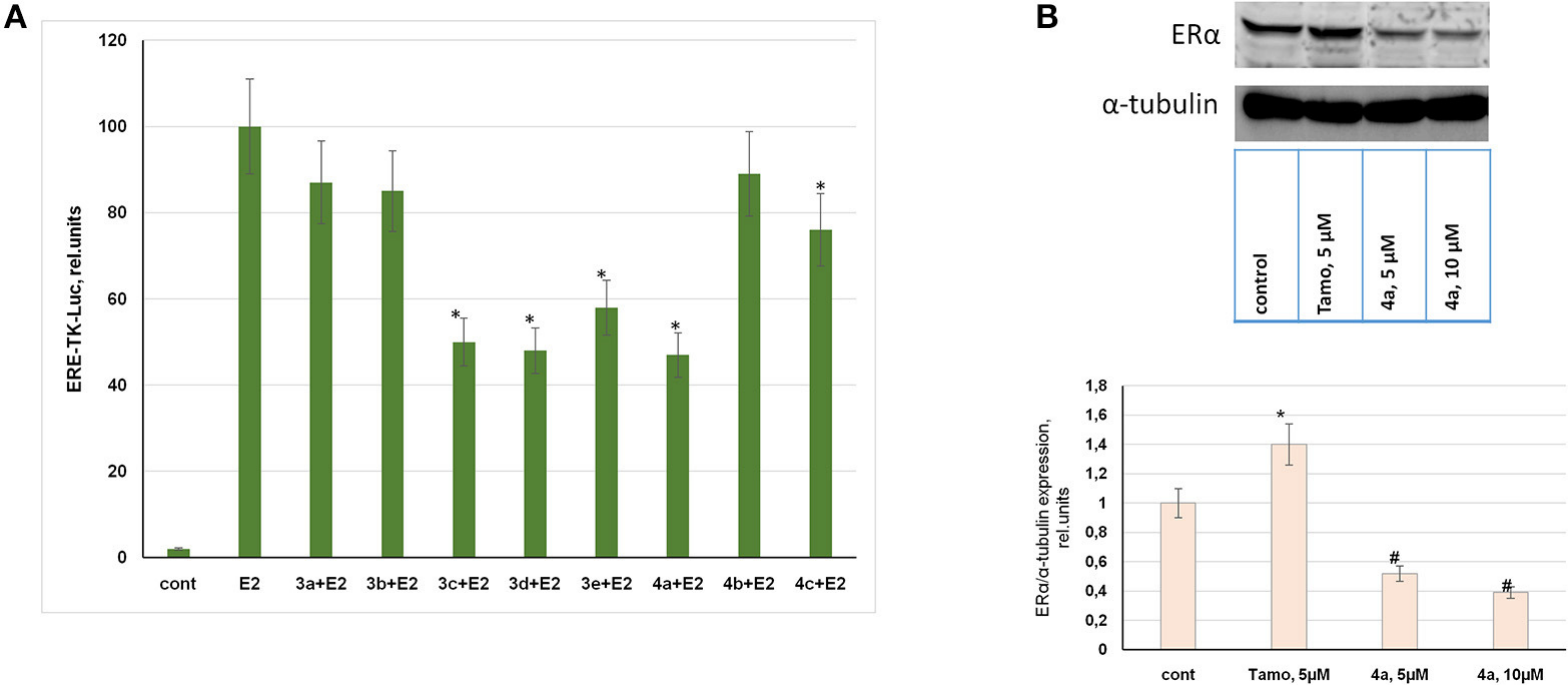
Evaluation of ERα activity and expression. **(A)** MCF-7 human breast cancer cells were transfected with the ERE-TK-LUC plasmid containing the luciferase reporter gene under the estrogen responsive element (ERE) and co-transfected with β-galactosidase plasmid. The media was removed 24 h after transfection and 10 nM of 17β-estradiol was used to induce ERα activity. Synthesized compounds at 10 μM concentration or vehicle control (cont) was added to phenol-free DMEM supplemented with 10% steroid-free serum (Hyclone). The luciferase and β-galactosidase activities were determined in 24 h. The luciferase activity was calculated in relative units evaluated as the ratio of the luciferase activity to the galactosidase activity. ^*^P < 0.05 vs. E2-treated cells. **(B)** MCF-7 cells were treated with or without tamoxifen or compound **4a**. The cell samples were subjected to Western blot analysis of ERα and α-tubulin as the loading control; Densitometry for ERα/α-tubulin ratio was carried out using ImageJ software with the densitometry protocol provided by The University of Queensland. ^*^P < 0.05 vs. control cells, ^#^*P* < 0.05 vs. tamoxifen-treated and control cells.

As can be seen in Figure 2A, estranes **3a,b** containing the D-ring-fused pyrimidine moiety did not inhibit ERα activity at 10 μM concentration. Moreover, these **3a** and **3b** stimulated ERα activity at low (10 nM) concentration acting as partial receptor agonists (Table S1). Androstene derivative **4b** proved to be inactive as ERα inhibitor. Compound **4c** showed weak inhibitory activity, while steroids **3c, 3d, 3e**, and **4a** highly inhibited E2-mediated ERα activity at 10 μM concentration. These compounds showed no ERα agonist activity in the luciferase reporter assay (Table S1).

Taking into consideration the two-fold gain in cytotoxicity of compound **4a** against ERα-positive breast cancer cells vs. ERα-negative cells and its high activity as ERα inhibitor we performed immunoblotting of ERα in MCF-7 cells. The data obtained by immunoblotting confirmed that compound **4a** exerted ERα inhibitory activity. The incubation of MCF-7 cells with compound **4a** resulted in the partial suppression of ERα expression, as can be seen in Figure 2B. Tamoxifen was used as the standard reference drug and its application resulted in an increase in ERα expression, which may be attributed to tamoxifen-induced stabilization of inactive ERα in the cell cytoplasm as discussed in Wijayaratne and McDonnell (2001). As compared with tamoxifen, compound **4a** was found to be active as the partial ERα downregulator (Figure 2B).

### Estrogen receptor docking analysis

**Figure 3:**
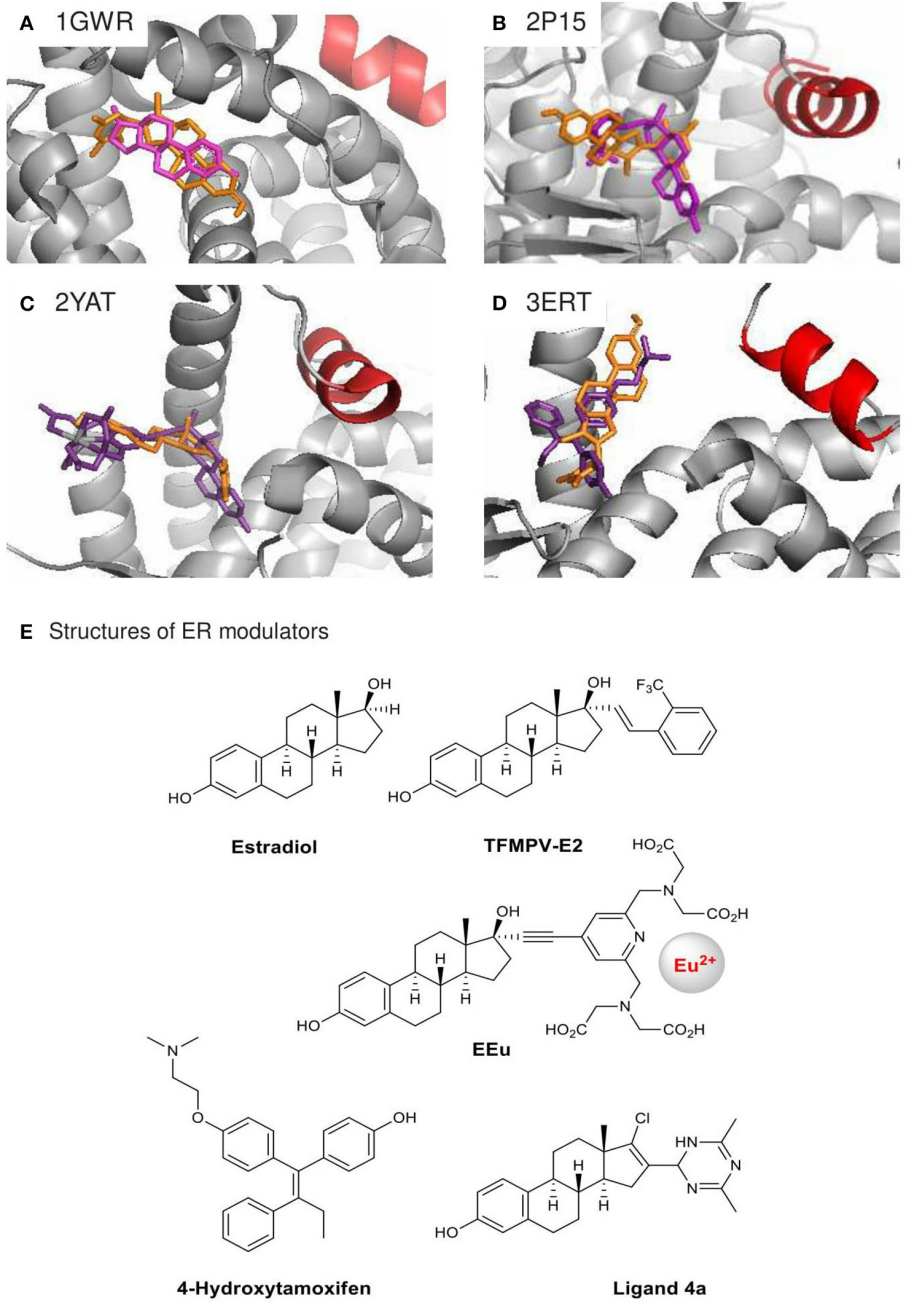
Crystal structures of agonist-bound ERα 1GWR **(A)**, 2P15 **(B)**, 2YAT **(C)** and antagonist-bound 3ERT **(D)** used in the docking analysis of ER modulator **4a**. Their original ligands **(E)** are in purple, modulator **4a** in orange, and helix 12 in red. Only the highest ranked poses with the strongest BA were selected: **(A)** +0.1 kcal/mol, **(B)** −8.1 kcal/mol, **(C)** −8.9 kcal/mol, **(D)** −8.9 kcal/mol.

In order to gain insight into the structural basis of the observed ERα inhibitory effects of compound **4a**, we performed *in silico* docking analysis using Autodock Vina. Low-energy binding poses were generated by evaluating the combined energetic contributions of torsion, steric repulsion, hydrogen bonding, and hydrophobic interactions between the ligand and the protein binding pocket. Using the crystal structures of ERα in complexes with estradiol [PDB: 1GWR (Warnmark et al., 2002), Figure 3A] and the weak agonists 17α-modified estradiol analogs TFMPV-E2 [PDB: 2P15 (Nettles et al., 2007), Figure 3B] and EEu [PDB: 2YAT (Li et al., 2011), Figure 3C], we found that the steroidal moiety of compound **4a** in the docked poses differs from the estradiol moieties of the original ligands (Table 1, Figures 3A–C,E).

Compound **4a** was found to be too big for the accommodation in the estradiol-binding pocket of ERa in the structure 1GWR. Meanwhile, the ligands TFMPV-E2 (PDB: 2P15) and EEu (PDB: 2YAT) are known to modulate the dynamics of the ERα helix 12 (shown in red, Figures 3A–D), resulting in an increase in the ligand-binding pocket surface of ER without changing the shape of the ligand-binding domain of ER due to the presence of bulky substituents at the 17α position of the estradiol core (Eignerova et al., 2010; Gryder et al., 2013). Compound **4a** bearing the bulky dihydrotriazine ring at the 16-position is docked against the ERα crystal structures 2P15 and 2YAT with reasonable binding affinity (−8.1 and −8.9 kcal/mol, respectively). However, the major binding modes of compound **4a** for 2P15 and 2YAT are as follows: the A-ring phenolic hydroxyl group points away the binding pocket of ER and the dihydrotriazine moiety points inward the binding pocket. The critical hydrogen-bonding interactions between the steroid estradiol, TFMPV-E2 and the A-ring phenolic hydroxyls of EEu with Arg-394/Glu-353 were not found for compound **4a** (Figures 3A–C), which can be attributed to steric hindrance caused by the bulky substituent at the 16-position and spatial aliasing of the estrane core due to the addition of the C_16_ = C_17_ double bond.

Alternatively, compound **4a** docked against the crystal structure of ERα in complex with the ER antagonist 4-hydroxytamoxifen (PDB: 3ERT, Figures 3D,E) is able to fill the hydrophobic space and latch onto Arg-394/Glu-353. Tamoxifen prevents the helix 12 from closing properly upon the binding pocket of the ligand-binding domain, while compound **4a** can extend the steroidal moiety through the opening left by the displaced helix 12. The D ring of steroid **4a** modified at the 16-and 17-positions can go inside the pocket potentially stabilized by polar interactions with TRP-383, Phe-404, Leu-387, Met-388, and Leu-391. These docked structures may reflect the most probable mode of binding. Although the direct comparisons are speculative, our docking outputs are supported by our observation with compound **4a** in ER luciferase reporter assays. The antagonist effect of compound **4a** is interesting, because D-ring modifications of estradiol commonly result in weak ER agonists (Yang et al., 2000; Kreis et al., 2001).

## Discussion

The initial synthesis of annulated steroidal pyrimidines by the groups of Clinton (Ackerman et al., 1964) and Ruggieri (De Ruggieri and Gandolfi, 1965; De Ruggieri et al., 1965, 1966a,b) dates back to the mid-1960s. Their synthetic approach was based on condensation of guanidines with activated α,β-unsaturated ketones, primarily β-enol ethers, and have become widely applied in chemistry of steroids due to a great diversity of obtainable products (Romo et al., 1968; Bajwa and Sykes, 1978; Hajos and Snatzke, 1989; Mallamo et al., 1992; Hasan et al., 1995; Forgo and Vincze, 2002). Unfortunately, this method suffers from drawbacks, such as harsh reaction conditions, moderate yields and high labor content/cost of preparing starting materials. Therefore, over the years a considerable effort has been directed toward the development of alternative methods for steroidal pyrimidines synthesis. Improved solid phase protocol of β-enol ethers heterocyclization was elaborated toward synthesis of steroidal A-ring fused pyrimidines (Barthakur et al., 2009). Although there is an example of condensation using β-enamino ketones (Xu et al., 2012). Boruah group have developed a range of methods, among which are three-component condensation of steroidal ketones with aromatic aldehydes and amidine derivatives in presence of potassium tert-butoxide (Saikia et al., 2014), Pd(OAc)_2_-catalyzed hetrocyclization of steroidal β-halo-α,β-unsaturated aldehydes with amidines (Gogoi et al., 2013) and SmCl_3_-catalyzed condensation of β-formyl enamide with urea under microwave irradiation (Barthakur et al., 2007). Baran group reported synthesis of 4,5-disubstituted pyrimidines from steroidal ketones and formamidine acetate (Baran et al., 2006). Here we have achieved high-yielding syntheses of novel A- and D-rings annulated steroidal pyrimidines via developed by us metal-free condensations of β-chlorovinyl aldehydes with amidines (Komendantova et al., 2017). These reactions are efficiently occur under mild conditions, with the added advantage that heterocyclization pattern can be easily switch to steroidal dihydrotriazines, previously unexplored class of heterosteroids.

Steroidal pyrimidines may be considered as promising compounds for the design of novel antitumor drugs. This line of research has been extensively developed in recent years. In 2017, Ke et al. designed novel steroidal[17,16-d]pyrimidines derived from dehydroepiandrosterone and evaluated their *in vitro* inhibitory activity against liver and gastric cancer cells (Ke et al., 2017). Briefly, 16 steroidal[17,16-d]pyrimidines derived from dehydroepiandrosterone were designed and synthesized via a sequence transformation, and their activities were assessed by MTT. Ke et al. found that some of these heterocyclic steroidal[17,16-d]pyrimidines showed antiproliferative activities against HepG2, Huh-7, and SGC-7901 cell lines compared to the reference 5-fluorouracil. Eight novel compounds synthesized by Ke et al. exhibited excellent inhibitory activities against all three cell lines with 70–82% growth inhibition at the concentration of 40 μg/mL. Thus, steroidal[17,16-d]pyrimidines might be used as promising compounds for discovery of novel anticancer drugs for treatment of liver and gastric cancers.

Other promising steroidal pyrimidines were discussed by Ali et al. (2017). The antitumor activity of the B-ring fused steroidal pyrimidines was tested *in vitro* against the MDA-MB231, HeLa, and HepG2 cancer cell lines and the non-cancer normal cell line PBMCs (peripheral blood mononuclear cells) by the standard MTT assay. The compounds showed moderate to good activity and proved to be nontoxic to normal PBM cells. One of the synthesized compounds was found to be active against all three cancer cell lines but more specific against the MDA-MB231 cells with IC_50_ of about 9 μM, which is similar to our data on the activity of the compounds against breast cancer cells. Finally, the authors discussed the ability of steroidal compounds to interact with the protein HSA involved in drug delivery.

Metastatic bone tumors occur at particularly high rates in cancers of the breast, prostate, and lung, accounting more than 70% of all patients. Treatment of skeletal metastasis and development of new specific “blockers” of bone resorption are relevant. Pyrimidine-fused betulinic acid may be considered as promising compounds for the design of novel inhibitors of osteoclast differentiation and bone resorption. Jun Xu et coworkers synthesized over 20 heterocyclic ring-fused betulinic acid derivatives and evaluated their inhibition on RANKL-induced osteoclast formation in preosteoclast RAW264.7 cells (Xu et al., 2012). Some compounds exhibited potent inhibitory activity on RANKL-induced osteoclast formation by TRAP assay.

The elucidation of the mechanism of action of compounds in target cells and understanding of their common metabolism in human body are of interest. The structural optimization will be performed and the molecular mechanism of novel steroidal pyrimidines will be investigated in due course. On the other hand, the activity of steroidal dihydrotriazines against cancer cells is less well known described, and our study is very relevant.

## Conclusion

Here, we describe novel series of steroidal anticancer agents. In summary, this study demonstrates that the cyclization of steroidal β-chlorovinyl aldehydes with bis-nucleophilic amidines provides an easy approach to various novel heterosteroids. Natural hormones **1a–c** (estrone, 3β-acetoxyandrostene, 3-keto-17β-hydroxyandrostane) were transformed into the corresponding A- and D-modified steroidal pyrimidines and dihydrotriazines in moderate to high yields (29–89%) using a two-step sequence involving the Vilsmeier–Haack reaction and condensation with amidines, such as guanidine and acetimidamide. The new compounds showed remarkable cytotoxic activity against breast and prostate cancer cells. Furthermore, lead compounds demonstrated selectivity toward ERα in MCF-7 breast cancer cells. Compound **4a** inhibits 50% of ERα activity at its cytotoxic concentration. Using immunoblotting, partial ERα downregulation was observed in compound **4a**-treated MCF-7 cells. Docking approaches confirmed the ability of compound **4a** to bind to ERα. Thus, compound **4a** may be considered as a candidate for future anticancer drug design, in particular, for ERα-positive breast cancers.

Despite a limited number of compounds in series, it provides significant novel insight into the structure–activity relationship of heterosteroids as anticancer agents. Biological studies show that annulation of androst-5-ene core with pyrimidine is efficient for development of novel selective compounds for treatment of hormone-dependent breast cancer. Moreover, installation of dihydrotriazine pendant at A- and D-rings of estrane and androst-5-ene cores results in strong antiproliferative activities against breast and prostate cancer cells comparable with cisplatine. The two-fold gain in cytotoxicity of 16-C dihydrotriazine-modified estrane against ERα-positive breast cancer cells vs. ERα-negative cells and its high activity as ERα inhibitor were shown while similar androstene derivative was less selective.

These results offer new knowledge about the binding site and receptor flexibility of ERα. The described heterosteroids will be useful lead agents for the development of more potent and selective SERMs.

## Author contributions

AS carried out the immunoblotting, performed the statistical analysis and the transient transfection, drafted and prepared the manuscript for submission, worked with cell cultures; AVK synthesized steroidal compounds; ASK synthesized steroidal compounds, prepared the supporting information; MY worked with cell cultures; OA performed the reporter analysis, worked with plasmids; VS managed the project; IZ managed the project; AH managed the project; YV wrote the manuscript, conceived of the study, *in silico* analysis, and managed the project; All authors read and approved the final manuscript.

### Conflict of interest statement

The authors declare that the research was conducted in the absence of any commercial or financial relationships that could be construed as a potential conflict of interest.

## Abbreviations

SERM: Selective estrogen receptor modulator
SERD: selective estrogen receptor degrader
ER: estrogen receptor
MTT: methylthiazolyldiphenyl-tetrazolium bromide
ERE: estrogen response element
DCC serum: dextran-coated charcoal-treated serum
AR: androgen receptor
E2: 17β-estradiol.
