# Supplementary material for: Steroidal Pyrimidines and Dihydrotriazines as Novel Classes of Anticancer Agents against Hormone-Dependent Breast Cancer Cells
Source: Front Pharmacol. 2018 Jan 10;8:979. doi: 10.3389/fphar.2017.00979 (PMC5767602; doi:10.3389/fphar.2017.00979)
Supplement: Supplementary file 1 [file SupplementaryMaterial.pdf]

STEROIDAL PYRIMIDINES AND DIHYDROTRIAZINES AS NOVEL CLASSES OF ANTICANCER AGENTS AGAINST HORMONE-DEPENDENT BREAST CANCER CELLS

Alexander M. Scherbakov[1*], Alexander V. Komkov[2], Anna S. Komendantova[2], Margarita A. Yastrebova[1], Olga E. Andreeva[1], Valerii Z. Shirinian[2], Alakananda Hajra,[3] Igor V. Zavarzin[2], Yulia A. Volkova[2*]

[1]*Department of Experimental Tumor Biology, N.N. Blokhin National Medical Research Center of Oncology, Kashirskoye shosse 24, 115478 , Moscow, Russia*

[2] *N.D. Zelinsky Institute of Organic Chemistry, Russian Academy of Sciences, Leninsky prosp. 47, 119991 Moscow, Russia*

[3] *Department of Chemistry, Visva-Bharati (A Central University), Santiniketan, India*

^{1}H NMR (DMSO-d6, 600 MHz) spectrum of

3-hydroxy-2'-methyl-Δ$^{1,3,5(10)}$-estratrieno[17,16-d]pyrimidine (3a)

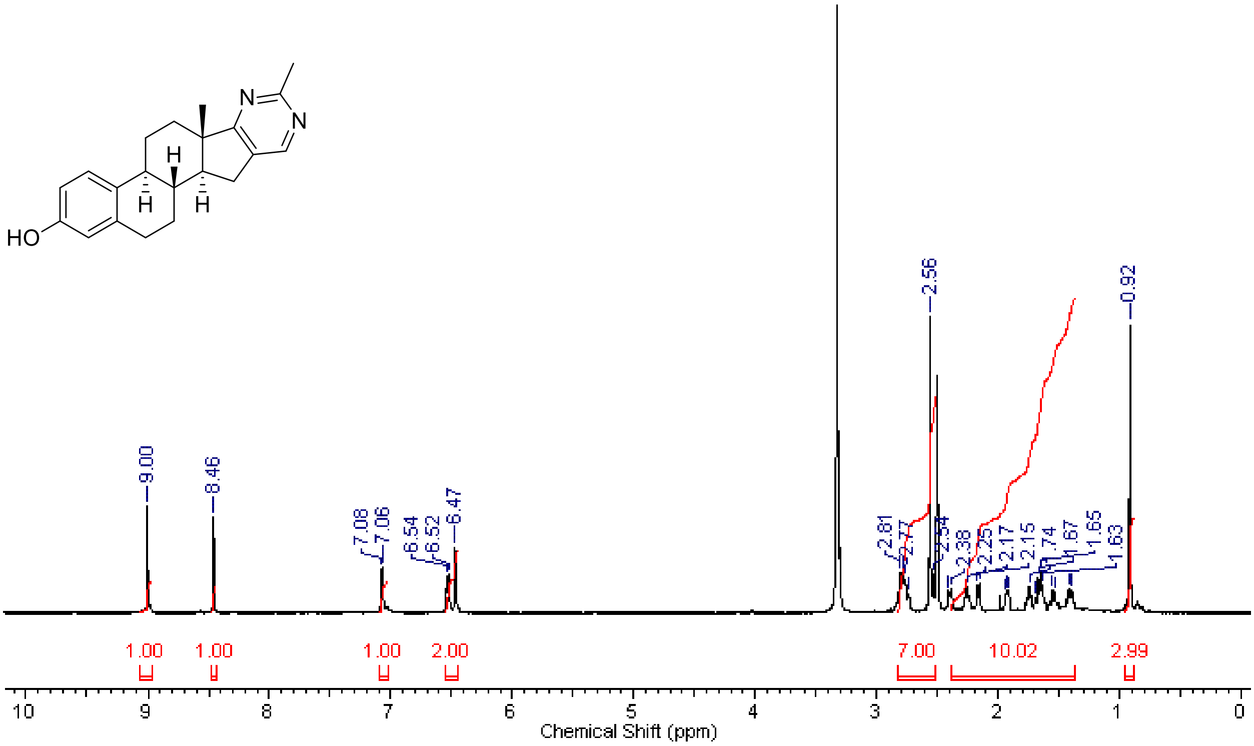

^{13}C NMR (DMSO-d6, 150 MHz) spectrum of

3-hydroxy-2'-methyl-Δ$^{1,3,5(10)}$-estratrieno[17,16-d]pyrimidine (3a)

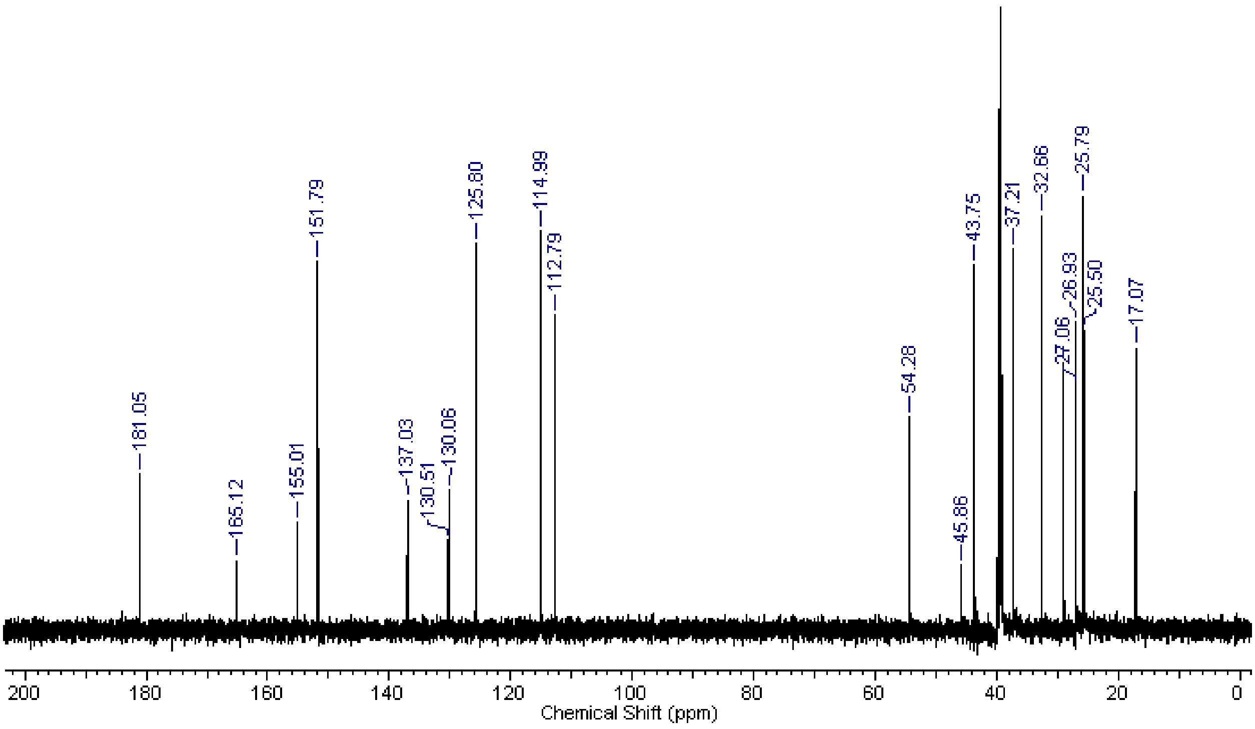

^{1}H-^{13}C HSQC NMR (DMSO-d_6) spectrum of

3-hydroxy-2'-methyl-Δ$^{1,3,5(10)}$-estratrieno[17,16-d]pyrimidine (3a)

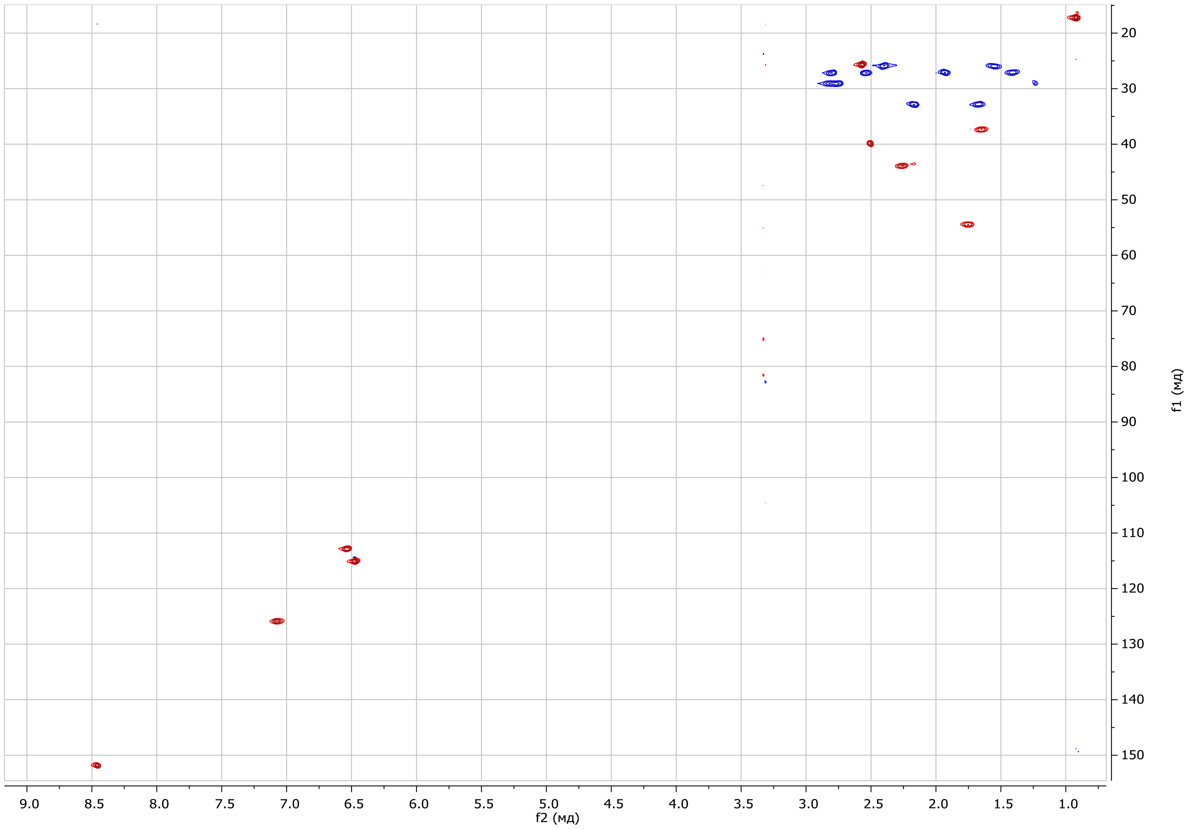

^{1}H-^{13}C HMBC NMR (DMSO-d_6) spectrum of

3-hydroxy-2'-methyl-Δ$^{1,3,5(10)}$-estratrieno[17,16-d]pyrimidine (3a)

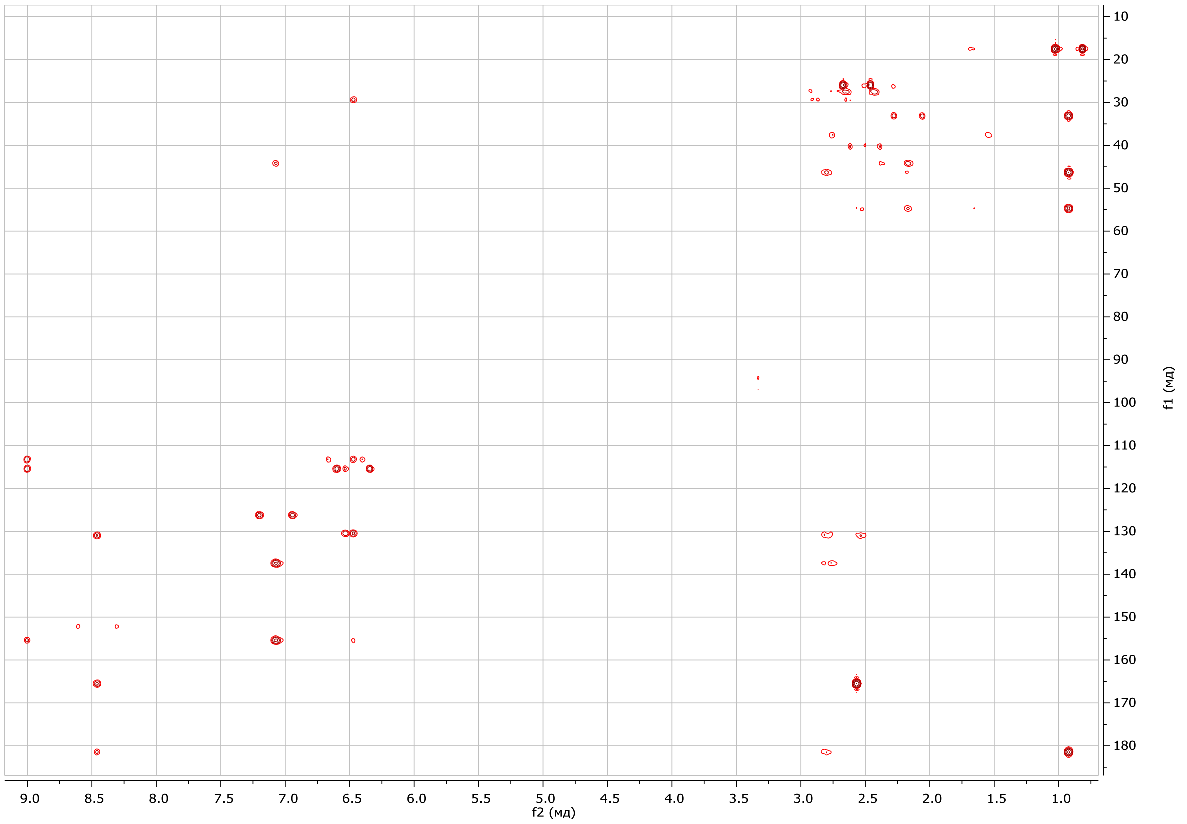

^{1}H-^{1}H COSY NMR (DMSO-d_6) spectrum of

3-hydroxy-2'-methyl-Δ$^{1,3,5(10)}$-estratrieno[17,16-d]pyrimidine (3a)

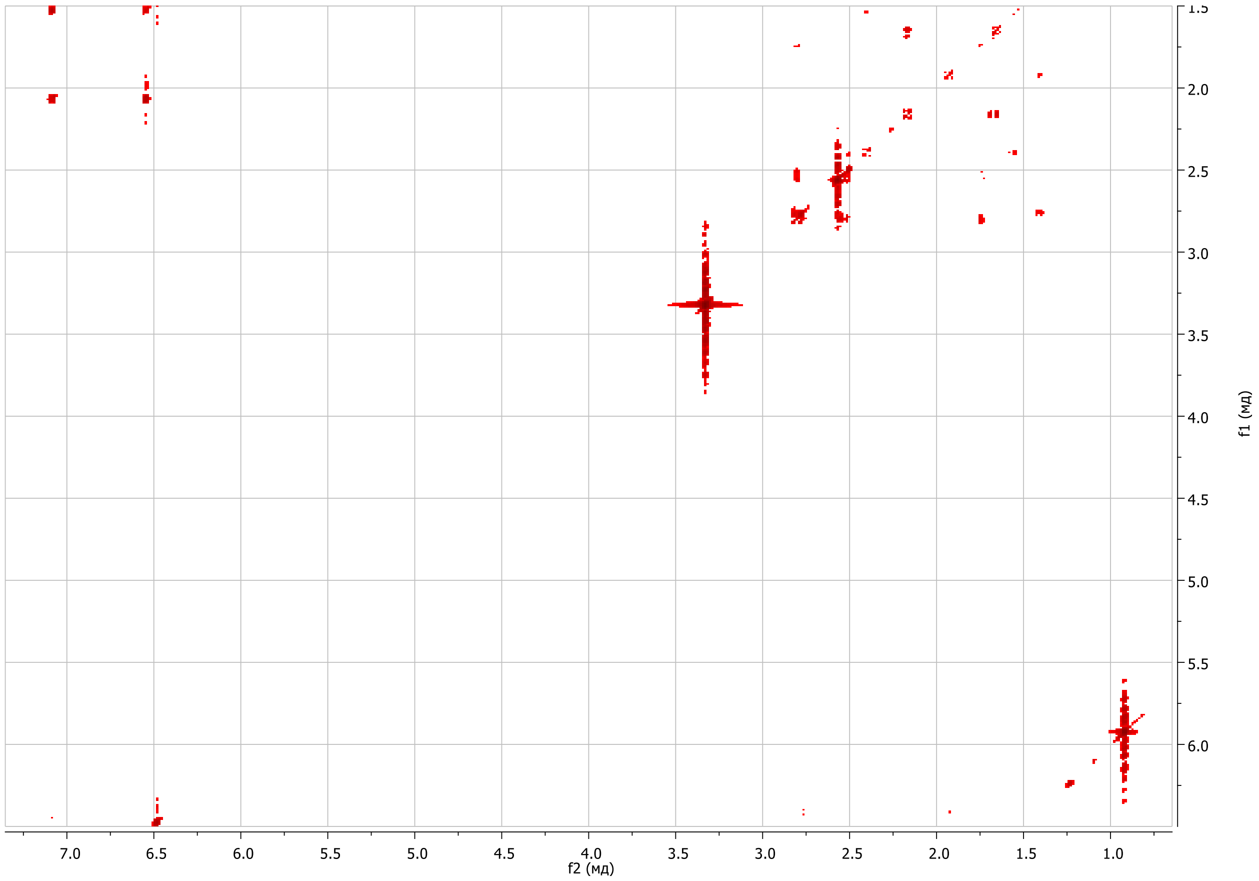

^1H NMR (DMSO-d_6, 600 MHz) spectrum of

2'-amino-3-hydroxy-Δ1,3,5(10)-estratrieno[17,16-d]pyrimidine (3b)

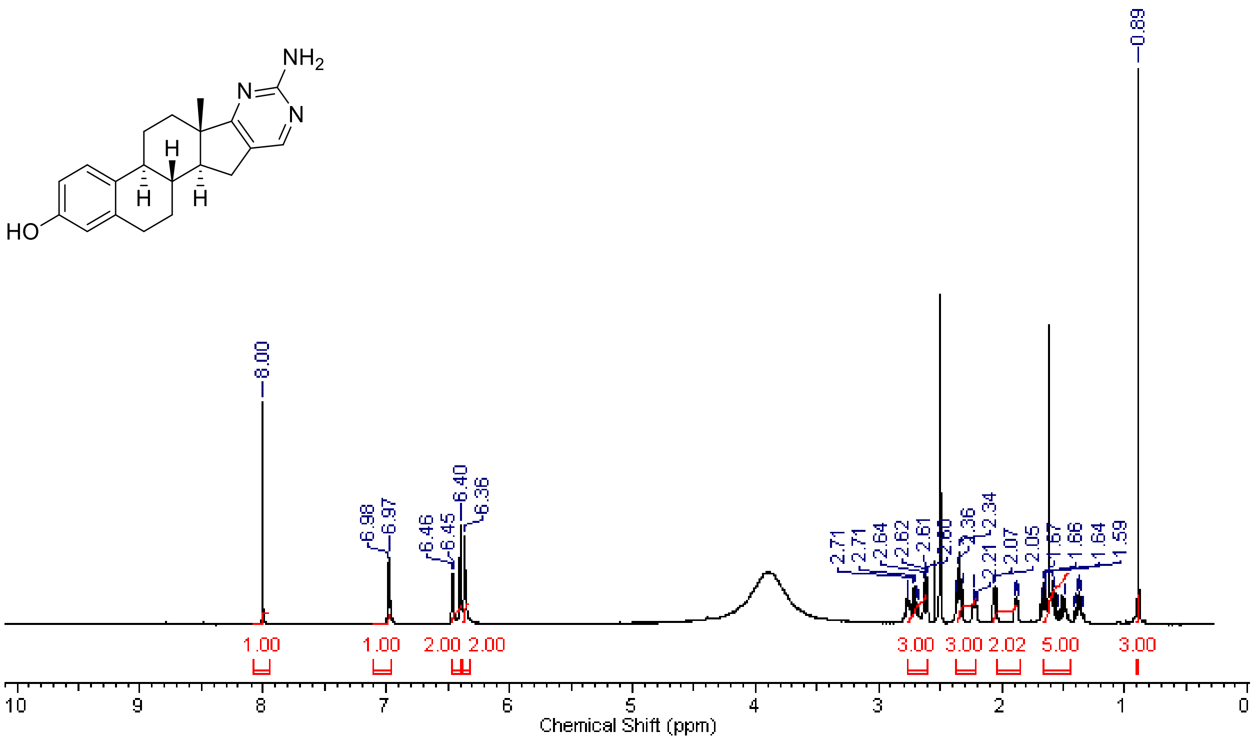

^{13}C NMR (DMSO-d_6, 150 MHz) spectrum of

2'-amino-3-hydroxy-Δ1,3,5(10)-estratrieno[17,16-d]pyrimidine (3b)

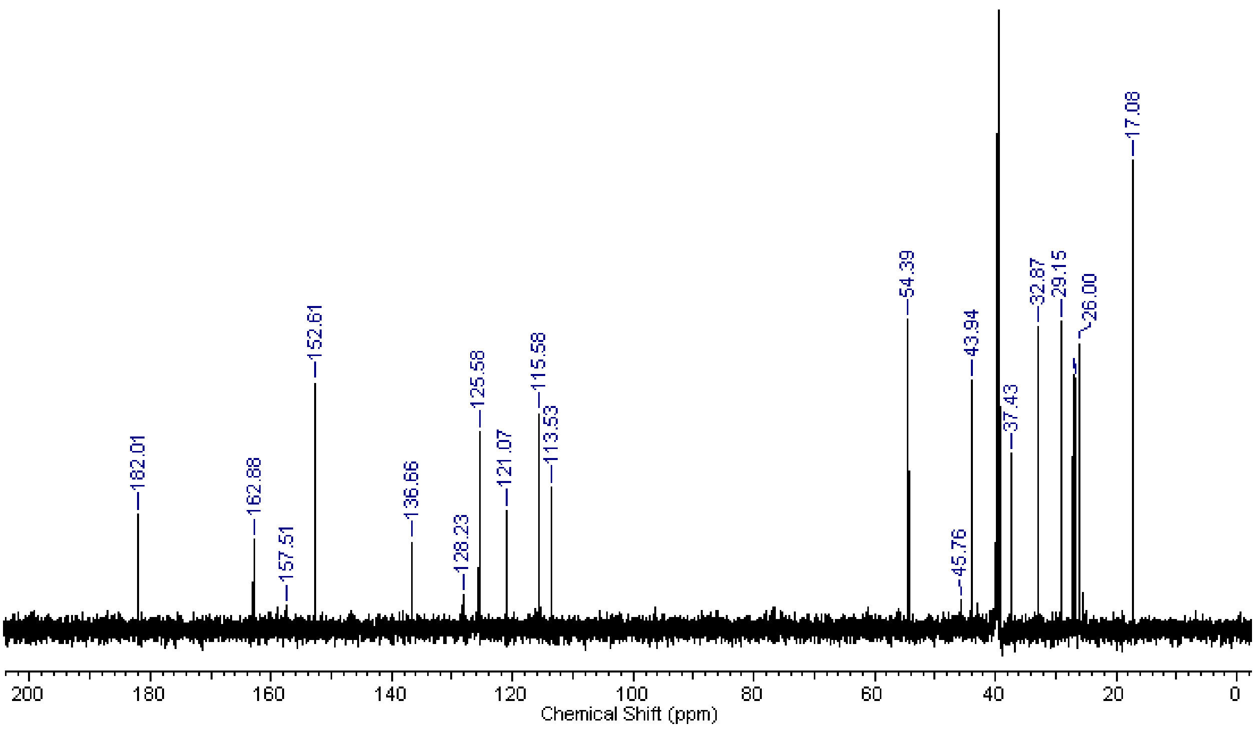

^1H-^{13}C HSQC NMR (DMSO-d_6, 600 MHz) spectrum of
2’-amino-3-hydroxy-Δ1,3,5(10)-estratrieno[17,16-d]pyrimidine (3b)

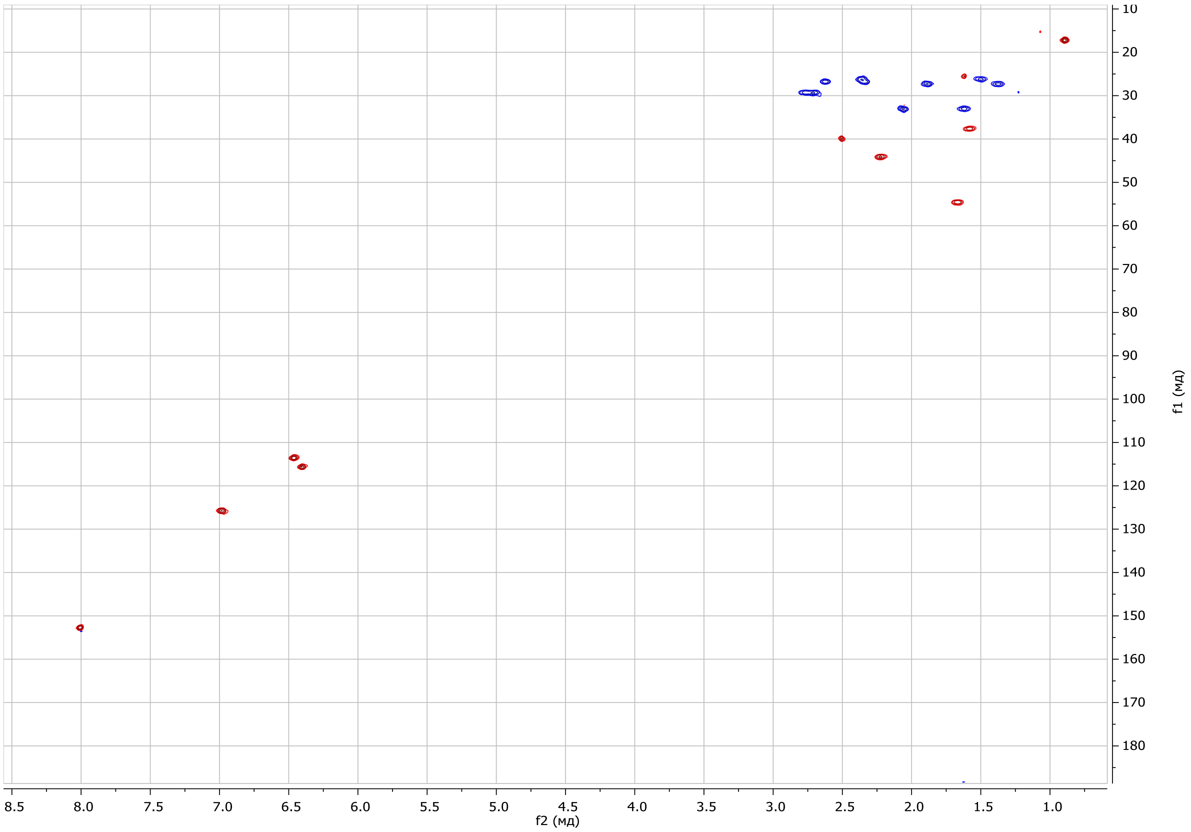

^1H-^{13}C HMBC NMR (DMSO-d_6, 600 MHz) spectrum of
2’-amino-3-hydroxy-Δ1,3,5(10)-estratrieno[17,16-d]pyrimidine (3b)

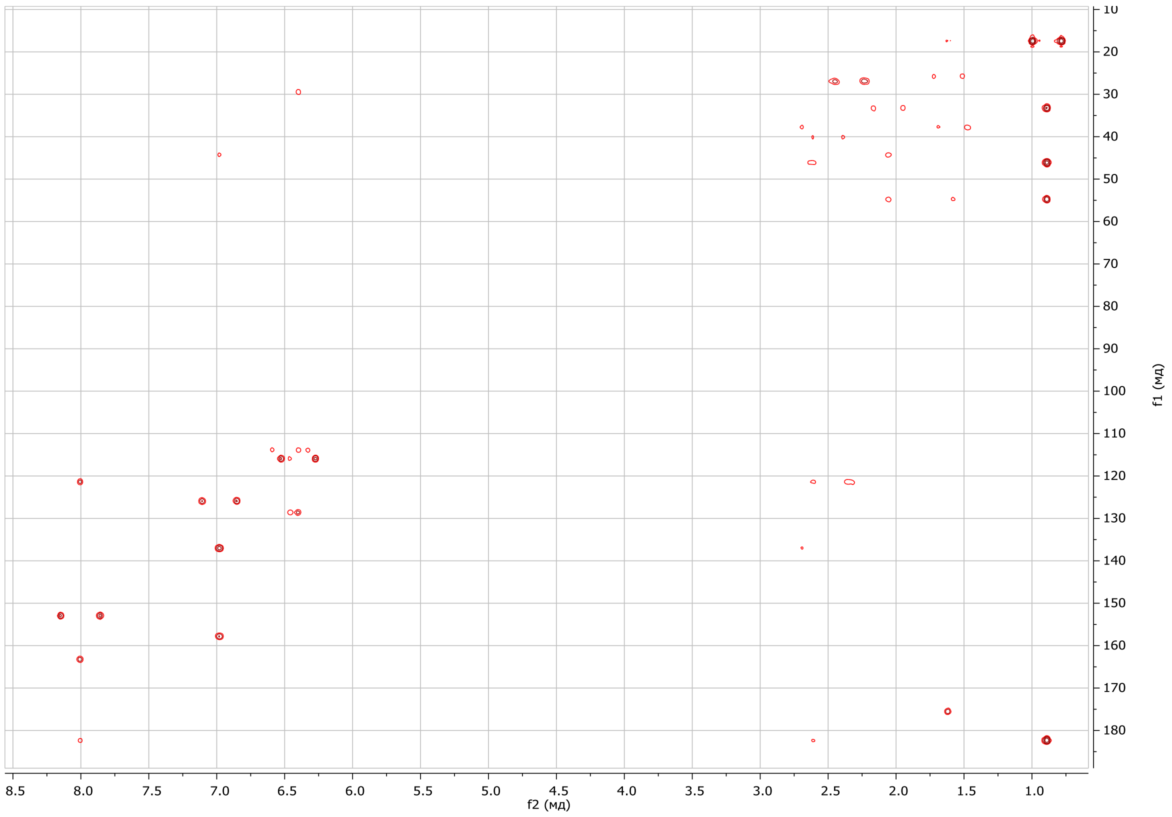

^{1}H-^{1}H COSY NMR (DMSO-d_6, 600 MHz) spectrum of

2’-amino-3-hydroxy-Δ1,3,5(10)-estratrieno[17,16-d]pyrimidine (3b)

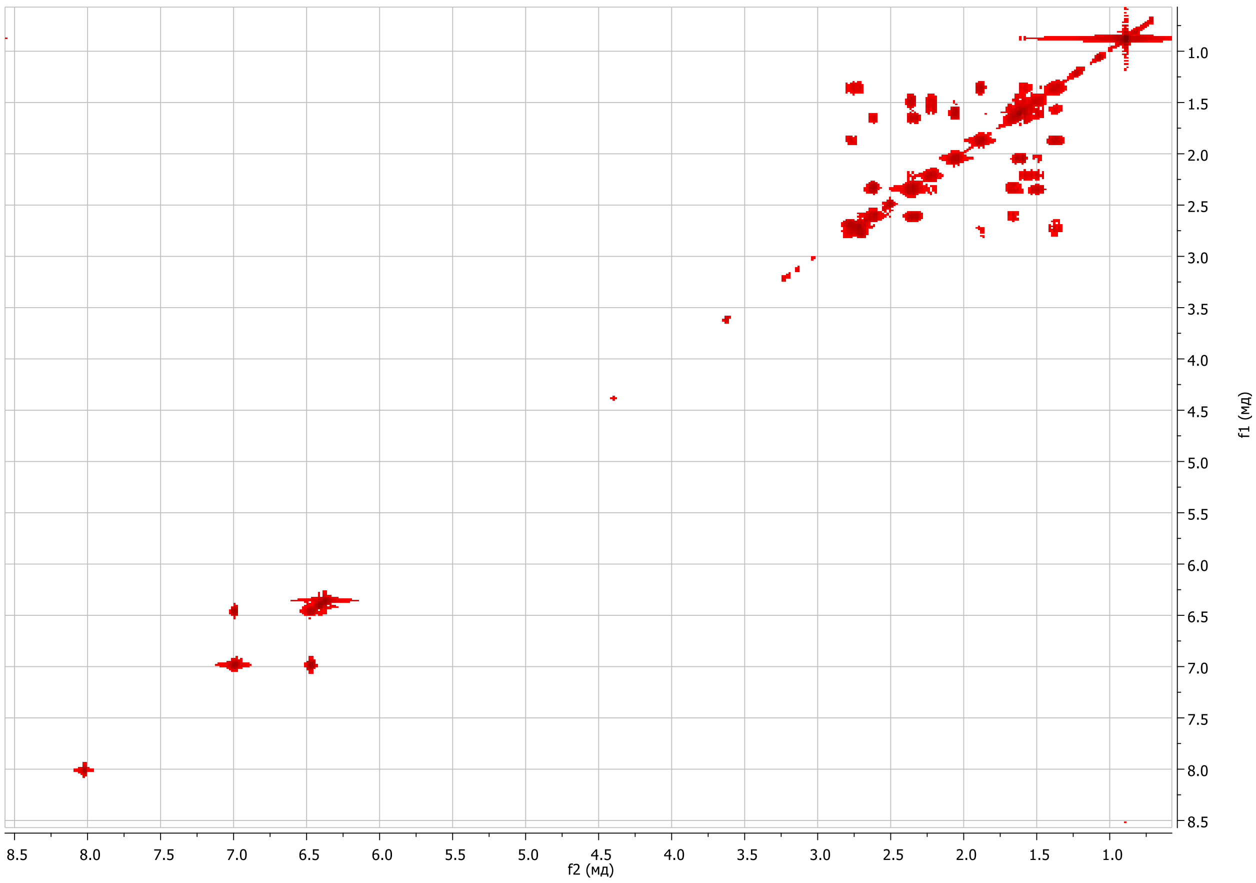

^{1}H NMR (DMSO-d_6, 600 MHz at 303K) spectrum of

3β-acetoxy-3'-methyl-5-androsteno[17,16-d]pyrimidine (3c)

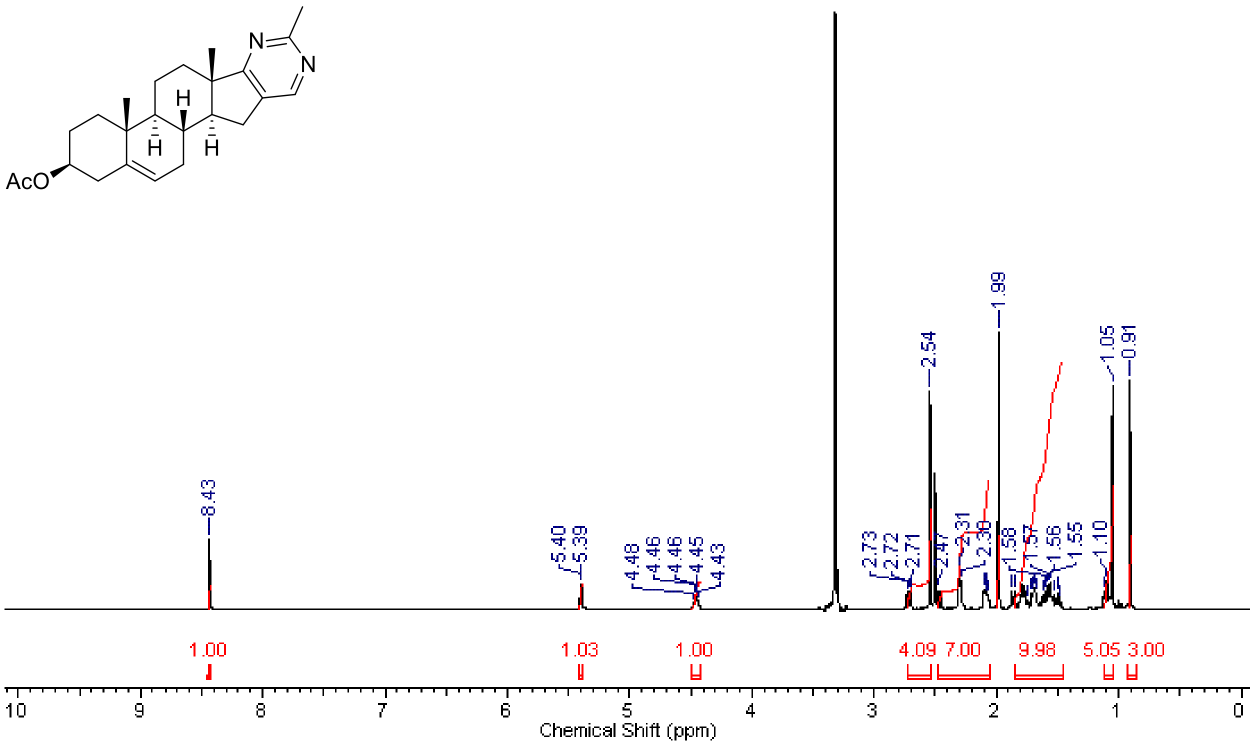

^{13}C NMR (DMSO-d_6, 150 MHz at 303K) spectrum of

3β-acetoxy-3'-methyl-5-androsteno[17,16-d]pyrimidine (3c)

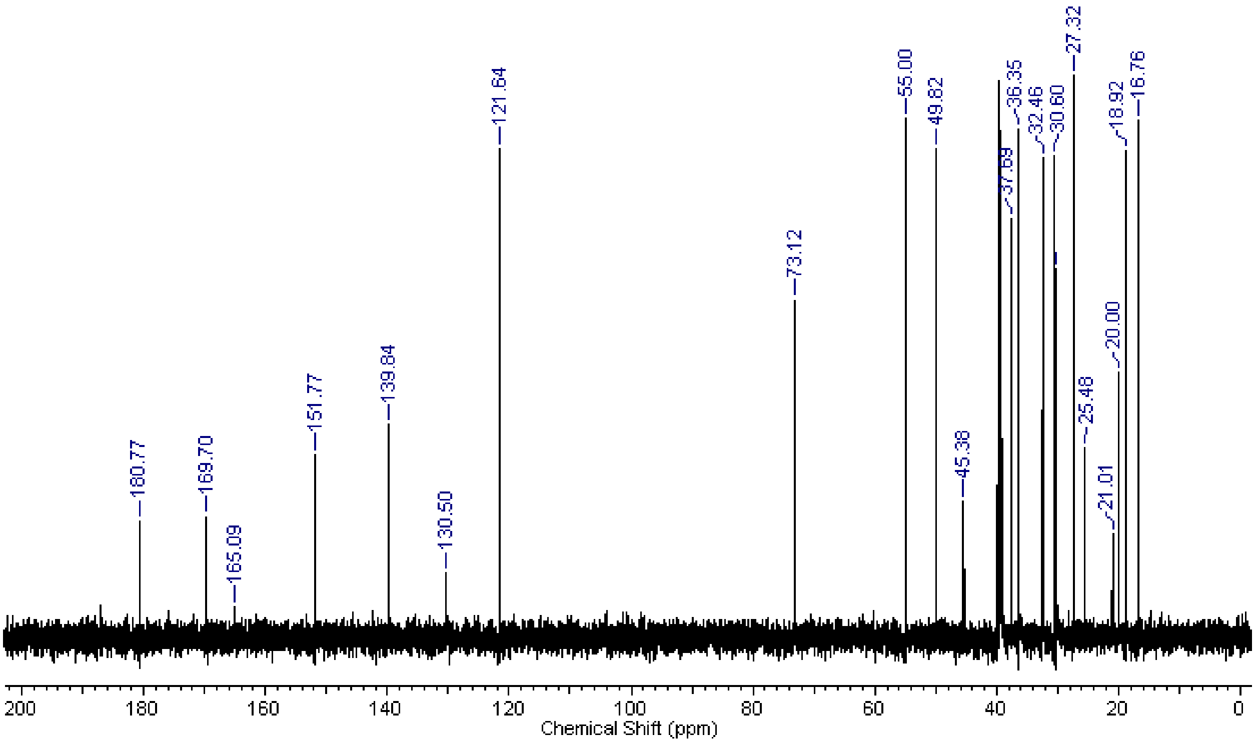

^{1}H-^{13}C HSQC NMR (DMSO-d_6 at 303K) spectrum of

3β-acetoxy-3’-methyl-5-androsteno[17,16-d]pyrimidine (3c)

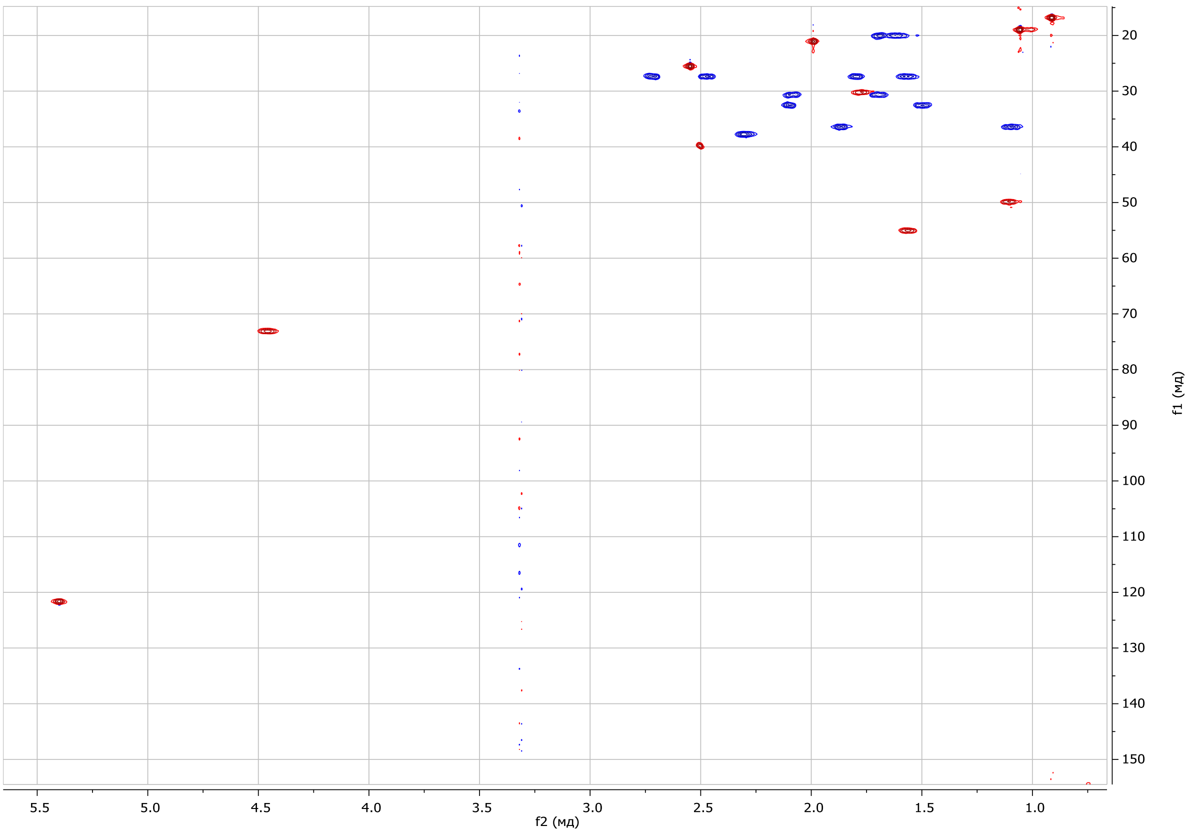

^{1}H-^{13}C HMBC NMR (DMSO-d_6 at 303K) spectrum of

3β-acetoxy-3’-methyl-5-androsteno[17,16-d]pyrimidine (3c)

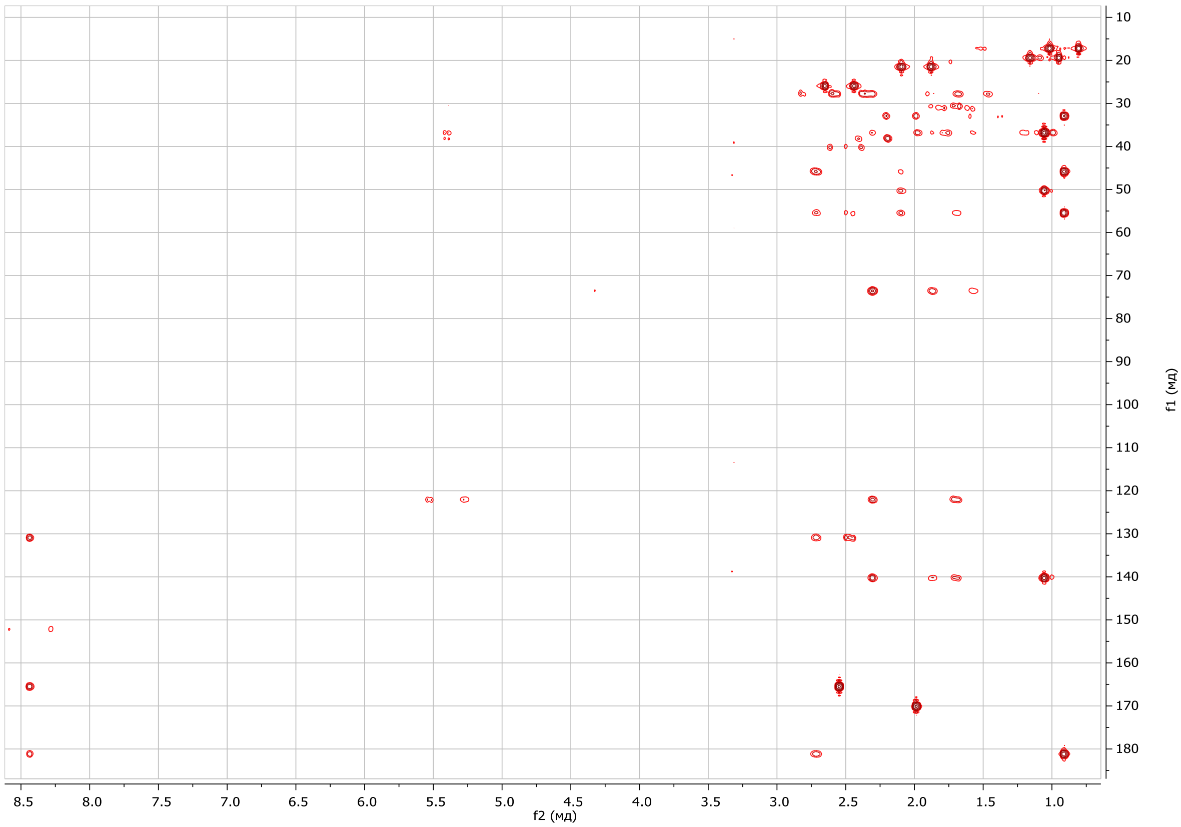

^{1}H-^{1}H COSY NMR (DMSO-d6 at 303K) spectrum of

3β-acetoxy-3’-methyl-5-androsteno[17,16-d]pyrimidine (3c)

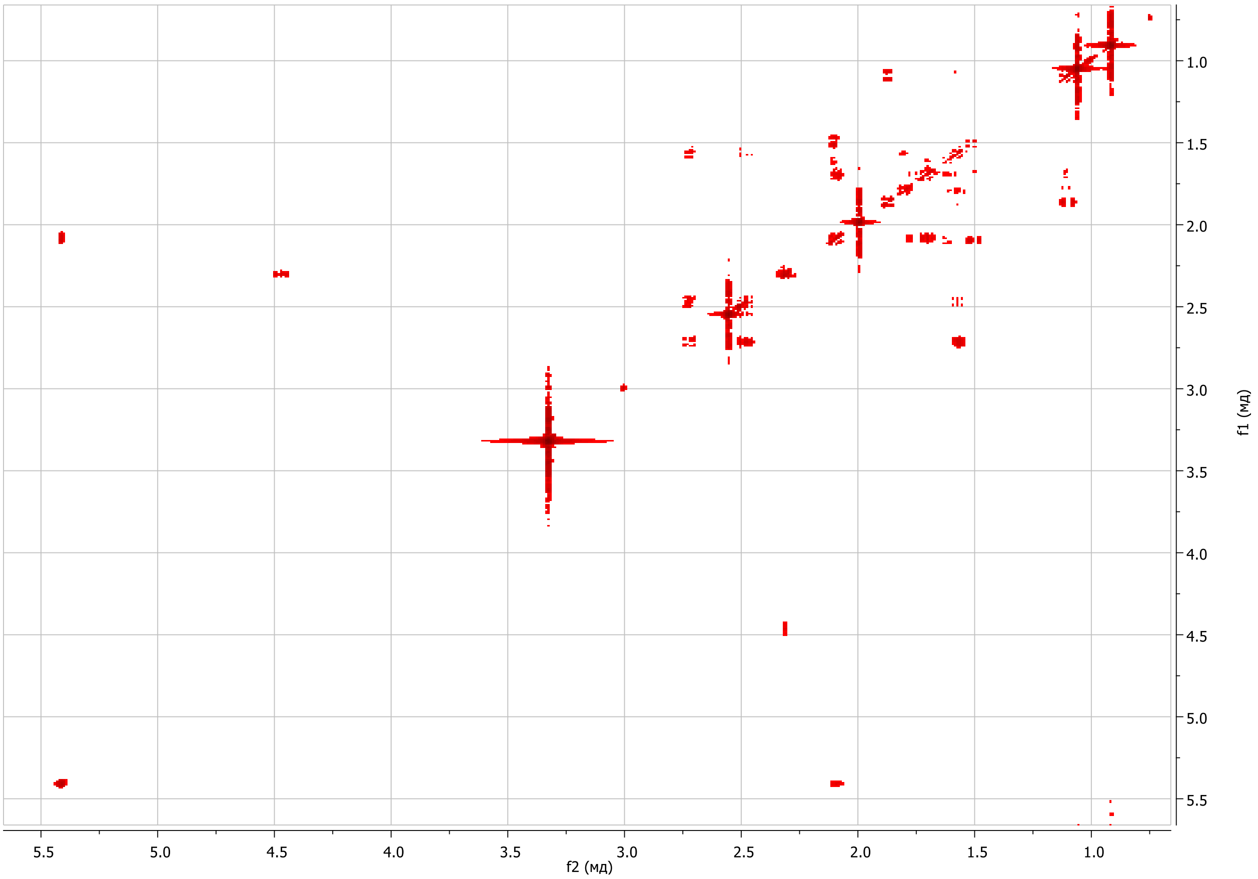

^{1}H NMR (DMSO-d_6+ CCl_4, 600 MHz) spectrum of

3β-Hydroxy-3’-methyl-5-androsteno[17,16-d]pyrimidine (3d)

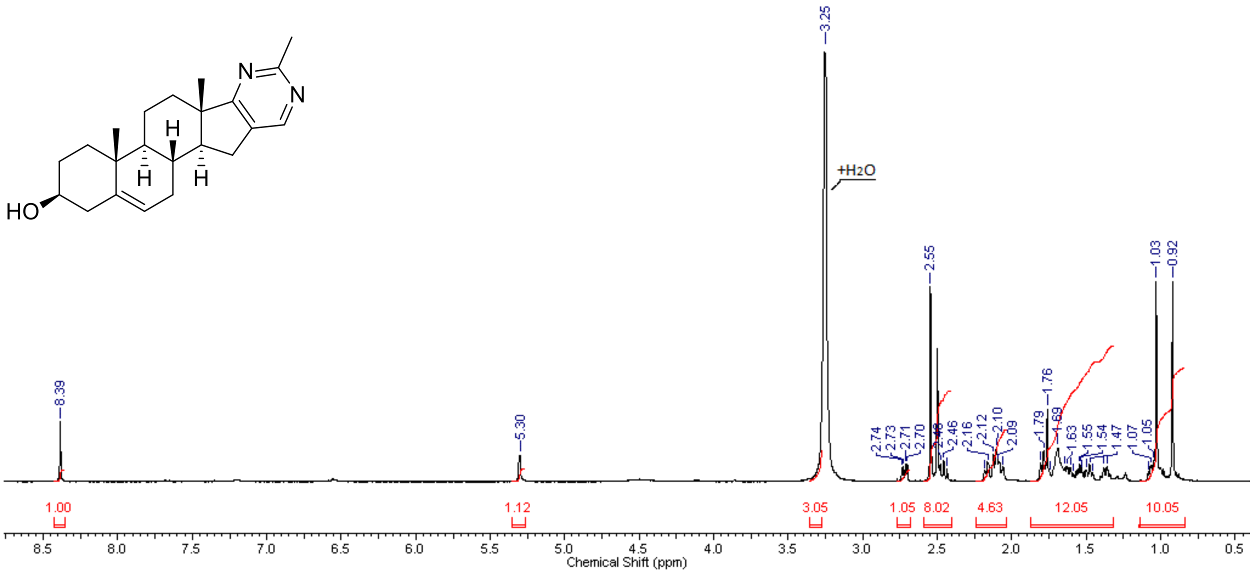

^{13}C NMR (DMSO-d_6 + CCl_4, 150 MHz) spectrum of

3β-Hydroxy-3’-methyl-5-androsteno[17,16-d]pyrimidine (3d)

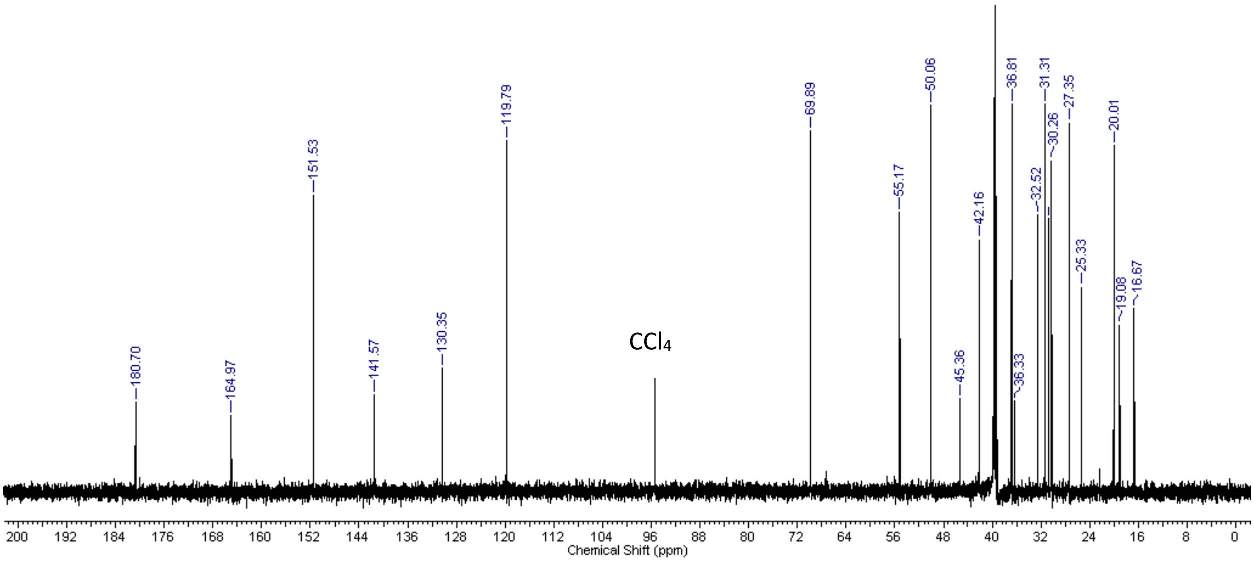

^{1}H-^{13}C HSQC NMR (DMSO-d$_6$ + CCl_4, 600 MHz) spectrum of

3β-Hydroxy-3’-methyl-5-androsteno[17,16-d]pyrimidine (3d)

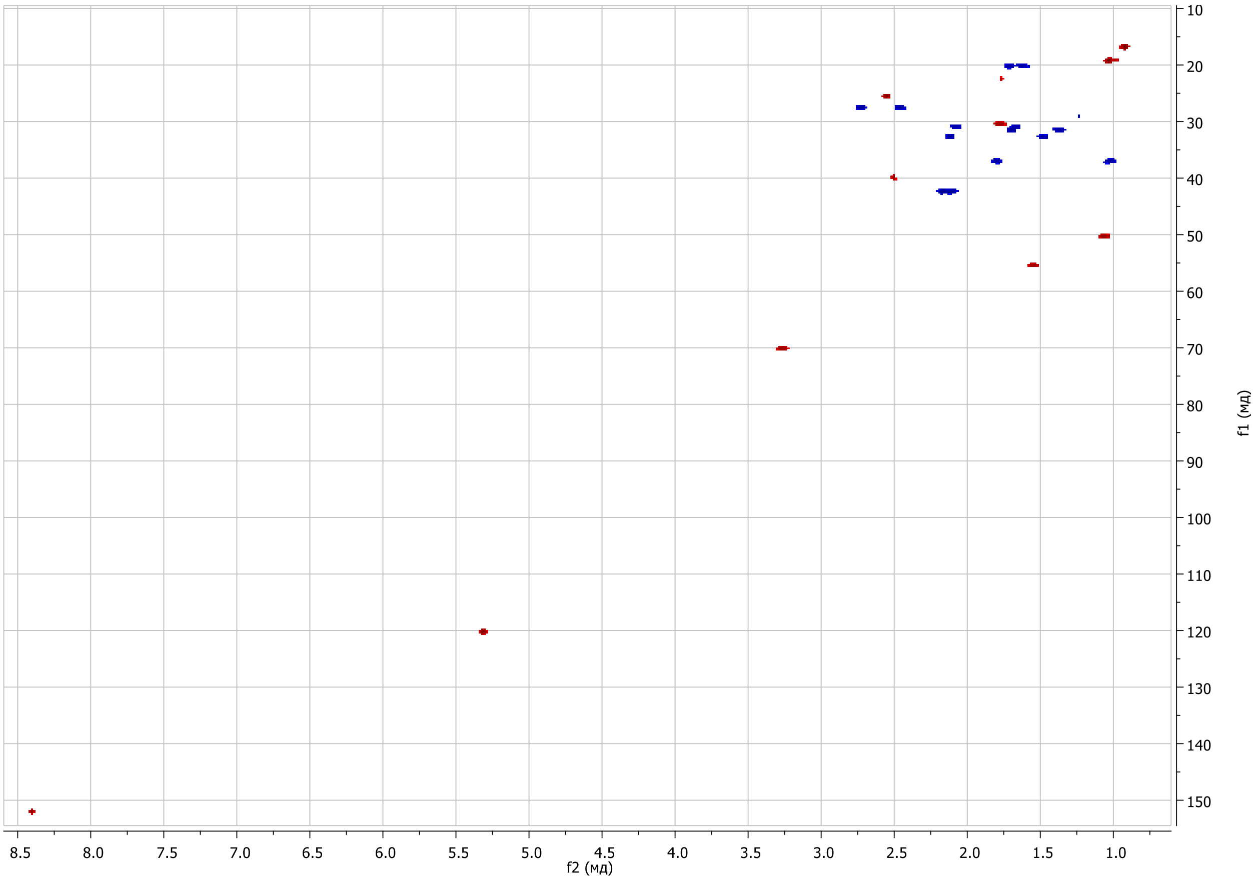

^{1}H-^{13}C HMBC NMR (DMSO-d$_6$ + CCl_4, 600 MHz) spectrum of

3β-Hydroxy-3’-methyl-5-androsteno[17,16-d]pyrimidine (3d)

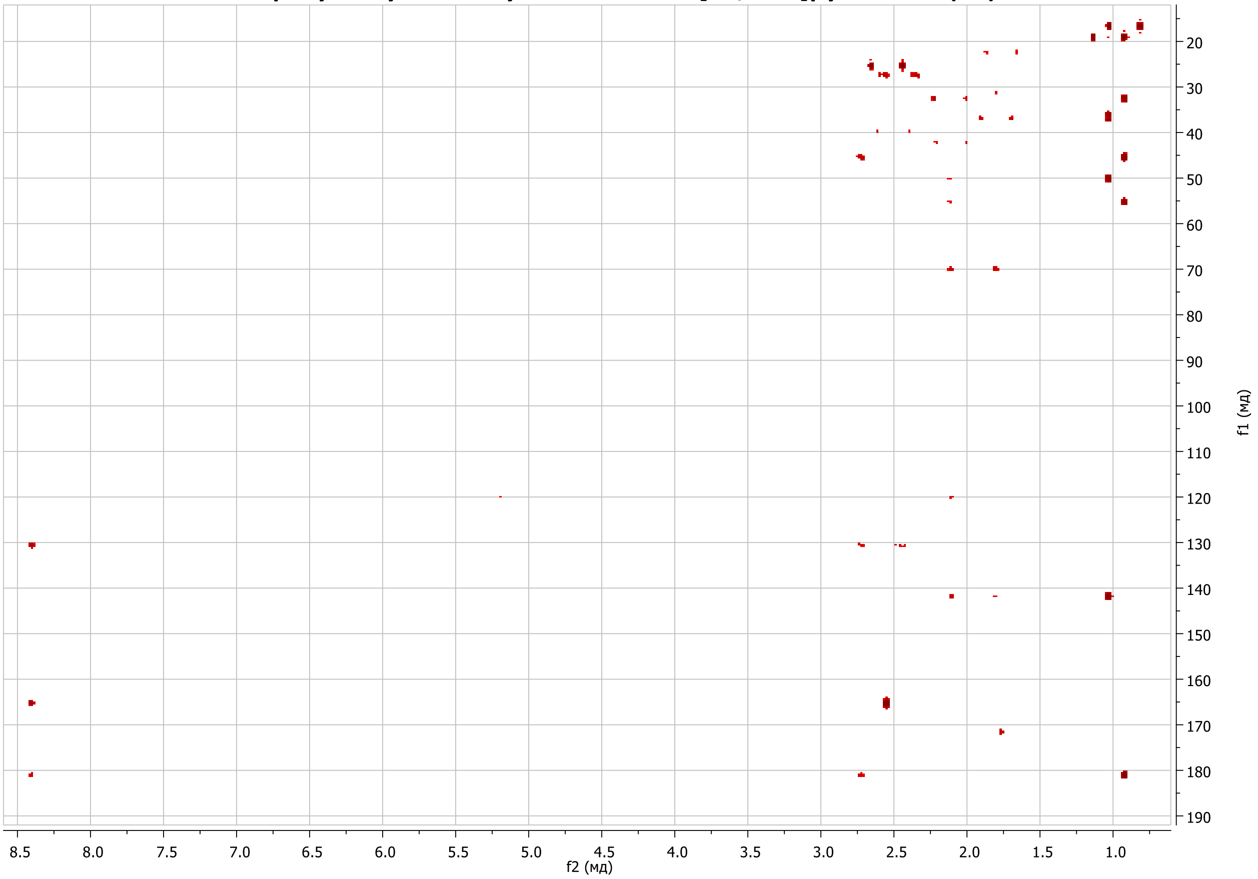

^1H-^1H COSY NMR (DMSO-d$_6$ + CCl_4) spectrum of

3β-Hydroxy-3’-methyl-5-androsteno[17,16-d]pyrimidine (3d)

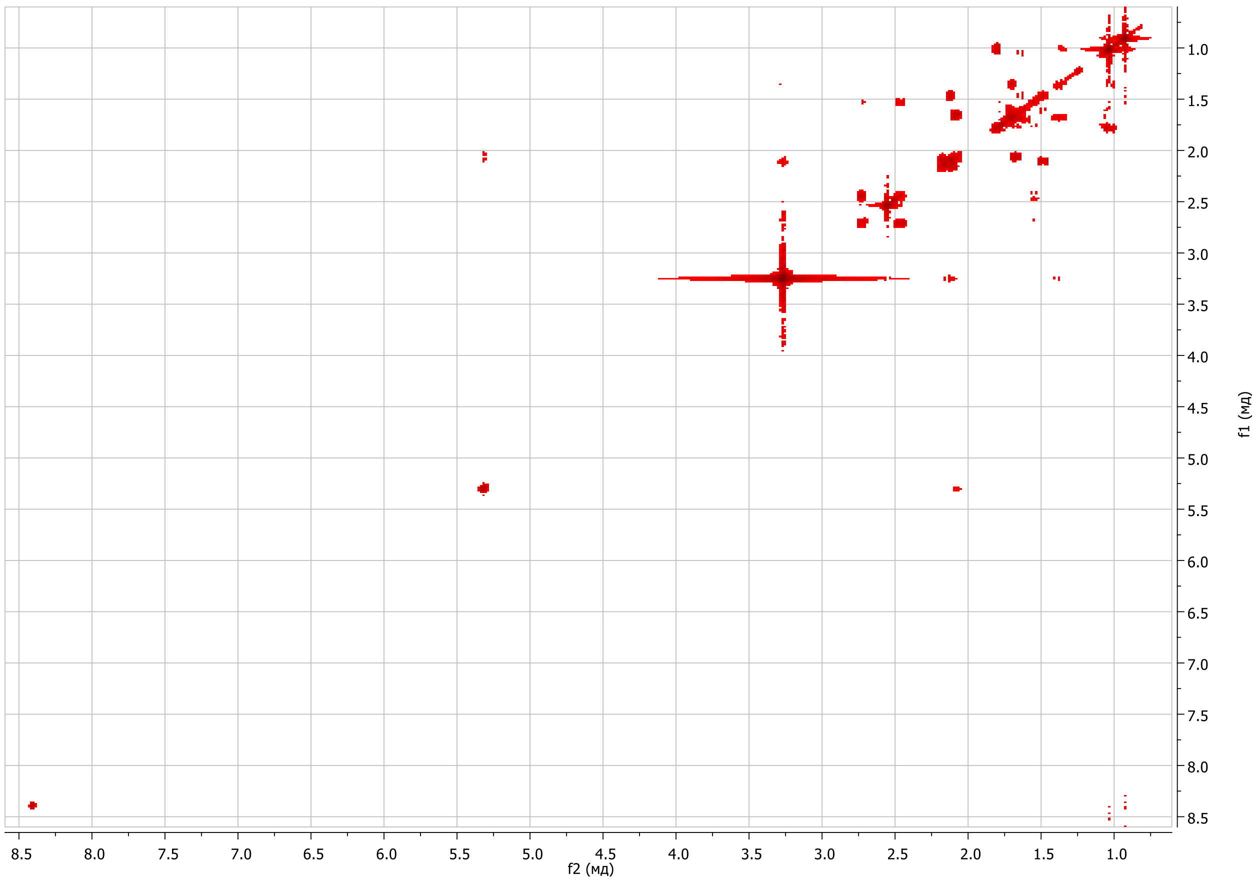

^{1}H NMR (DMSO-d$_6$, 600 MHz at 333K) spectrum of

2’-amino-3β-hydroxy-5-androsteno[17,16-d]pirimydine (3e)

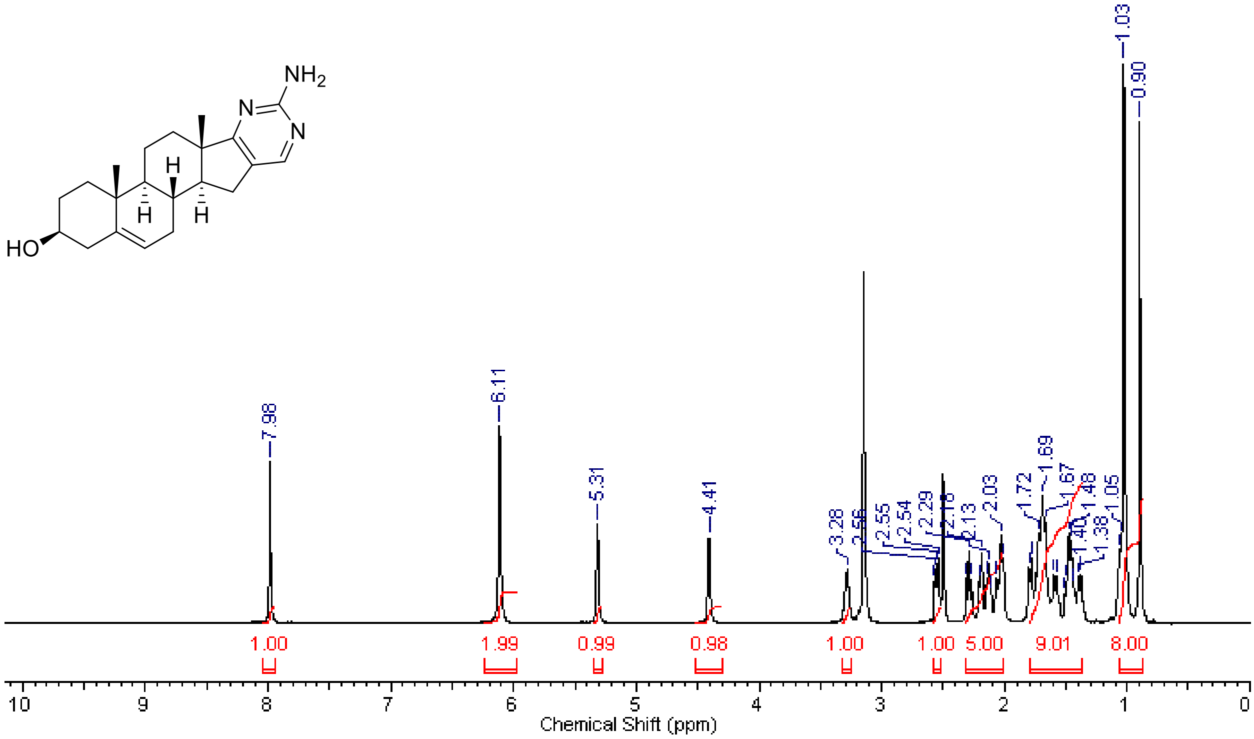

^{13}C NMR (DMSO-d$_6$, 150 MHz at 333K) spectrum of

2’-amino-3β-hydroxy-5-androsteno[17,16-d]pirimydine (3e)

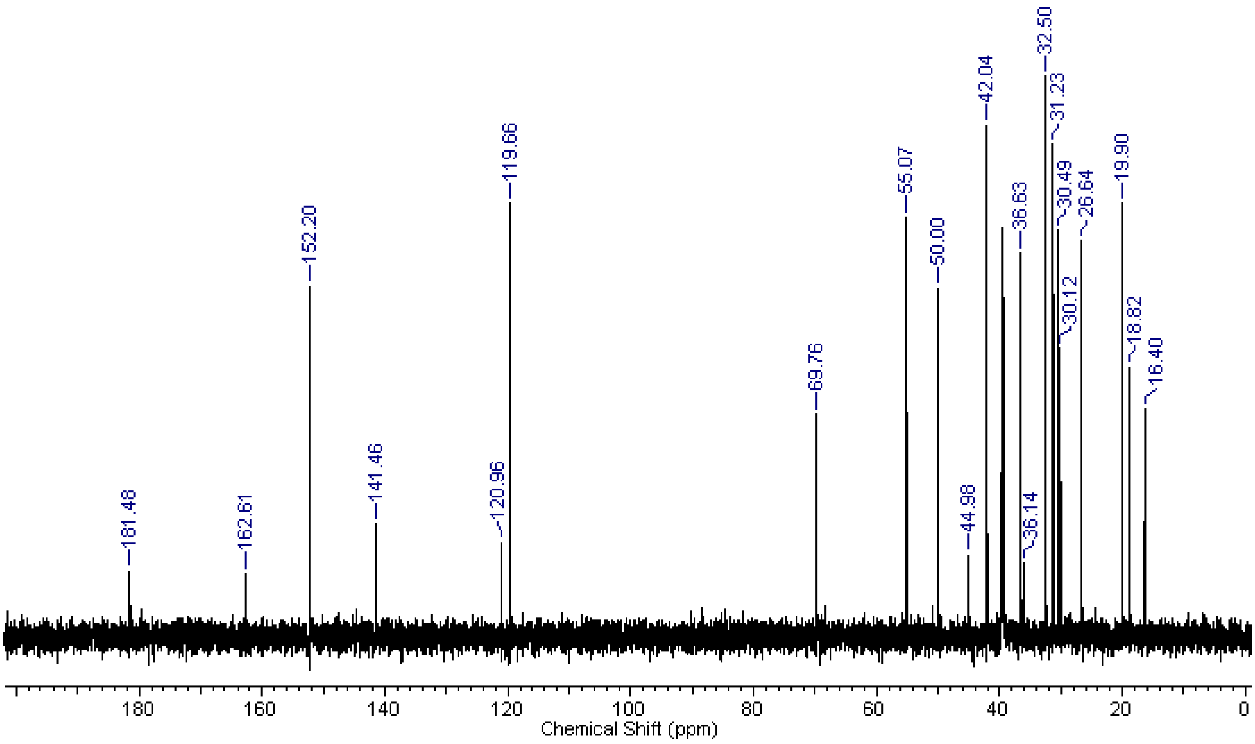

^{1}H-^{13}C HSQC NMR (DMSO-d$_{6}$ at 333K, 600 MHz) spectrum of

2’-amino-3β-hydroxy-5-androsteno[17,16-d]pirimydine (3e)

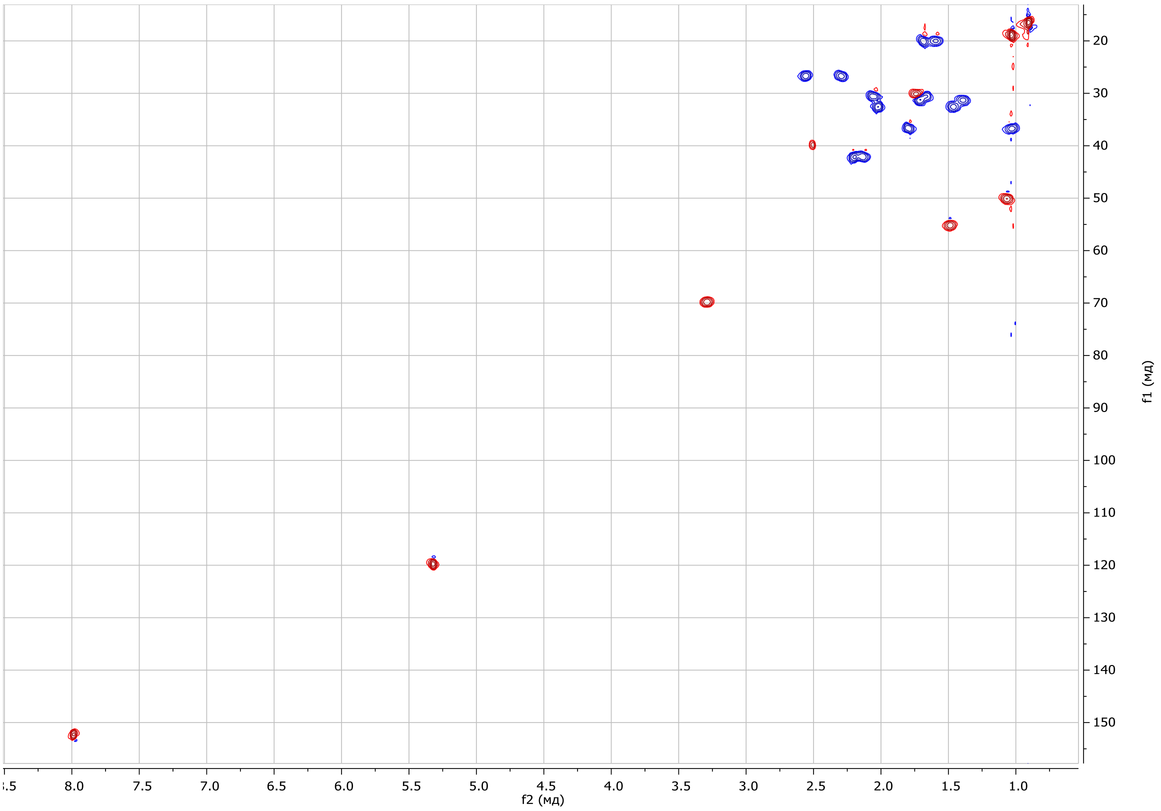

^{1}H-^{13}C HMBC NMR (DMSO-d$_{6}$ at 333K, 600 MHz) spectrum of

2’-amino-3β-hydroxy-5-androsteno[17,16-d]pirimydine (3e)

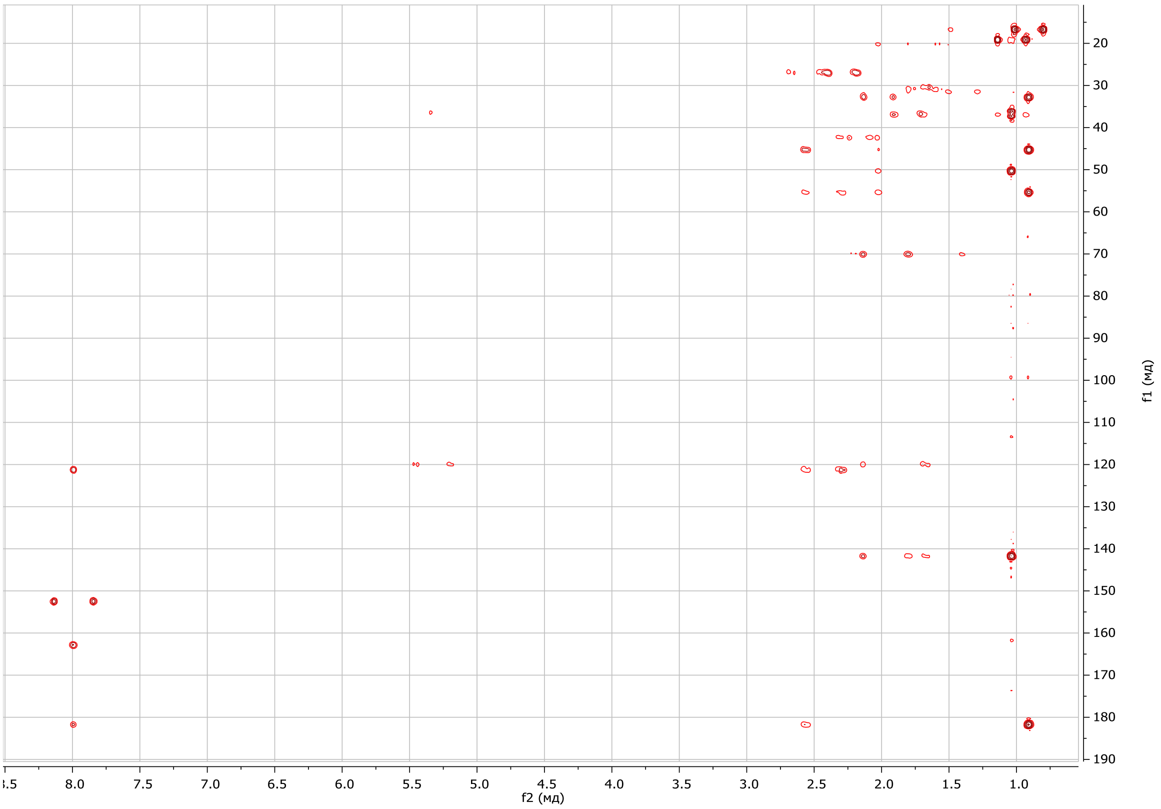

^{1}H NMR (DMSO-d$_6$, 600 MHz) spectrum of **2’-amino-17β-hydroxy-5α-androstano[2,3-d]pyrimidine (3f)**

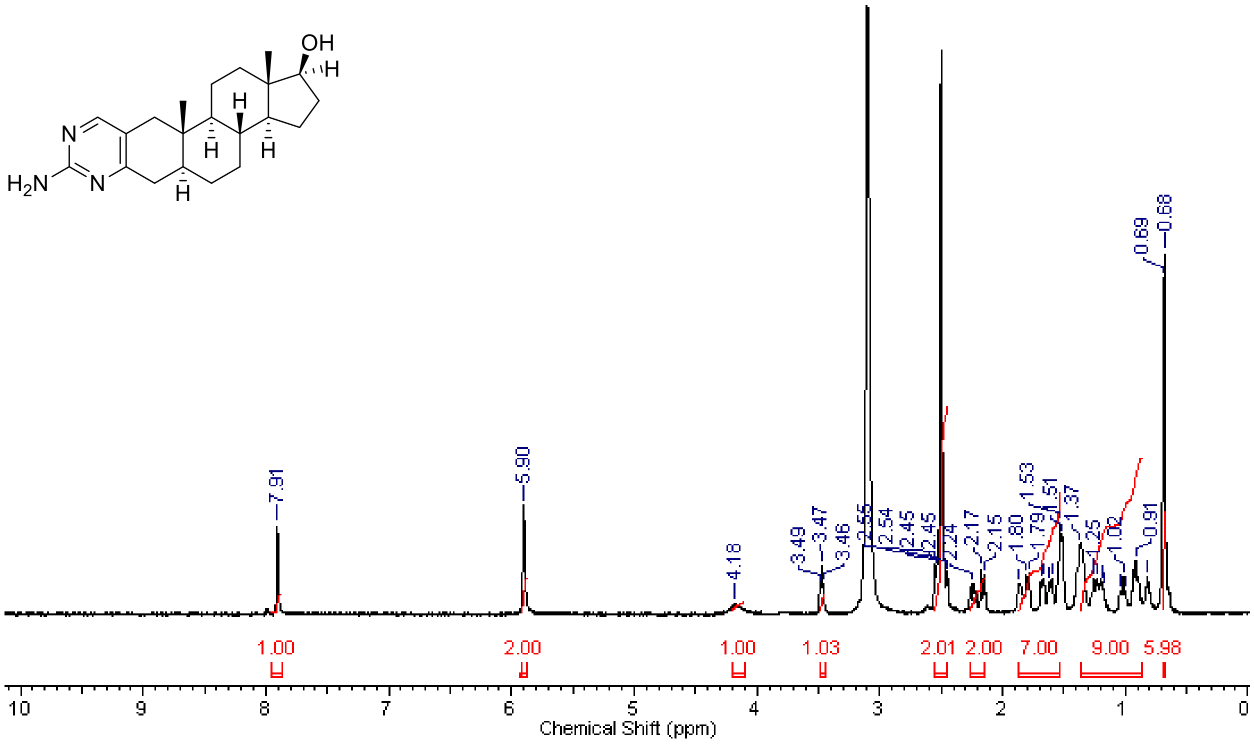

^{13}C NMR (DMSO-d$_6$, 150 MHz) spectrum of **2’-amino-17β-hydroxy-5α-androstano[2,3-d]pyrimidine (3f)**

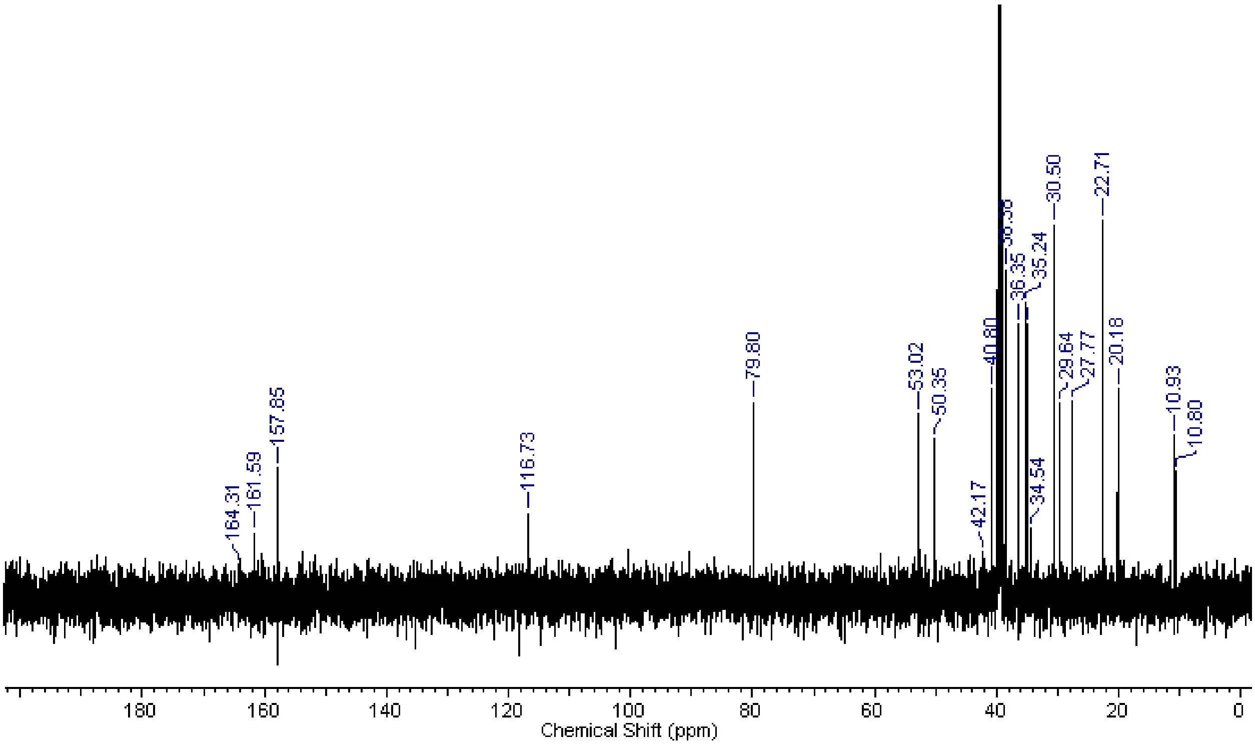

^{1}H-^{13}C HSQC NMR (DMSO-d_6, 600 MHz) spectrum of
2’-amino-17β-hydroxy-5α-androstano[2,3-d]pyrimidine (3f)

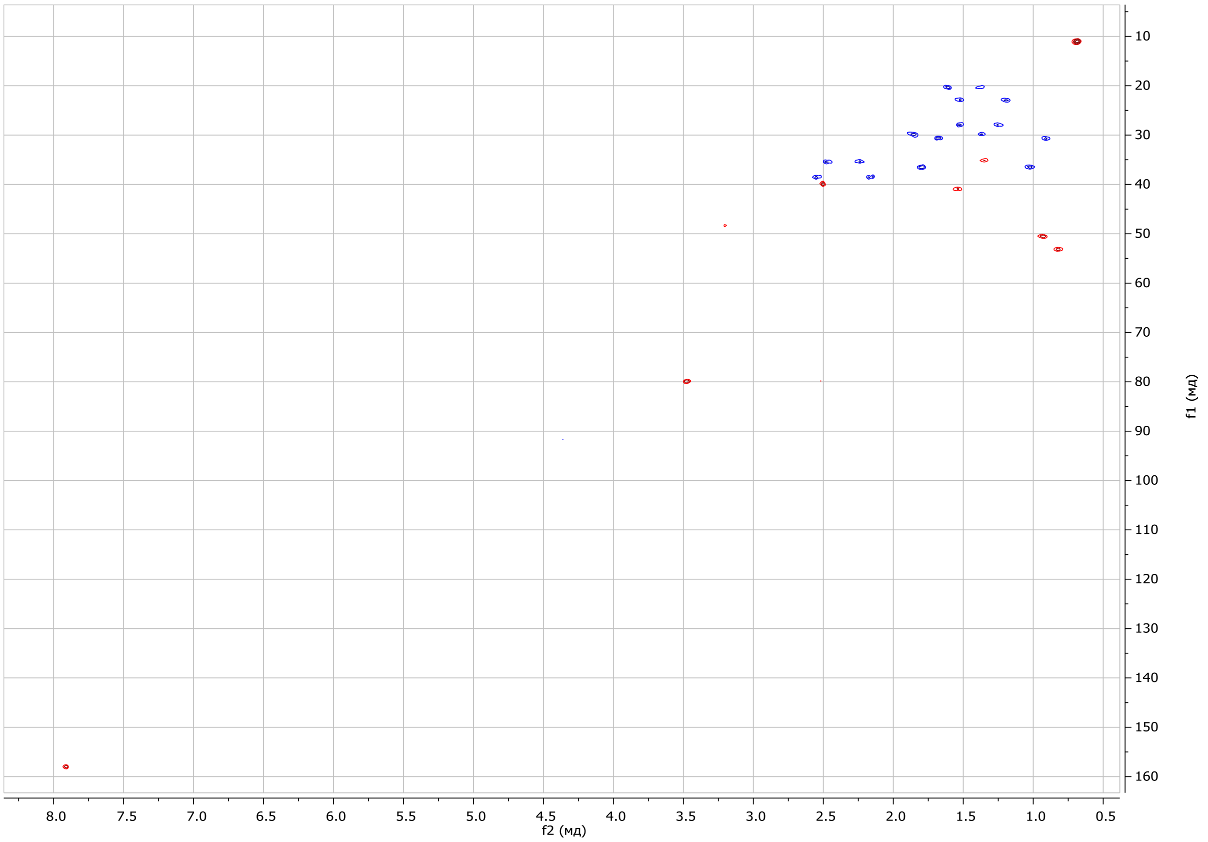

^{1}H-^{13}C HMBC NMR (DMSO-d_6, 600 MHz) spectrum of
2’-amino-17β-hydroxy-5α-androstano[2,3-d]pyrimidine (3f)

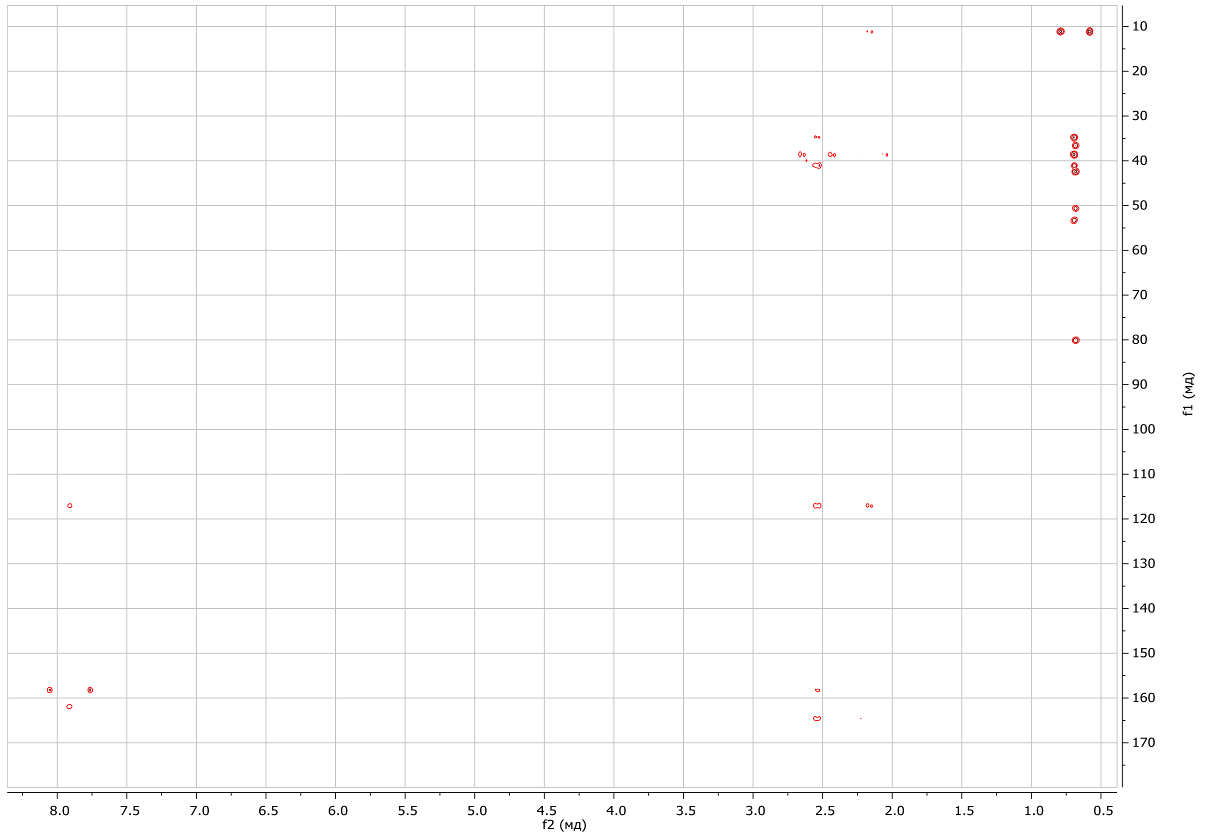

^1H-^1H COSY NMR (DMSO-d_6, 600 MHz) spectrum of

2’-amino-17β-hydroxy-5α-androstano[2,3-d]pyrimidine (3f)

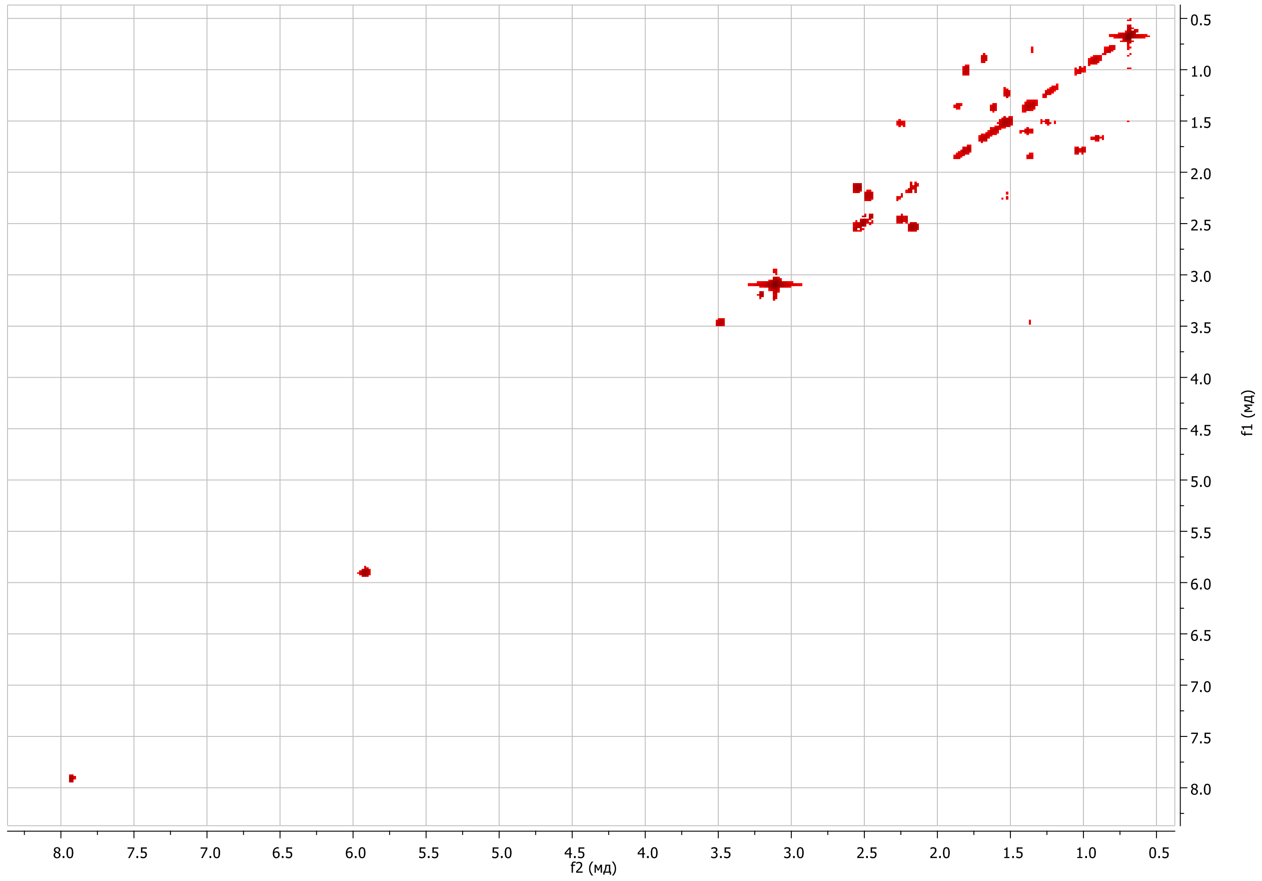

^{1}H NMR (DMSO-d_6, 600 MHz) spectrum of

16-(4, 6-dimethyl-1,2-dihydro-1,3,5-triazin-2-il)-17-chlor-$\Delta^{1,3,5(10),16}$-estratetraen-3-ol (4a)

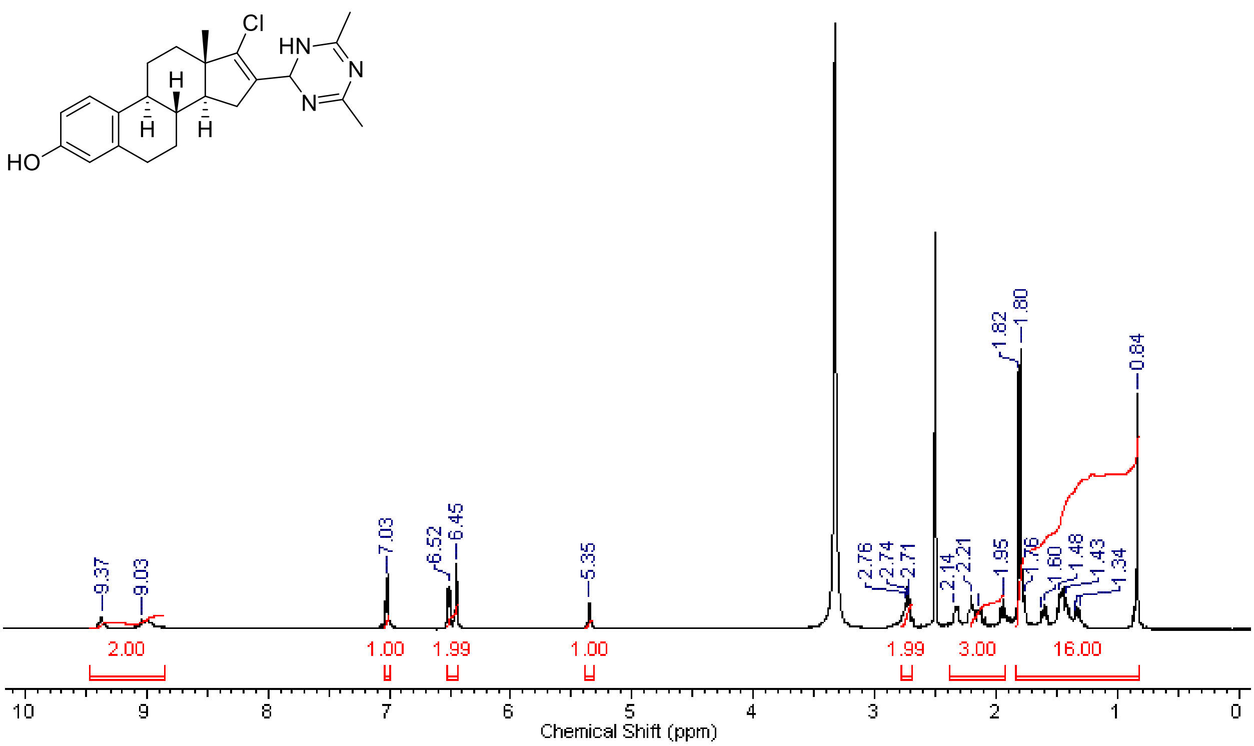

^{13}C NMR (DMSO-d_6, 150 MHz) spectrum of

16-(4, 6-dimethyl-1,2-dihydro-1,3,5-triazin-2-il)-17-chlor-$\Delta^{1,3,5(10),16}$-estratetraen-3-ol (4a)

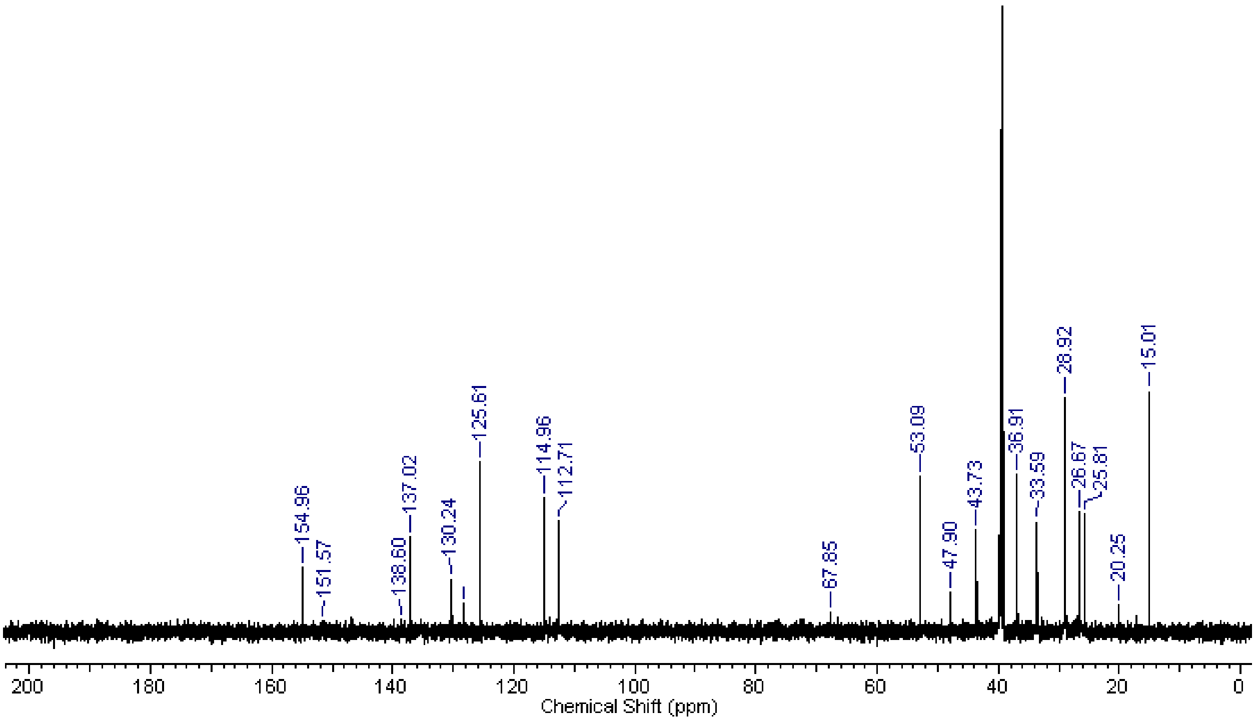

^{1}H-^{13}C HSQC NMR (DMSO-d_6) spectrum of

16-(4, 6-dimethyl-1,2-dihydro-1,3,5-triazin-2-il)-17-chlor-$\Delta^{1,3,5(10),16}$-estratetraen-3-ol (4a)

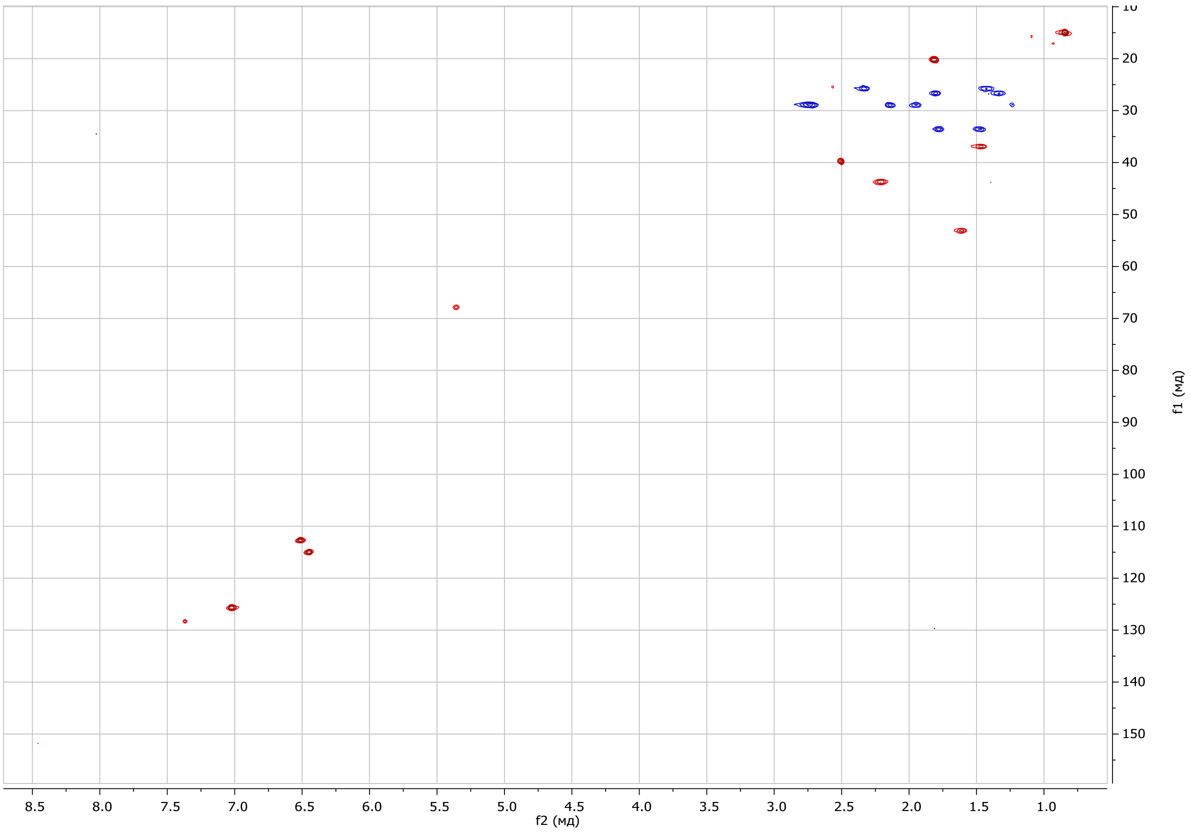

^{1}H-^{13}C HSQC NMR (DMSO-d_6) spectrum of

16-(4, 6-dimethyl-1,2-dihydro-1,3,5-triazin-2-il)-17-chlor-$\Delta^{1,3,5(10),16}$-estratetraen-3-ol (4a)

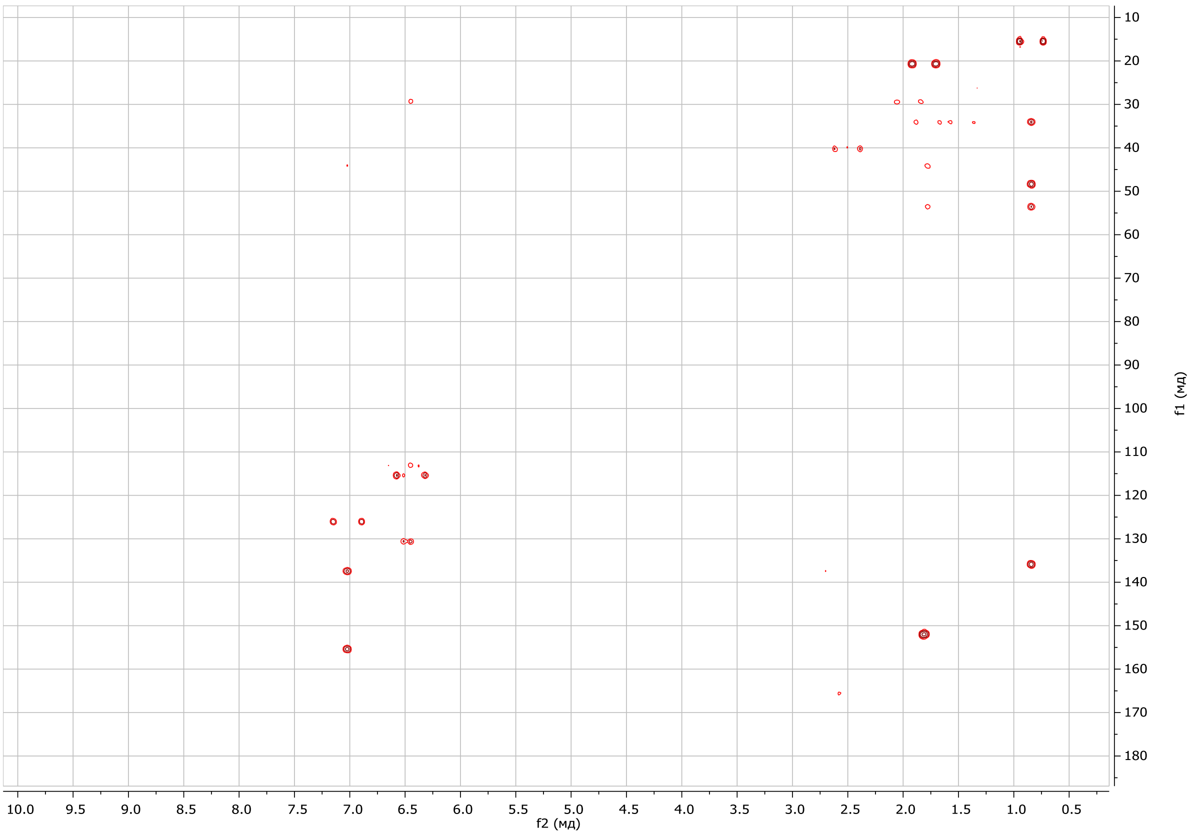

^{1}H-^{1}H COSY NMR (DMSO-d_6) spectrum of

16-(4, 6-dimethyl-1,2-dihydro-1,3,5-triazin-2-il)-17-chlor-$\Delta^{1,3,5(10),16}$-estratetraen-3-ol (4a)

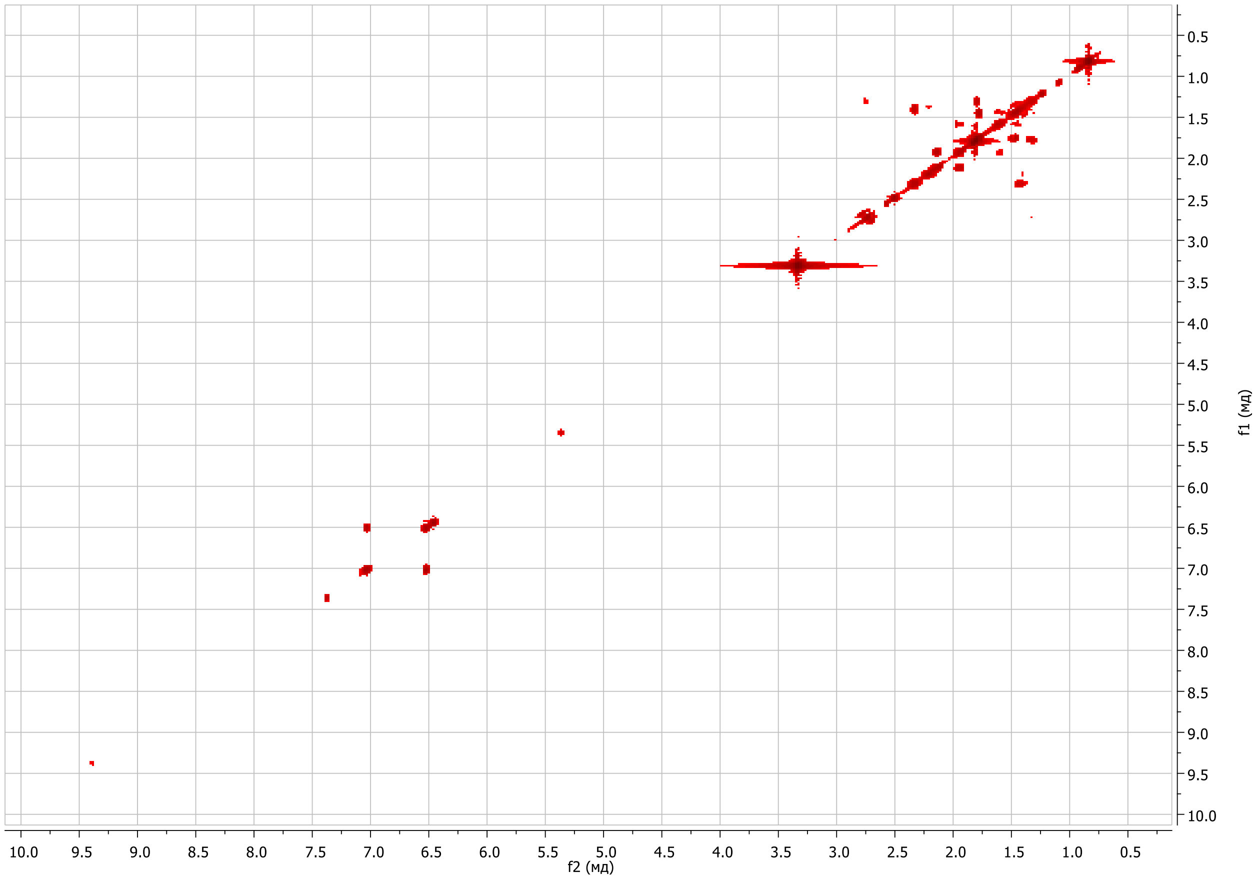

^{1}H NMR (DMSO-d$_6$, 600 MHz) spectrum of

3β-acetoxy-16-(4,6-dimethyl-1,2-dihydro-1,3,5-triazi-2-il)-17-chlorandrosta-5,16-dien (4b)

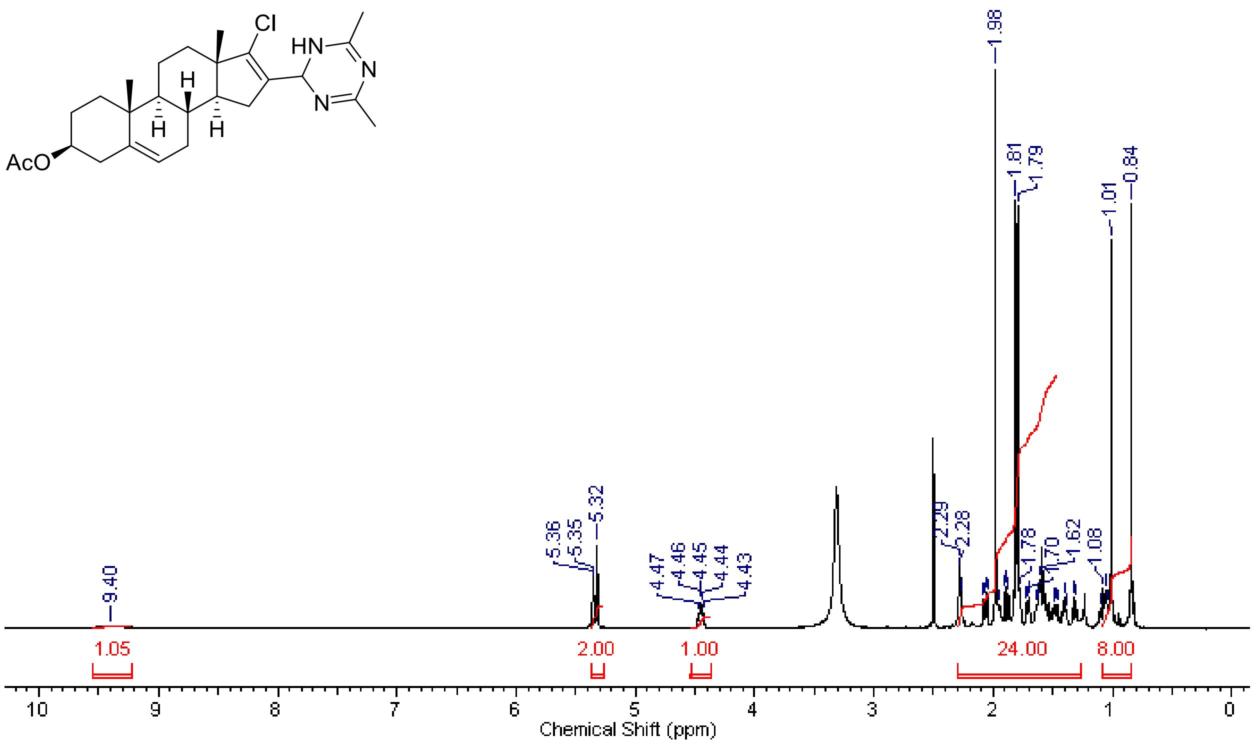

^{13}C NMR (DMSO-d$_6$, 150 MHz) spectrum of

3β-acetoxy-16-(4,6-dimethyl-1,2-dihydro-1,3,5-triazi-2-il)-17-chlorandrosta-5,16-dien (4b)

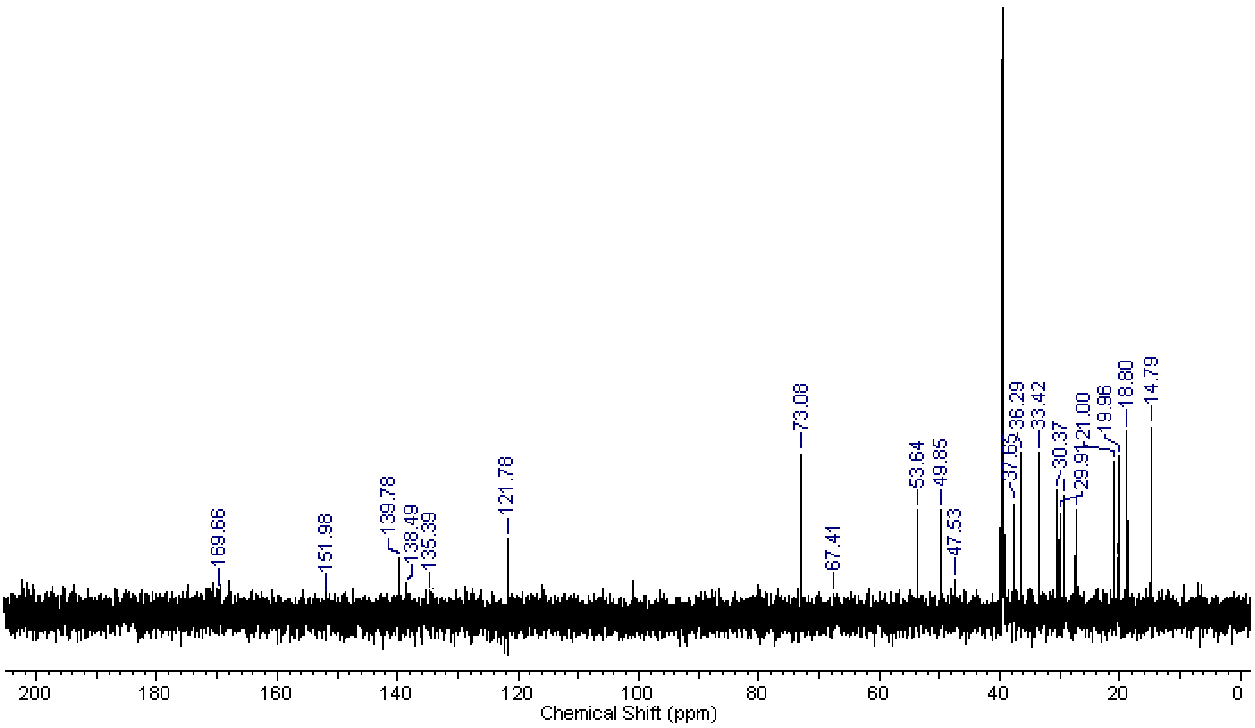

^{1}H-^{13}C HSQC NMR (DMSO-d_6) spectrum of

3β-acetoxy-16-(4,6-dimethyl-1,2-dihydro-1,3,5-triazi-2-il)-17-chlorandrosta-5,16-dien (4b)

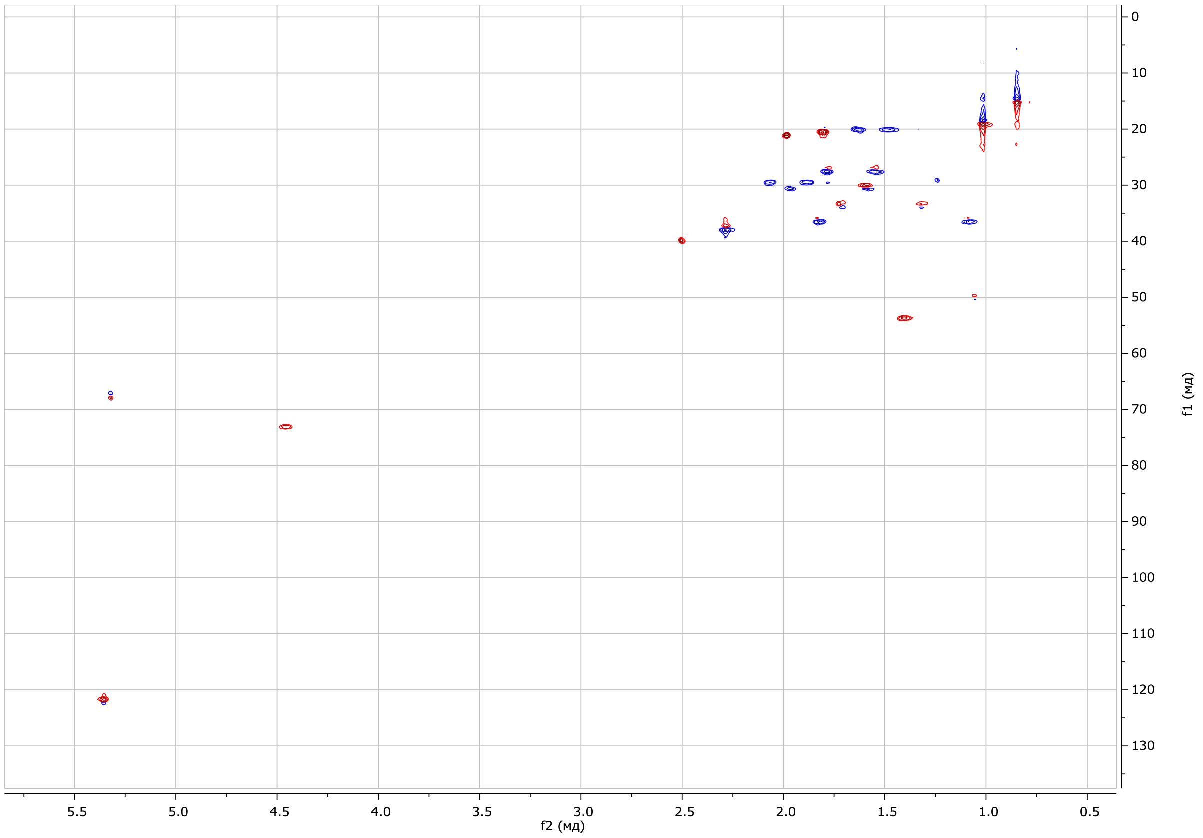

^{1}H-^{13}C HMBC NMR (DMSO-d_6) spectrum of

3β-acetoxy-16-(4,6-dimethyl-1,2-dihydro-1,3,5-triazi-2-il)-17-chlorandrosta-5,16-dien (4b)

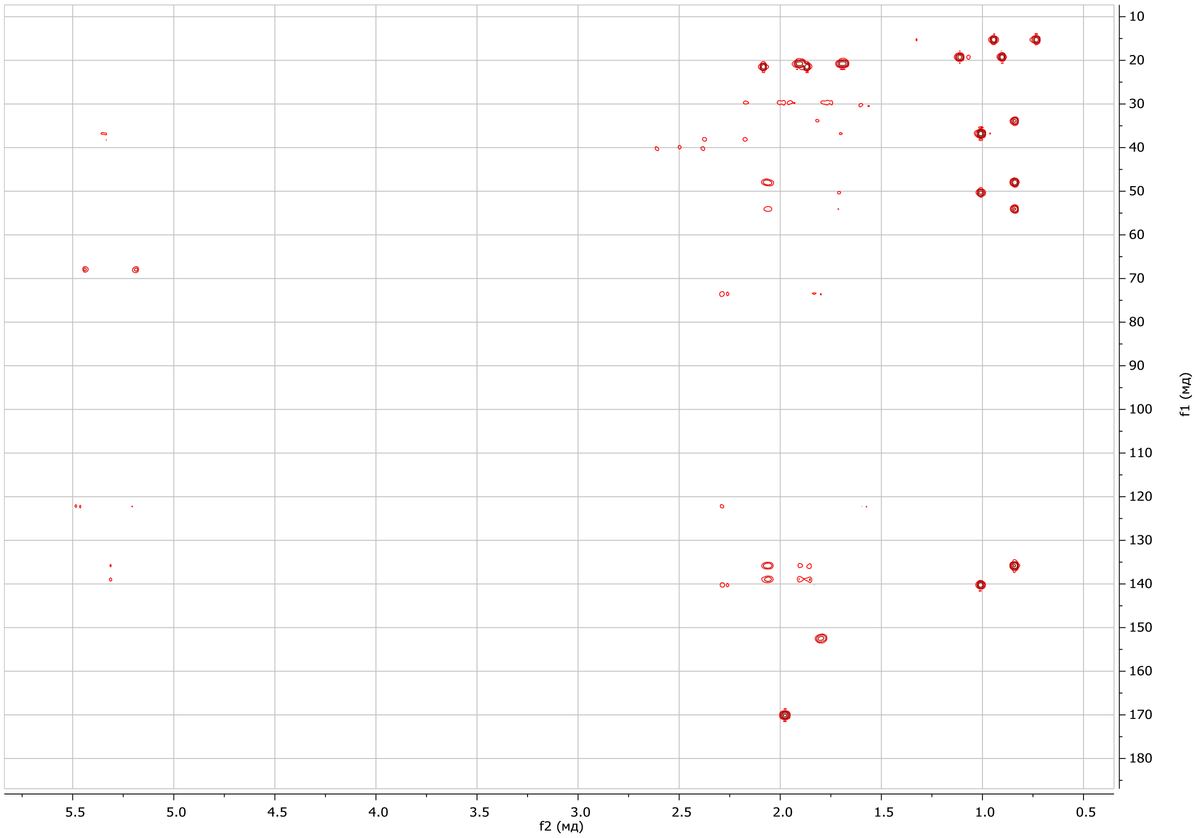

^{1}H NMR (DMSO-d$_6$, 600 MHz) spectrum of

17β-hydroxy-2-(4,6-dimethyl-1,2-dihydro-1,3,5-triazin-2-il)-3-chlor-5α-androstane (4c)

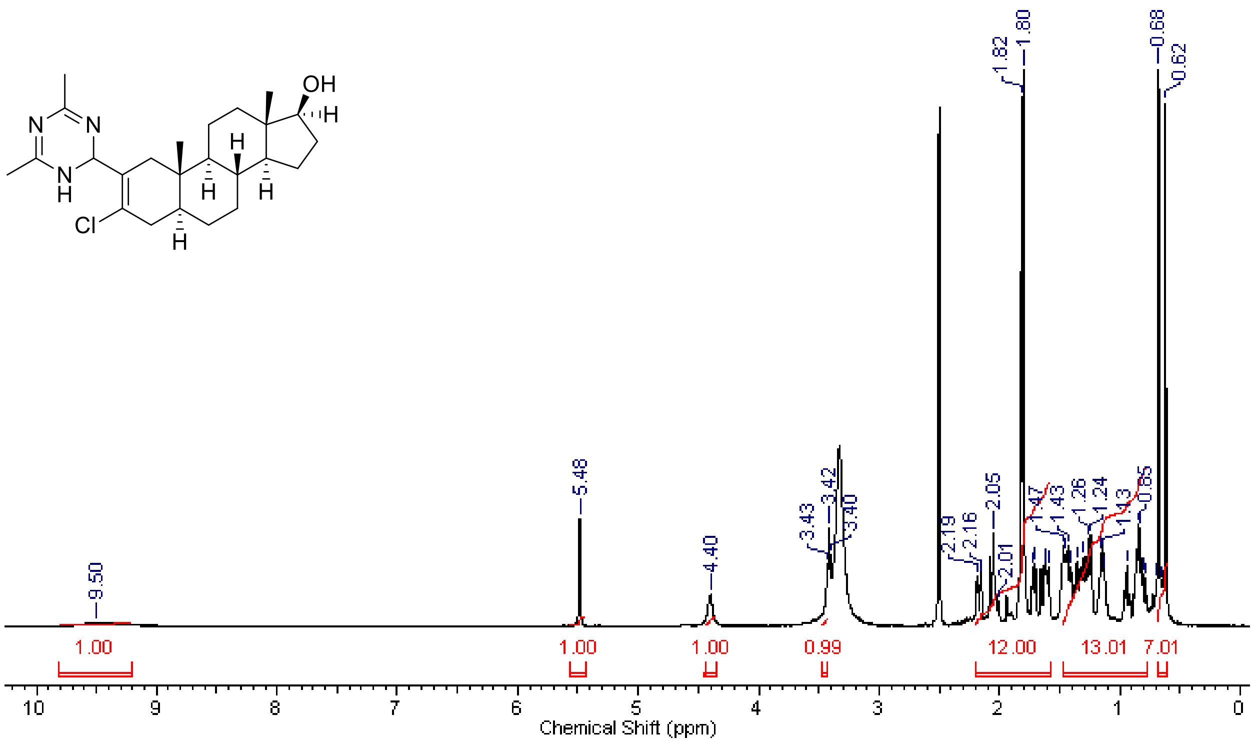

^{13}C NMR (DMSO-d$_6$, 150 MHz) spectrum of

17β-hydroxy-2-(4,6-dimethyl-1,2-dihydro-1,3,5-triazin-2-il)-3-chlor-5α-androstane (4c)

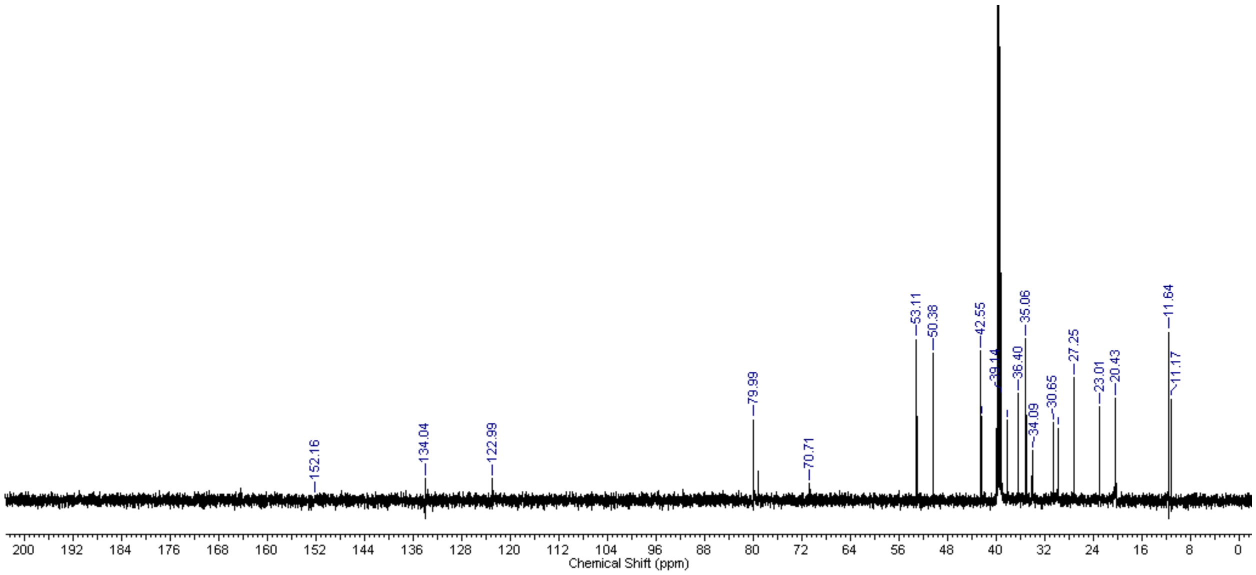

^{1}H-^{13}C HSQC NMR (DMSO-d$_6$) spectrum of

17β-hydroxy-2-(4,6-dimethyl-1,2-dihydro-1,3,5-triazin-2-il)-3-chlor-5α-androstane (4c)

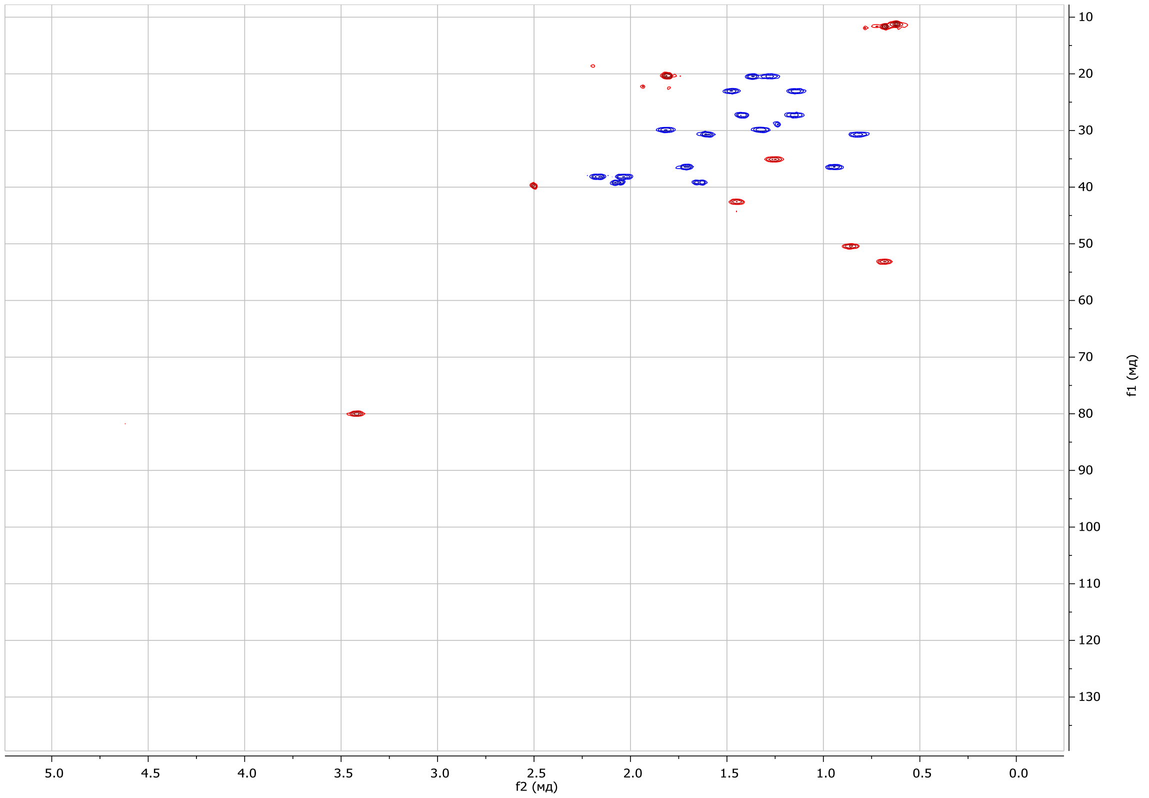

^{1}H-^{13}C HMBC NMR (DMSO-d$_6$) spectrum of

17β-hydroxy-2-(4,6-dimethyl-1,2-dihydro-1,3,5-triazin-2-il)-3-chlor-5α-androstane (4c)

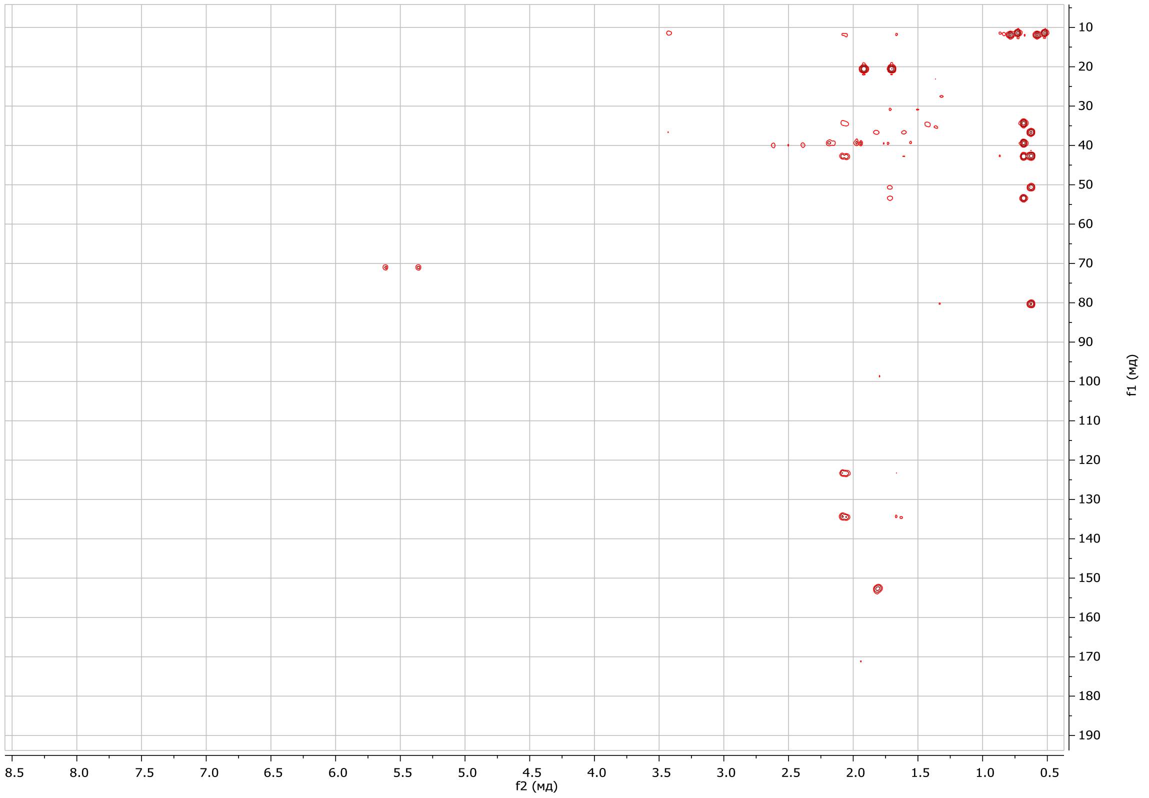

Biology

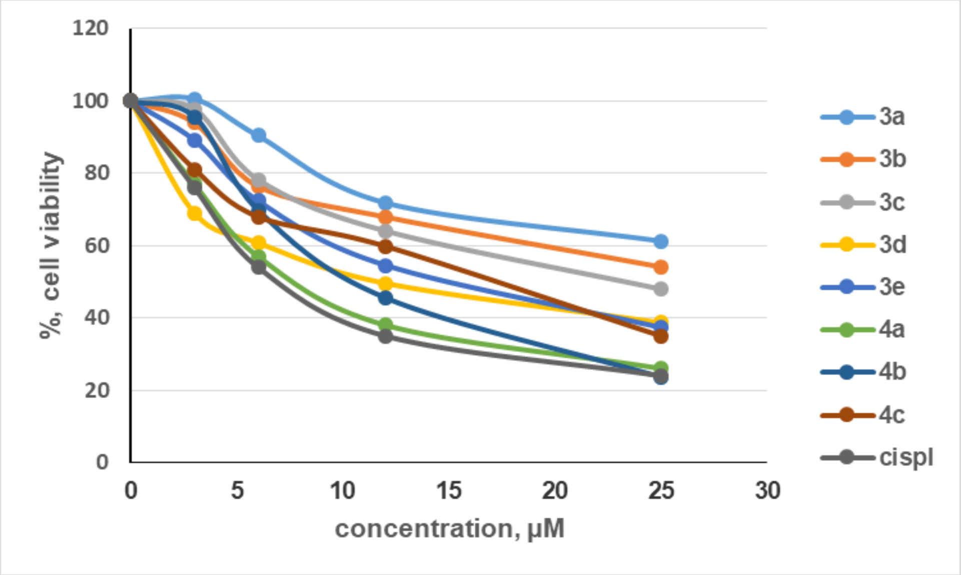

Figure S1. Antiproliferative activity of the synthesized heterosteroidal compounds and cisplatin against MCF-7 cells. Cisplatin (cislp) was used as reference drug. Cells were treated with compounds for 72 h and then the viability was assessed by MTT assay. Results were expressed as mean of 3 independent experiments.

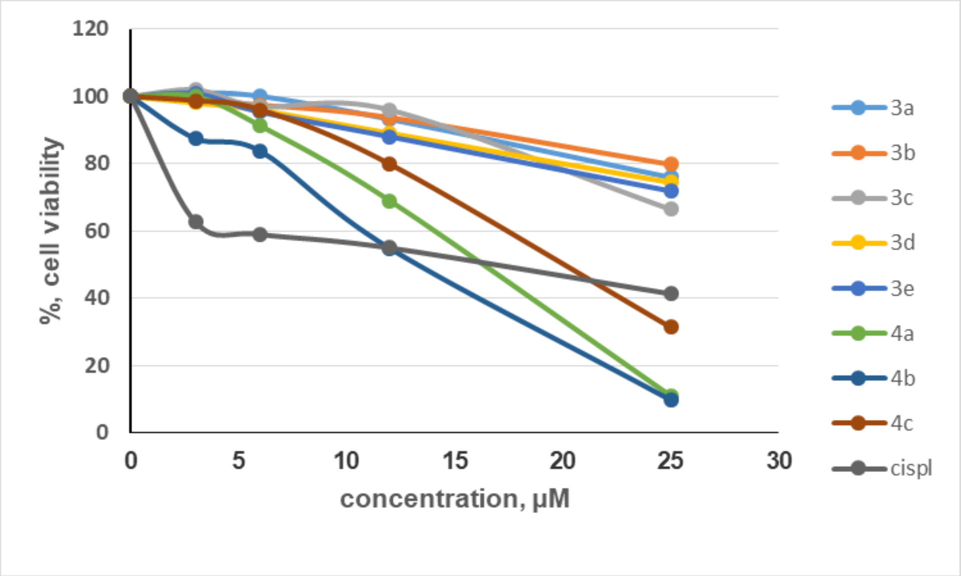

Figure S2. Antiproliferative activity of the synthesized heterosteroidal compounds and cisplatin against MDA-MB231 cells.

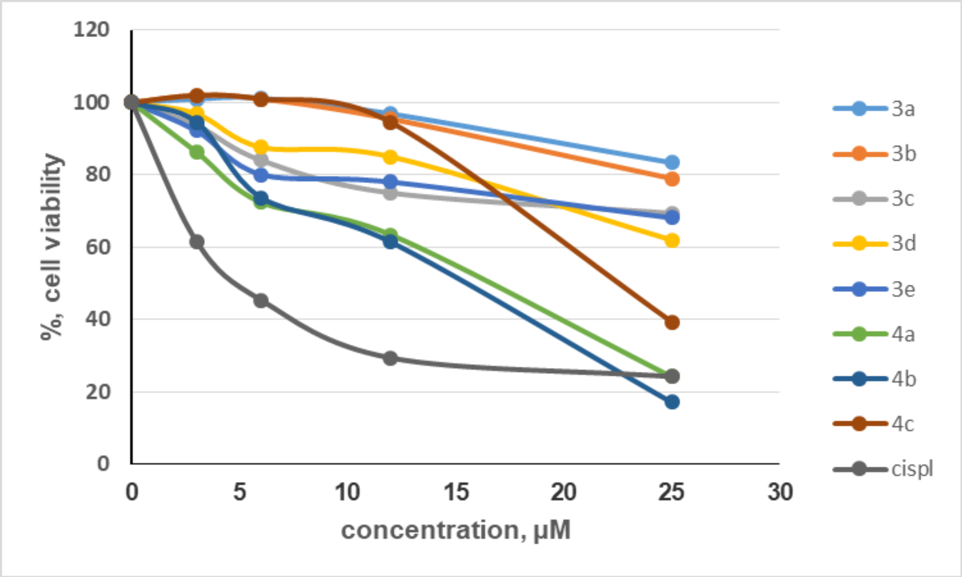

Figure S3. Antiproliferative activity of the synthesized heterosteroidal compounds and cisplatin against PC3 cells.

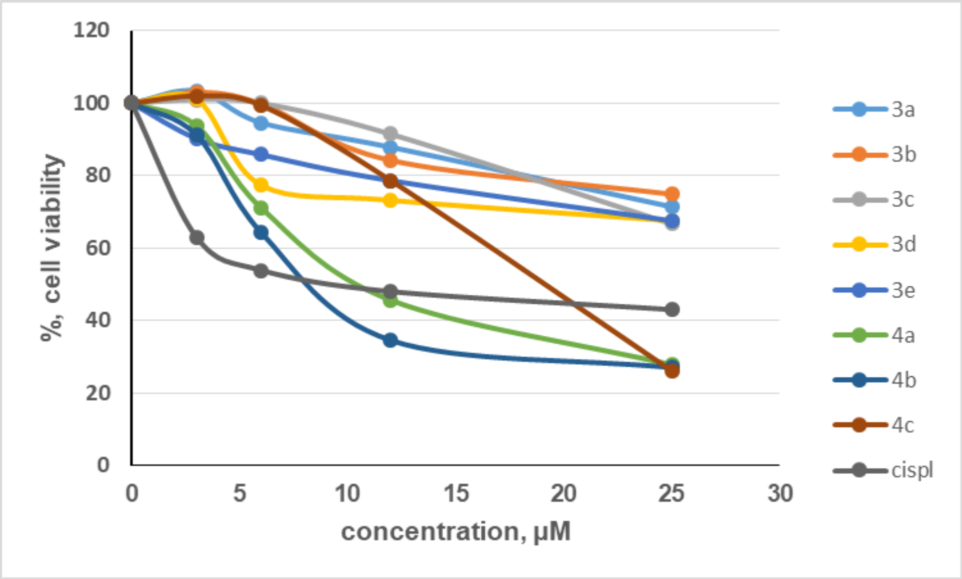

Figure S4. Antiproliferative activity of the synthesized heterosteroidal compounds and cisplatin against 22Rv1 cells.

Table **S1**. **Agonist effects of compounds on ERE-TK-luciferase activity in MCF-7 cells.** The transcriptional activation of reporter was assessed using Promega Luciferase assays. Cells were treated with compounds at 10 nM concentrations. 17β-estradiol (E2) was used as the reference ERα agonist. The results were expressed as the ratio of the firefly luciferase activity to the β-galactosidase activity, and the luciferase activity was relative to that of the cells treated with E2 which was set as 100.

Entry	Luciferase activity, rel. units
**DMSO**	2.0 ± 0.3
**3a**	6.9 ± 0.7
**3b**	11.4 ± 1.2
**3c**	2.1 ± 0.2
**3d**	2.0 ± 0.2
**3e**	2.1 ± 0.2
**4a**	2.2 ± 0.3
**4b**	2.0 ± 0.2
**4c**	1.9 ± 0.3
**E2**	100 ± 11
